# Three-Dimensional Noncovalent Interaction Network within [NpO_2_Cl_4_]^2–^ Coordination Compounds: Influence on Thermochemical and Vibrational Properties

**DOI:** 10.1021/acs.inorgchem.3c02502

**Published:** 2023-10-10

**Authors:** Harindu Rajapaksha, Grant C. Benthin, Dmytro V. Kravchuk, Haley Lightfoot, Sara E. Mason, Tori Z. Forbes

**Affiliations:** † Department of Chemistry, University of Iowa, Iowa City, Iowa 52242, United States; ‡ Center for Functional Nanomaterials, Brookhaven National Laboratory, Upton, New York 11973, United States

## Abstract

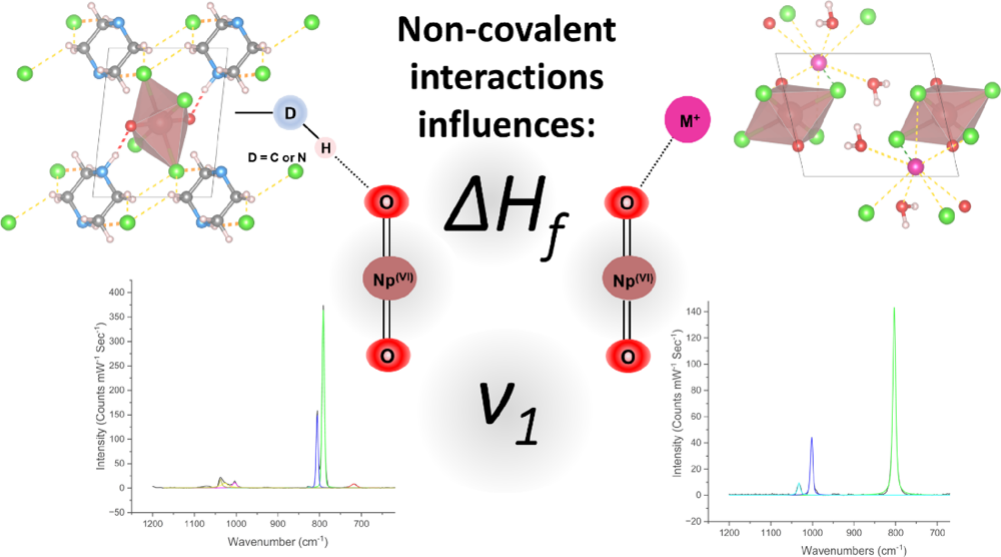

Noncovalent interactions (NCIs) can influence the stability and chemical properties of pentavalent and hexavalent actinyl (AnO_2_^+/2+^) compounds. In this work, the impact of NCIs (actinyl–hydrogen and actinyl–cation interactions) on the enthalpy of formation (Δ*H*_f_) and vibrational features was evaluated using Np(VI) tetrachloro compounds as the model system. We calculated the Δ*H*_f_ values of these solid-state compounds through density functional theory+ thermodynamics (DFT+ T) and validated the results against experimental Δ*H*_f_ values obtained through isothermal acid calorimetry. Three structural descriptors were evaluated to develop predictors for Δ*H*_f_, finding a strong link between Δ*H*_f_ and hydrogen bond energy (*E*_H_^total^) for neptunyl–hydrogen interactions and total electrostatic attraction energy (*E*_electrostatic_^total^) for neptunyl–cation interactions. Finally, we used Raman spectroscopy together with bond order analysis to probe Np=O bond perturbation due to NCIs. Our results showed a strong correlation between the degree of NCIs by axial oxygen and red-shifting of Np=O symmetrical stretch (ν_1_) wavenumbers and quantitatively demonstrated that NCIs can weaken the Np=O bond. These properties were then compared to those of related U(VI) and Np(V) phases to evaluate the effects of subtle differences in the NCIs and overall properties. In general, the outcomes of our study demonstrated the role of NCIs in stabilizing actinyl solid materials, which consequently governs their thermochemical behaviors and vibrational signatures.

## Abstract 2

Noncovalent interactions within solid-state neptunyl tetrachloro compounds were analyzed using theoretical and experimental methods. We found that these interactions have a direct influence on the formation enthalpy (Δ*H*_f_) and neptunyl vibrational signatures of the solid phases.

## 1 Introduction

High valent actinides, particularly the pentavalent and hexavalent states, display a unique chemistry that influences the overall properties of the solid-state compounds.^1−3^ While multiple oxidation states exist for the actinide elements, the penta- and hexavalent forms are most prevalent for uranium (U) and neptunium (Np).^3,4^ In these higher valent oxidation states, the actinide element exists within the actinyl cation, AnO_2_^*n*+^ (*n* = 1 for pentavalent and *n* = 2 for hexavalent forms).^5,6^ The actinyl cation is linear and contains two strong bonds to oxygen atoms to achieve complete bonding saturation in the axial plane.^3,6^ Additional ligand coordination to the actinyl cation occurs through the equatorial plane, resulting in tetragonal, pentagonal, or hexagonal bipyramidal complexes.^7,8^

While the structural chemistry of the actinyl cation is similar between U and Np, subtle differences in the electronic structure impact the overall chemistry of the system, particularly for noncovalent interactions (NCIs) that take place in the second coordination sphere environment.^6,9,10^ Strong An=O bonding reduces the Lewis basicity of the axial oxygen,^11^ but the equatorial ligands increase electron density at the An center, weakening the An=O bond^11,12^ and promoting a range of NCIs. The interaction between the actinyl oxo and neighboring groups can be classified into various forms, including actinyl–cation (ACIs),^13,14^ actinyl–hydrogen (AHIs),^15,16^ actinyl–halogen (AXIs),^10,12^ and actinyl–actinyl interactions (AAIs).^17^ ACIs, AHIs, and AXIs are found in both hexa- and pentavalent species; however, AAIs are more common in pentavalent species.^17,18^ When compared to the hexavalent cation (AnO_2_^2+^), the pentavalent cation (AnO_2_^+^) has greater electron density at the An^n+^ center, weakening the An=O bond and increasing axial oxygen participation in NCIs.^6,19^ Because of the reasons outlined above, NpO_2_^2+^ is projected to have a higher tendency to produce NCIs than UO_2_^2+^. However, only a minor increase in electron density surrounding the oxo ligands has previously been described, implying a comparable ability to make NCIs.^10,20^

It is important to have model compounds to systematically analyze NCIs and their effects on structural, vibrational, and redox behavior. The actinyl tetrachloride system has previously been utilized as a model system due to its ability to form a range of solid-state compounds with high reproducibility.^10,13,15,16,21^ This system has been widely utilized by the Cahill group to develop rational approaches to the design of U(VI) hybrid materials and to explore NCIs. In addition, Cahill et al. conducted preliminary investigations into the thermochemical properties of actinyl systems and their correlation with NCIs. In this work, they found a relationship between the formation enthalpies and the protonation of the charge-balancing organic cations.^22^ In addition, Surbella et al. extended this system to evaluate the structural features and NCIs of transuranic complexes, including [AnO_2_Cl_4_]^2–^ (An = Np and Pu) systems.^10^ They report a slight increase in the Lewis basicity of the axial oxo ligands across the period and conclude that NCIs dictate the arrangement of molecular units in the crystalline lattice. Furthermore, perturbation of the An=O bond by NCIs has been shown to result in red-shifting of the symmetrical stretch (ν_1_) and the activation of additional vibrational bands.^13,15,18^ However, a systematic evaluation of the relationship between NCIs and overall stability and vibrational characteristics has not been conducted, particularly for Np(VI), due to the inherent difficulties in working with transuranic materials.

**Figure 1:**
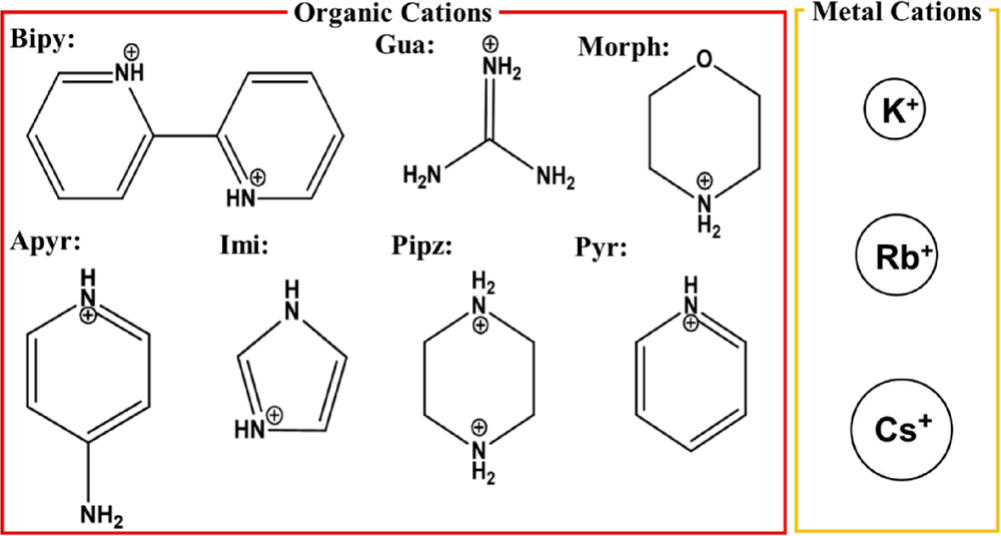
Seven Np(V) tetrachloro compounds were synthesized for this study using Bipy, 2,2′-bipyridinium (Np(VI)-Bipy); Gua, guanidium (Np(VI)-Gua); Morph, morpholinium (Np(VI)-Morph); Apyr, 4-aminipyridium (Np(VI)-Apyr); Imi, imidazolium (Np(VI)-Imi); Pipz, piperazinium (Np(VI)-Pipz); and Pyr, pyridinium (Np(VI)-Pyr), as charge-balancing cations. Metal cations (K^+^, Rb^+^, and Cs^+^) were also used as counterions in the formation of three Np(VI) tetrachloride coordination compounds (Np(VI)-K, Np(VI)-Rb, and Np(VI)-Cs).

In this study, we systematically evaluated the impact of NCIs on the thermodynamics (Δ*H*_f_) and vibrational signatures of neptunyl tetrachloro compounds, with a specific focus on neptunyl–hydrogen and neptunyl–cation interactions. Herein, we synthesized seven Np(VI) tetrachloride compounds (including six novel compounds) that engage in neptunyl–hydrogen interactions and three additional phases (including two novel compounds) that engage in neptunyl–cation interactions (Figure 1). Measurements of Δ*H*_f_ necessitated ∼30 mg of pure materials for each measurement, which is difficult to obtain for Np-237, and therefore, only two systems (Np(VI)-Pipz and Np(VI)-Pyr) were subjected to analysis. To expand our data set, we introduced a DFT+ thermodynamics (DFT+ T) method as a reliable method in calculating the formation enthalpies (Δ*H*_f_) of neptunyl halide systems.^23,24^ We recently employed periodic DFT to examine the effect of NCIs on the vibrational and thermochemical properties of solid-state U(VI) halide compounds and demonstrated its accuracy compared to experimental values.^23^ Utilizing this DFT methodology enabled us to evaluate the relative stability of all of the structurally characterized compounds, evaluate structural descriptors that can be used to predict compound stability, and explore the relationship between bond order and the energy of the neptunyl symmetrical stretch (ν_1_). This approach also enabled us to explore subtle differences between U(VI), Np(V), and Np(VI) tetrachloro systems to provide a systematic assessment of the role of NCIs in these materials.

## 2 Experimental Methods

### 2.1 Synthesis and Characterization of Materials

*Caution: Neptunium-237 (*^*237*^*Np) is an alpha emitter with a half-life of 2.14 million years. Its daughter product, protactinium-233 (*^*233*^*Pa), is a highly radioactive beta emitter with a half-life of 26.9 days. Research with this isotope is restricted to specialized laboratories and must be handled under the appropriate regulatory controls.*^*237*^*Np used in this study was purchased from the Oak Ridge National Laboratory Isotope Production Program.* Synthesis of Np(VI) tetrachloride coordination compounds was performed by mixing 0.27 M Np(VI) in 2 M HCl with the appropriate charge-balancing cation (bipyridinium (Bipy), piperazinium (Pipz), guanidinium (Gua), morpholinium (Morph), 4-aminopyridinium (Apyr), imidazolium (Imi), cesium (Cs), rubidium (Rb), or potassium (K)) (Figure 1). Slow evaporation led to the formation of large yellow crystals that were harvested from the mother liquor. Section 1.1 of the Supporting Information contains detailed information about the synthesis of the material. Single-crystal X-ray diffraction was utilized to determine the structural features of the material (Supporting Information, Sections 1.2 and 1.3), and powder X-ray diffraction assessed the purity of the crystalline material used for calorimetric measurements (Supporting Information, Section 2.1, Figure S1).

### 2.2 Calorimetry

Two Np(VI) phases (Np(VI)-Pipz and Np(VI)-Pyr) were selected, on the basis of their high overall yield and purity, for calorimetric measurements to verify the accuracy of theoretical calculations. Solvation enthalpies for these two compounds were measured using a Setaram Calvet C80 calorimeter at 25.0 ± 0.1 °C and at atmospheric pressure. Between 10 and 15 mg of pure samples was mixed with 1.000 mL of HCl solution (2 N HCl in H_2_O), and the isothermal heat transfer rate was recorded and compared to the reference (Δ*H*_sol_ values are provided in the Supporting Information, Section 4.1, Table S7). To ensure proper system equilibration, a baseline variation of 0.05 mW was maintained before and after mixing for 15 min. Baseline subtraction and peak integration were performed using the Calisto Processing software.

### 2.3 Computational Details

The Vienna Ab initio Simulation Package (VASP) was used for all DFT calculations.^25−27^ To represent exchange-correlation energy, the generalized gradient approximation of Perdew–Burke–Ernzerhof was utilized.^28^ Atoms were represented using projector augmented wave^29,30^ pseudopotentials. A place wave basis set cutoff of 550 eV and gamma-centered k-grid^31^ of at least 5 × 3 × 3 was used. Without symmetry restrictions, all structures were subjected to comprehensive geometry optimizations, and the forces and total energy converged to within 1 meV Å^–1^ and 1 × 10^–8^ eV, respectively. Following the technique of Dudarev et al.,^64^ a Hubbard *U* correction of 3.75 eV was added to the neptunium *f* states. Benchmarking of a Hubbard *U* correction and magnetic orientation is also discussed in the Supporting Information, Sections 3.1 and 3.2. In all DFT computations, the van der Waals dispersion correction methods (DFT-D3), including the Becke–Johnson damping term,^32^ were utilized. The Phonopy software was used to conduct vibrational calculations by using the finite-displacement approach.^33^ Bond orders (BOs) were estimated using the Chargemol program’s Density-Derived Electrostatic and Chemical 6 (DDEC6) method.^34−37^

## 3 Results and Discussion

### 3.1 Structural Description

#### Neptunyl–Hydrogen Compounds

**Figure 2:**
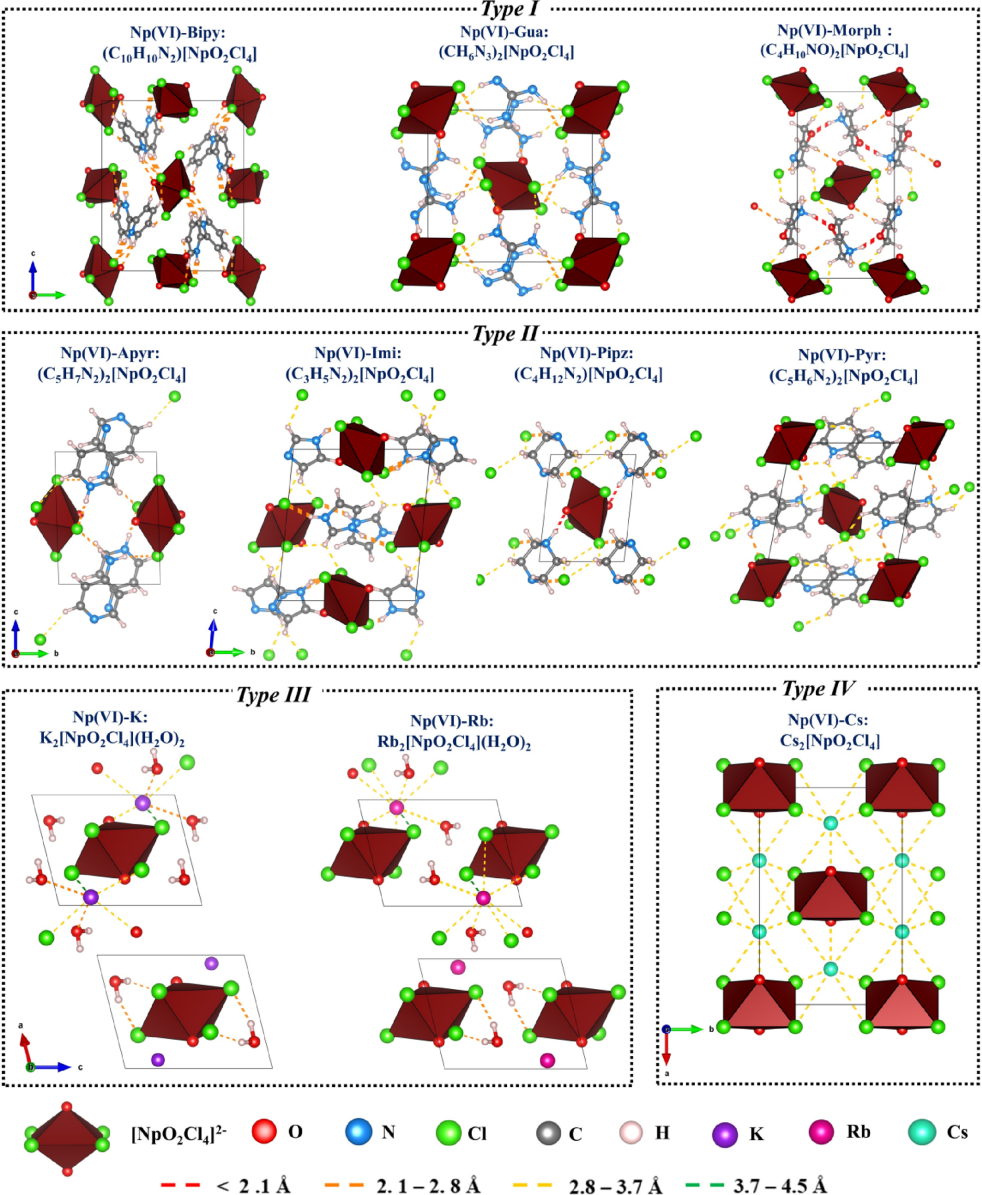
Structures of the crystallized neptunyl hybrid materials, organized by type, as defined in the text. NCI network projected over the DFT optimized unit cells. The color of the interaction indicates the distance. (VESTA visualization files are provided as a part of the Supporting Information.) A legend is provided below the image, which defines the molecular units, atoms, and hydrogen bond distances. The criterions used in the identification of NCIs are borrowed from our previous work.^23^

Pairing [NpO_2_Cl_4_]^2–^ unit with seven organic balancing cations (Figure 1) resulted in the crystallization of the neptunyl hybrid materials (Figure 2) where six of them are novel compounds (crystallographic data are provided in the Supporting Information, Section 1.3, Tables S1 and S2). The exception to this is the Np(VI)-pyr compound, which was previously reported by Surbella et al.^10^ These compounds are isostructural to previously reported [UO_2_Cl_4_]^2–^ structures^18,23^ and can be characterized into two general structure types based on the space group of the crystalline lattice and types of NCIs (Table 1).

**Table 1:** Description of the NCI, Space Group, and Compounds Belonging to Each Structure Type

	**structure type**			
	**type I**	**type II**	**type III**	**type IV**
type of neptunyl interaction	neptunyl–hydrogen		neptunyl–cation	
space group	*P*2_1_/*n*	*P*-1	*P*-1	*C*2/*m*
compounds	Np(VI)-Bipy	Np(VI)-Apyr	Np(VI)-K	Np(VI)-Cs
	Np(VI)-Gua	Np(VI)-Imi	Np(VI)-Rb	
	Np(VI)-Morph	Np(VI)-Pipz		
		Np(VI)-Pyr		

Type I structures (Figure 2) crystallize in the *P*2_1_/*n* space group. On average, the Np=O distances for the axial oxygen of the neptunyl cation are 1.757, 1.739, and 1.754 Å for Np(VI)-Bipy, Np(VI)-Morph, and Np(VI)-Gua, respectively. The Np–Cl distances for these three complexes are 2.654, 2.656, and 2.661 Å, respectively. Np(VI)-Bipy contains 2,2′-bipyridinium cations that are linked by π–π stacking in the [100] direction. In contrast, Np(VI)-Morph has supramolecular chains of morpholinium, linked by hydrogen bonds, that extend in the [010] direction. Though Np(VI)-Morph is isostructural to the equivalent U(VI) phase,^23^ it differs structurally from the related Np(V) compound as there are additional interactions between the neptunyl oxo and the morpholinium cation.^15^ In Np(VI)-Gua, the guanidium ions form extended chains in the [100] direction through hydrogen bonding with [NpO_2_Cl_4_]^2–^ units. Type II structures (Figure 2) crystallize in the *P*-1 space group, with Np=O bond distances ranging from 1.752 to 1.769 Å, and Np–Cl distances are similar to those observed in type I. The type II compound, Np(VI)-Apyr, contains a supramolecular chain of 4-aminopyridine cations extending in the [100] direction that are linked by π–NH_2_^+^ interactions (3.386 Å). Other remaining structures do not show clear evidence of extended supramolecular interactions between the charge-balancing cations. Both type I and type II structures have hydrogen bonds between the organic cation and the [NpO_2_Cl_4_]^2–^ unit that vary in type and strength where four motifs (N–H···Cl_eq_, C–H···Cl_eq_, N–H···O_yl_, and C–H···O_yl_) were observed in the crystallized phases. These NCIs identified in the Np(VI) compounds are similar to our previous report on [UO_2_Cl_4_]^2–^ hybrid materials.^23^ A detailed description of the hydrogen bonding interactions in the seven neptunyl systems is provided in the Supporting Information, Section 6.

#### Neptunyl–Cation Compounds

Neptunyl tetrachloride coordination complexes crystallized with alkali metal cations can also be classified into two structure types based on the space group. Two of the three compounds have not been previously reported (Np(VI)-K and Np(VI)-Rb), and all three neptunyl–cation compounds are isostructural to the corresponding U(VI) compounds.^24^ The K^+^ and Rb^+^ materials are classified as type III compounds (Figure 2) because they crystallize in the space group *P*-1, have actinyl–cation interactions, and are isostructural to each other. The Np=O and Np–Cl bond lengths are similar in both structures at 1.757 and 2.646 Å, respectively. In each compound, the alkali metal cation interacts with the neptunyl oxo, equatorial chloride anions, and water molecules located in the unit cell (Figure 2). Np(VI)-Cs is a type IV compound (Figure 2) that crystallizes in the space group *C*2/*m* and has been reported earlier by Wilkerson et al.^38^ This compound displays bond distances similar to those of the other two compounds (Np=O distance of 1.764 Å). In addition, Np(VI)-Cs is isostructural to the analogous U(VI) structure but differs structurally from the Np(V) phase^39^ where the Cs^+^ strongly interacts with both Np(V)O_2_^+^ oxo groups and equatorial chloride. A detailed description of the cation interactions on these three systems is provided in the Supporting Information, Section 6.

### 3.2 Experimental and Computational Thermochemistry

In our previous studies, we effectively employed a DFT+ T methodology to compute the Δ*H*_f_ of uranyl solid-state complexes and observed good agreement between theoretical predictions and experimental results.^23,24^ In the current work, we successfully adopted the DFT+ T approach to calculate the Δ*H*_f_ of neptunyl systems. The overall formation reactions used in the computation approach for neptunyl complexes are given in eqs 1–5. The full thermocycles used in experimental and theoretical Δ*H*_f_ determination are provided in the Supporting Information, Sections 4.1 and 4.2 and Table 2.
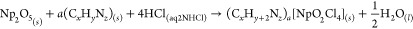
1
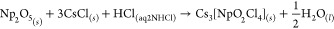
2
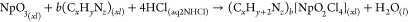
3
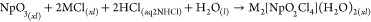
4
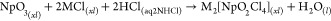
5

**Figure 3:**
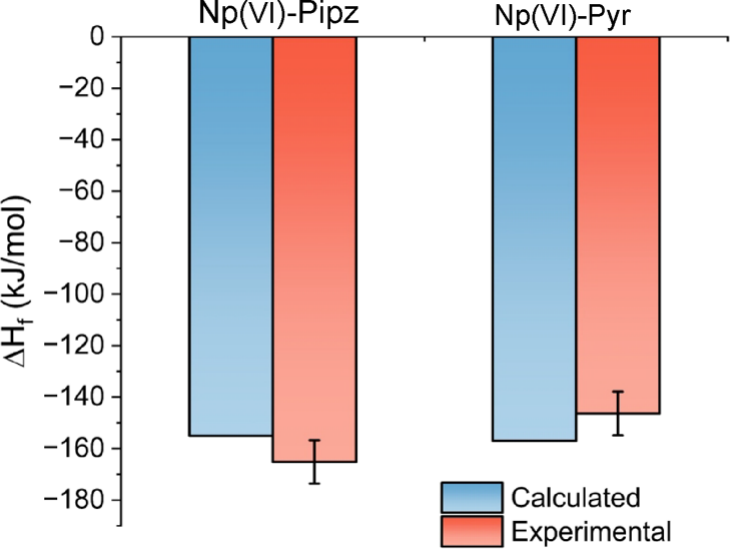
Chart of the calculated and experimental formation enthalpy (Δ*H*_f_) of Np(VI)-Pipz and Np(VI)-Pyr systems.

Calculated Δ*H*_f_ values were comparable to those of other actinyl hybrid systems and agreed well with the experimental values obtained from the calorimetry (Figure 3). The Δ*H*_f_ values for the hybrid Np(VI) system ranged from −251.40 to −103.36 kJ/mol, which was comparable to the calculated U(VI) system (−217.74 to −81.37 kJ/mol).^23^ Theoretical Δ*H*_f_ values for Np(VI)-Pipz and Np(VI)-Pyr were compared to experimental values, resulting in percent errors of −6.12 and +7.96%, respectively. These percent errors are consistent with previously reported examples for the Δ*H*_f_ for metal oxides^40−44^ and uranyl(VI) hybrid materials.^23^ Based on the fact that the DFT+ T technique utilized here exhibits a high degree of agreement with experimental results, despite the complexity of the aqueous chemistry effects shown in the isothermal calorimetry data, we believe that it is clearly an appropriate methodology to use to generate predictions and detect patterns.

**Table 2:** Calculated Values for the Formation Enthalpy (Δ*H*_f_) for the Neptunyl Materials and Experimental Formation Enthalpy (Δ*H*_f_) for Np(VI)-Pipz and Np(VI)-Pyr at 25 °C and Atmospheric Pressure^a^

**compound**	**calculated Δ***H*_**f**_(kJ/mol)	**experimental Δ***H*_**f**_(kJ/mol)	**absolute error per atom** kJ/mol)
Np(V)-Morph	–209.84		
Np(V)-Pipz	–202.31		
Np(V)-Cs	–24.17		
Np(VI)-Bipy	–103.32		
Np(VI)-Gua	–203.79		
Np(VI)-Morph	–251.40		
Np(VI)-Apyr	–176.75		
Np(VI)-Imi	–117.67		
Np(VI)-Pipz	–155.08	–165.19 ± 8.43	0.40
Np(VI)-Pyr	–156.94	–146.37 ± 8.50	0.17
Np(VI)-K	–100.81		
Np(VI)-Rb	–98.04		
Np(VI)-Cs	–93.34		

a The uncertainty of experimental Δ*H*_f_ was calculated as 2σ of the mean.

After validating the DFT methodology, we can now compare the U(VI) and Np(VI) hybrid systems. When the computed Δ*H*_f_ of uranyl(VI) tetrachloride hybrid complexes from our prior study are compared to those of the analogous neptunyl(VI) phases, all Np(VI) hybrid compounds have more exothermic Δ*H*_f_ values.^23^ The enthalpy of MO_3(s)_ (M = U or Np) and the enthalpy of the hybrid complex itself are the only two variables that change between the uranyl(VI) and neptunyl(VI) thermocycles. Because the shift occurs consistently across all hybrid systems, it may be attributed to MO_3(s)_ (M = U or Np), where NpO_3(s)_ (H = −34.86 eV) has lower stability than UO_3(s)_ (H = −35.07 eV) that leads to less exothermic Np(VI) enthalpies as compared to U(VI) values.^45^

The DFT+ T methodology provides a means for studying the stability of Np(V) phases in a pure form, which is challenging using experimental approaches due to the redox behavior of Np. The Δ*H*_f_ of two Np(V) hybrid complexes, morpholinium and piperazinium Np(V) tetrachloro compounds (Np(V)-Morph and Np(V)-Pipz), were calculated at Δ*H*_f_ = −290.84 and −202.31 kJ/mol, respectively. These values are of greater magnitude than those observed for Δ*H*_f_ of Np(VI)-Morph (−251.40 kJ/mol) and Np(VI)-Pipz (−156.94 kJ/mol). The larger Δ*H*_f_ values of the Np(V) phases can be related to the increased number of organic cations in the formula unit (e.g., Np(V)-Morph, (C_4_H_10_NO)_3_[NpO_2_Cl_4_], and Np(VI)-Morph, (C_4_H_10_NO)_2_[NpO_2_Cl_4_]).

Moving to the Np(VI) tetrachloro compounds that contain alkali metal cations, we observe Δ*H*_f_ values that are smaller in magnitude than those of the hybrid systems and are different from those of the related U(VI) compounds. The Δ*H*_f_ for the Np(VI) compounds containing K^+^, Rb^+^, and Cs^+^ are all relatively similar to values of −100.81, −98.04, and −93.36 kJ/mol, respectively. Thermodynamic parameters of related U(VI) compounds were previously calculated using the DFT+ T methodology and can be compared to the analogous Np(VI) phase.^24^ The results generally show that the formation of compounds from NpO_2_^+2^ with K^+^ and Rb^+^ is less exothermic than the isostructural UO_2_^+2^ phases with values of −156.89 and −167.63 kJ/mol.^24^ In contrast, the Cs^+^ phases do not follow this trend, with Δ*H*_f_ values for the U(VI) phase at more endothermic values (−40.40 kJ/mol) compared to those of the related Np(VI) phase. In addition, the Np(V)-Cs compound was also calculated at −24.12 kJ/mol. This enables us to determine the relative values of the Δ*H*_f_ as follows: NpO_2_^+^ < UO_2_^+2^ < NpO_2_^+2^ for the hybrid [AnO_2_Cl_4_]^3–/2–^ systems.

### 3.3 Descriptors of Δ*H*_f_

#### 3.3.1 Neptunyl–Hydrogen Interactions

**Figure 4:**
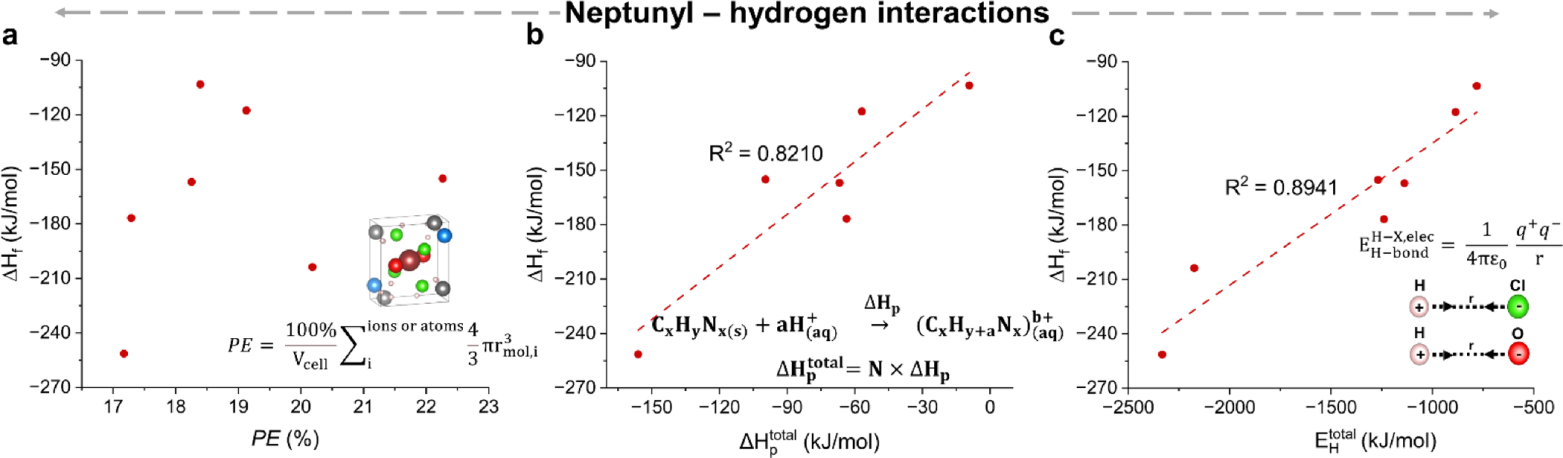
Correlation plots for neptunyl–hydrogen systems. (a) Δ*H*_f_ versus packing efficiency (PE) for Np(VI) hybrid compounds plotted with the equation used to calculate the PE imbedded in the figure. (b) Plot of Δ*H*_f_ vs *H*_p_^total^ with the general reaction for the protonation of the organic base and *H*_p_^total^ calculation with solvation enthalpy and number of cations in the formula unit. (C) Δ*H*_f_ plotted vs *E*_H_^total^, where *E*_H_^total^ was calculated electrostatically. Both neptunyl oxo groups and equatorial chloride anions are considered to be hydrogen bond acceptors. The full definitions of the embedded equations in Figure 4a–c are given in eqs 6, 7, and 8, respectively.

To further understand the trends in the enthalpy values for the actinyl tetrachloro coordination compounds, we turn to the exploration of structural descriptors. In our previous work, we have discussed three descriptors of Δ*H*_f_ in U(VI) hybrid materials: packing efficiency (PE), total protonation energy of the charge-balancing organic cation (*H*_p_^total^) and hydrogen bond energy (*E*_H_^total^).^23^ In the current work, we evaluated these three descriptors on neptunyl systems (Figure 4).

#### Packing Efficiency (PE)

Packing efficiency was determined by eq 6, as defined by Kitaigorodsky.^46^ Here, *V*_cell_ is the volume of the experimental unit cell, and *r*_mol_ is the covalent radii of C, H, N, and O of the organic cation and ionic radii of K^+^, Rb^+^, Cs^+^, Cl^–^, Br^–^, and O^2–^ of the neptunyl cation and Np^5/6+^.^47,48^
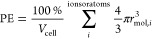
6When the values of Δ*H*_f_ were plotted against PE, we did not observe any clear trend (Figure 4a), indicating that PE is not a primary descriptor of Δ*H*_f_ neptunyl(VI) hybrid complexes. This result matches observations in uranyl(VI) hybrid materials^23^ and hydrogen-bonded cocrystals^49,50^ and suggests that the nature of the hydrogen bond plays a more important role in governing Δ*H*_f_.

#### Total Protonation Energy (*H*_p_^total^)

*H*_p_^total^ was determined by eq 7, where *N* is the number of organic cations in the formula unit and Δ*H*_sol_ is the solvation enthalpy of the organic cation (Table S8).
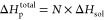
7

This descriptor of Δ*H*_f_ in hybrid materials was initially proposed by Cahill et al.^22^ and later confirmed by our work with U(VI) hybrid materials.^23^ The solvation/protonation enthalpy of guanidine was not measured during the study due to solid-state guanidine’s lack of availability; thus, the Np(VI)-Gua system was excluded from the regression analysis. Here, we identified a linear correlation between Δ*H*_f_ and *H*_p_^total^ with *R*^2^ = 0.8210 (Figure 4b), indicating that the more readily the organic bases protonate, the more stable the resultant neptunyl hybrid compound.

#### Hydrogen Bond Energy (*E*_H_^total^)

The *E*_H_^total^ descriptor was calculated by eqs 8 and 9 using the method proposed by Rajapaksha et al. for uranyl(VI) hybrid materials.^23^ Here, *q*^+^ and *q*^–^ are the atomic charges, *r* is the hydrogen interaction distance, and *z* is the number of formula units in the unit cell.
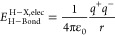
8
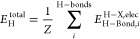
9

The calculated *E*_H_^total^ determines the effect of the three-dimensional (3-D) hydrogen bond network purely electrostatically (Table 3). Fitting the Δ*H*_f_ and *E*_H_^total^ to a linear regression model resulted in a *R*^2^ of 0.8941 (Figure 4c) and suggests that Np(VI) hybrid materials with a greater amount of hydrogen bonding within the unit cell are more likely to be stable. Comparing the *E*_H_^total^ of isostructural NpO_2_^2+^ and UO_2_^2+^ hybrid complexes shows that there is no systematic increase in hydrogen bonding strength in the NpO_2_^2+^ compounds.^10,15,23^ However, taking a closer look at the structure type reveals that the *E*_H_^total^ values are more exothermic for the Np(VI) compounds than the U(VI) compounds for type I compounds. Type II compounds display the opposite trend, with *E*_H_^total^ values for Np(VI) hybrid phases being more endothermic than the related U(VI) compounds. This could be because type I compounds have marginally more partial negative charges on equatorial chlorides than their uranyl counterparts, whereas type II compounds exhibit the opposite. But we were unable to determine why this pattern emerged from a particular crystal configuration.

**Table 3:** Calculated *E*_H_^total^ and Number of Hydrogen Bonds per Formula Unit of Neptunyl(VI) Hybrid Materials and *E*_H_^total^ of Uranyl(VI) Isomorph

**type**	**compound**	***E***_**H**_^**total**^(kJ/mol)	**number of H-bonds per formula unit**	***E***_**H**_^**total**^**of UO**_**2**_^**2+**^**isostructural compound** (kJ/mol)^23^
type I	Np(VI)-Bipy	–781.76	17	–764.34
	Np(VI)-Gua	–2174.47	22	–2115.62
	Np(VI)-Morph	–2332.97	32	–2228.54
type II	Np(VI)-Apyr	–1239.13	20	–1264.89
	Np(VI)-Imi	–886.15	15	–1257.07
	Np(VI)-Pipz	–1268.23	25	–1394.49
	Np(VI)-Pyr	–1137.98	25	

### 3.3.2 Neptunyl–Cation Interactions

#### Packing Efficiency (PE)

**Figure 5:**
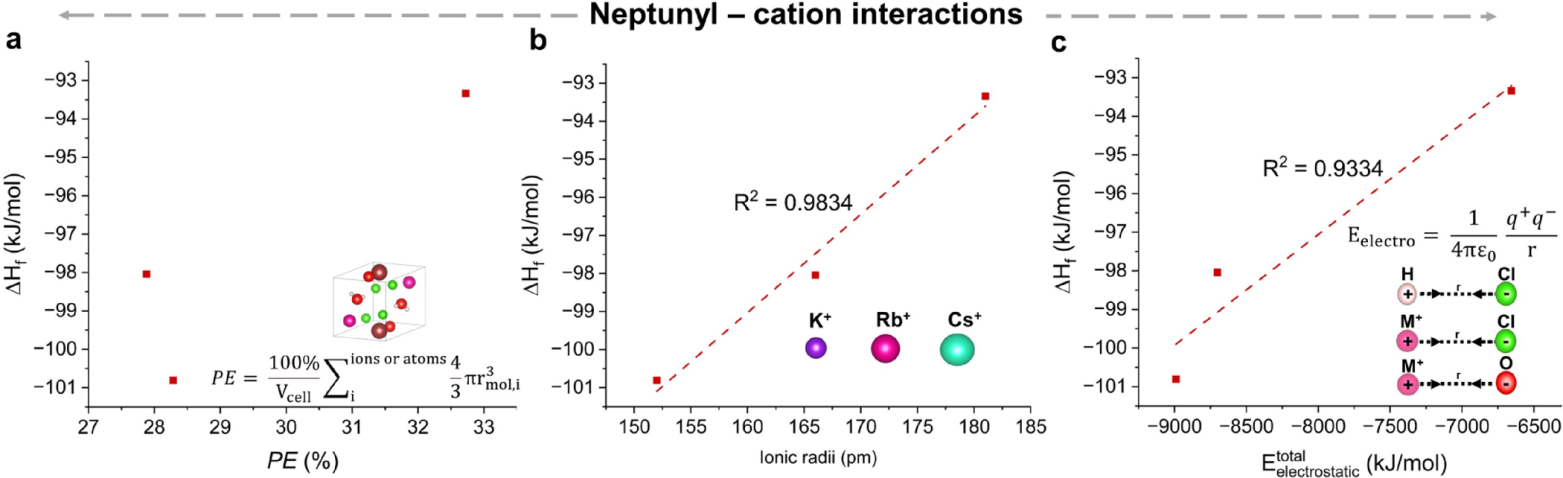
Correlation plots for neptunyl–cation systems. (a) Plot of Δ*H*_f_ vs PE; the equation used to calculate the PE is highlighted in the image. (b) Plot of Δ*H*_f_ vs *r*_ionic_. (C) Plot of Δ*H*_f_ vs *E*_electrostatic_^total^; here, electrostatic interactions are seen between M^+^ (M = K, Rb, or Cs) and Cl_eq_, O_yl_, and O_water_. Hydrogen bonding was observed between the water and Cl_eq_. The full definitions of the embedded equations in Figure 5a,c are given in eqs 6 and 10, respectively.

The PE was evaluated as a descriptor for Δ*H*_f_ for the Np(VI) tetrachloro compounds containing alkali cations. Plotting Δ*H*_f_ against PE reveals that a lower PE leads to more exothermic Δ*H*_f_ values (Figure 5a); given the small number of data points, it is not clear that there is a relationship to Δ*H*_f_. A similar observation is observed in the related U(VI) compounds (Supporting Information, Section 5, Figure S3).

#### Ionic Radii of the Alkali Metal Cation (*r*_ionic_)

Crystalline phases of ionic salts (like KCl) are primarily held together by electrostatic interactions; thus, the smaller size of a cation may stabilize the overall coordination compound due to a higher charge density.^51−55^ The radius of the ionic cation, *r*_ionic_, was plotted against Δ*H*_f_ (Figure 5b), and a linear trend (*R*^2^ = 0.9834) was observed between *r*_ionic_ and Δ*H*_f_, indicating a direct correlation between the two descriptors. However, this does not account for structural differences in the overall materials.

#### Electrostatic Attraction Energy (*E*_electrostatic_^total^)

The *E*_electrostatic_^total^ was calculated by eqs 10 and 11 where the symbols hold the meaning as defined in Section 3.3.1. The calculated *E*_electrostatic_^total^ values are provided in Table 4.
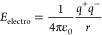
10
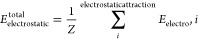
11

**Table 4:** Calculated *E*_electrostatic_^total^ of Neptunyl(VI)–Cation and Uranyl(VI)–Cation Systems

**compound**	***E***_electrostatic_^**total**^(kJ/mol)	***E***_electrostatic_^**total**^**(of the UO**_**2**_^**2+**^**isostructural compound** (kJ/mol))^23^
Np(VI)-K	–8990.52	–8968.29
Np(VI)-Rb	–8790.03	–8684.20
Np(VI)-Cs	–6656.86	–6338.26

The *E*_electrostatic_^total^ values for Np(VI)-K and Np(VI)-Rb are similar as they are isostructural but differ drastically from that of Np(VI)-Cs. This phenomenon can be attributed to the electrostatic attraction between the water molecules in Np(VI)-K and Np(VI)-Rb and the M^+^ ion (M = K or Rb), as well as the hydrogen bonding that occurs with the equatorial chloride anion. These interactions result in an additional attraction energy of ∼2400 kJ/mol per formula unit. In summary, these findings suggest that an increase in electrostatic attraction correlates with greater stability of neptunyl(VI)–cation complexes (Figure 5c). They also demonstrate that the total electrostatic energy may be a more detailed descriptor to understand the stability of the compounds because it includes the effects from additional lattice interactions.

## 3.4 Influence of NCIs on Vibrational Properties

Vibrational features of the NpO_2_^2+^ unit have been studied experimentally and theoretically and provide rapid identification and characterization of materials.^13,15,16,18,56,57^ In *D*_*∞h*_ symmetry, NpO_2_^2+^ has four fundamental vibrational modes: the symmetric stretch (ν_1_), a doubly degenerate bending mode (ν_2_), and the antisymmetric stretch (ν_3_). Based upon this symmetry, we predict that ν_1_ is Raman active, while ν_2_ and ν_3_ are active for infrared spectroscopy. Raman spectroscopy is widely utilized as a primary tool for evaluating actinyl bond perturbations.^23,58,59^ In previous work by Forbes and co-workers, Raman spectroscopy was used to probe perturbation in ν_1_ as a result of neptunyl–hydrogen,^15,16^ neptunyl–cation,^13^ and neptunyl–neptunyl^18^ interactions. In this work, we use our understanding of the 3-D networks of NCIs in solid materials to quantitatively assess the relationship between NpO_2_^2+^ ν_1_ shifts and NCIs with neptunyl axial oxygen atoms.

All compounds were analyzed with solid-state Raman (fitted Raman spectra are provided in the Supporting Information, Section 7.1, Figures S5–S12), and DFT phonon analysis was utilized to facilitate the identification of ν_1_ observed in the spectral range of 700–900 cm^–1^. The method employed for the computational assessment of vibrational signals was based on the approach utilized by Spano et al.^60^ and our prior work (calculated phonon spectra with a range of 600–1200 cm^–1^ are provided in the Supporting Information, Section 7.2, Tables S33–S45).^24^ When there are more than one neptunyl center in the unit cell (*Z* > 1), our calculations demonstrate the presence of several Np=O stretching bands emerging from the in-phase and out-of-phase vibration of neptunyl centers. However, while we could see these multiple bands experimentally, it was difficult to discern whether they were the product of coupling or an impurity, so we chose the neptunyl band with the highest intensity for our analysis.^61^ To assess the impact of NCIs on ν_1_, we calculated the sum of bond orders (BO_sum_^″yl″^) of all NCIs of a single axial oxygen using eq 12 where the BO_*i*_^″yl″^ is the bond order of NCIs through the axial oxygen calculated by the DDEC6 method.^34−37^ Strong hydrogen bonding interactions have previously been found to have BOs ranging from 0.07 to 0.130;^36^ therefore, this value will be used as a guide when comparing NCIs in neptunyl complexes.
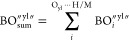
12

### Neptunyl–Hydrogen Compounds

**Figure 6:**
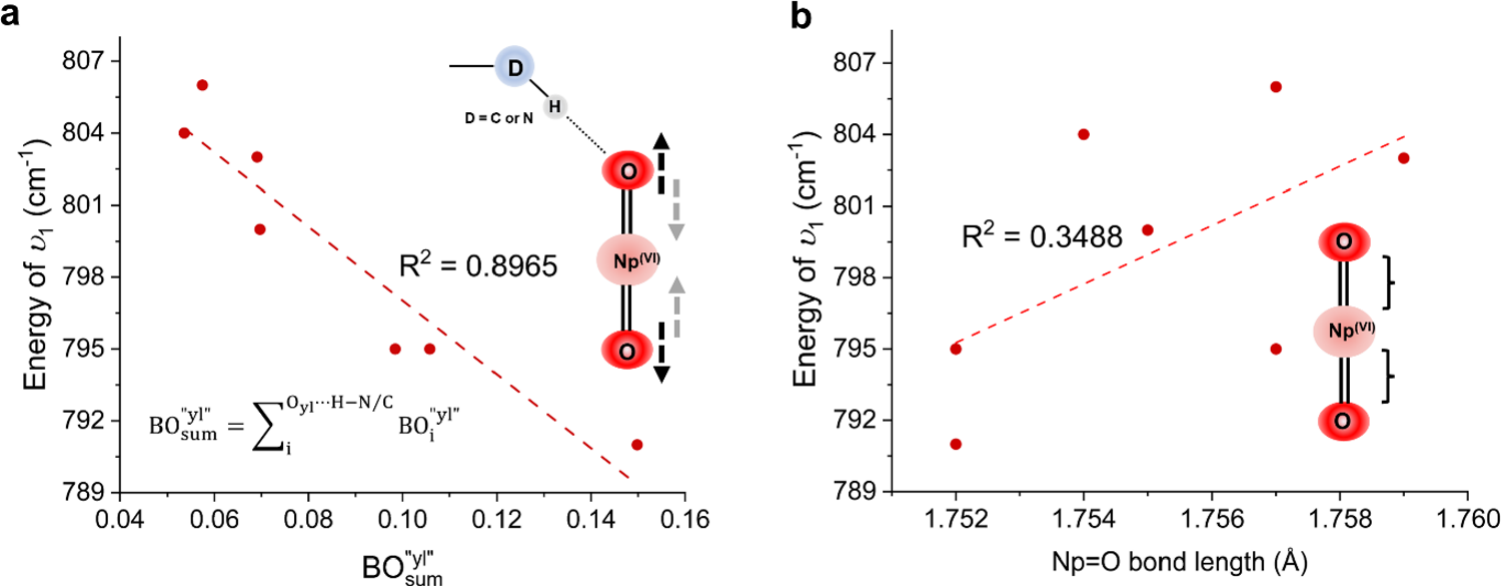
(a) Plot of ν_1_ energy (cm^–1^) vs BO_sum_^″yl″^. The BO_sum_^″yl″^ was calculated by summing the BOs of all of the hydrogen bonding interactions through the axial oxygen. (b) Plot of ν_1_ energy (cm^–1^) vs Np=O bond length (Å).

A red-shift of ν_1_ compared to the Np(VI) pentaqua complex^57^ can be observed and related to both types of hydrogen bonding (N–H···O_yl_ and C–H···O_yl_) across all the compounds containing neptunyl–hydrogen interactions. The strongest Np=O bond perturbation due to hydrogen bonding (BO_sum_^″yl″^= 0.150) is observed in Np(VI)-Pipz, where ν_1_ is red-shifted to 791 cm^–1^ (ν_1_ of [NpO_2_Cl_4_]^2–^ typically occurred at 795–830 cm^–1^).^15,62,63^ Likewise, strong hydrogen bonding to the neptunyl oxo in Np(VI)-Bipy (BO_sum_^″yl″^ = 0.106) and Np(VI)-Pyr (BO_sum_^″yl″^= 0.098) is observed. This strong hydrogen bonding can be related to the position of the ν_1_ symmetric stretch that is centered at 795 cm^–1^ (Table 5). To better visualize the impact of hydrogen bonding on the ν_1_ symmetric stretch, we plotted BO_sum_^″yl″^ against ν_1_ and fitted it with a linear model (Figure 6a). Our results quantitatively show a strong correlation between the degree of hydrogen bonding and the relative shift for the neptunyl ν_1_ symmetric stretch (*R*^2^ = 0.8965). Comparing the BO_sum_^″yl″^ of neptunyl tetrachloro complexes with isostructural uranyl complexes did not show a systemic increase in BO_sum_^″yl″^. This observation aligns well with previous work by Surbella et al., where a negligible reduction of 1.30% in electrostatic potential on axial oxygen is noted going from [UO_2_Cl_4_]^2–^ to [NpO_2_Cl_4_]^2–^.^10^ This similarity in hydrogen bonding capabilities may be the reason why NpO_2_^2+^ and UO_2_^2+^ share a similar crystallographic structure. Comparing Np(VI) and Np(V) systems, we notice a significant difference in the BO_sum_^″yl″^ between Np(VI) and Np(V) tetrachloro compounds (Np(V)-Morph, 0.260, and Np(V)-Pipz, 0.294). However, we found no association between the crystallographic Np=O bond length and the ν_1_ value (Figure 6b). This finding is in accordance with our earlier work and shows that computational treatment of the 3-D NCI network is required to effectively probe the Np=O bond perturbation due to NCIs.^23^

**Table 5:** Experimental Symmetrical Stretch, Reported in cm^–1^, Average Crystallographic Np=O Bond Length, Reported in Å, and Values of BO_sum_^″yl″^, Calculated Using eq 12, of Np(VI) Compounds

**type of interaction**	**compound**	**experimental υ**_**1**_**(cm**^**–1**^**)**	**average Np=O bond length (**Å**)**	**BO**_**sum**_^″**yl**″^	**BO**_**sum**_^″**yl**″^**of UO**_**2**_^**2+**^**isomorph**(23)
neptunyl–hydrogen	Np(V)-Morph	758^15^	1.833^15^	0.260	
	Np(V)-Pipz	734^15^	1.843^15^	0.294	
	Np(VI)-Bipy	795	1.757	0.106	0.108
	Np(VI)-Gua	804	1.754	0.054	0.028
	Np(VI)-Morph	803	1.759	0.069	0.031
	Np(VI)-Apyr	800	1.755	0.070	0.072
	Np(VI)-Imi	806	1.757	0.057	0.074
	Np(VI)-Pipz	791	1.769	0.150	0.160
	Np(VI)-Pyr	795^15^	1.752^15^	0.098	
neptunyl–cation	Np(V)-Cs		1.814^39^	0.375	
	Np(VI)-K	804	1.760	0.127	0.148
	Np(VI)-Rb	803	1.762	0.130	0.135
	Np(VI)-Cs	802^39^	1.775	0.143	0.127

### Neptunyl–Cation Compounds

Axial oxygen engagement in neptunyl–cation interactions can be seen occurring to varying degrees across all three compounds. The highest-level engagement is seen in Np(VI)-Cs (BO_sum_^″yl″^ = 0.144). The effect that cation interactions have on ν_1_ is too small to reliably assess spectroscopically because the observed variation in ν_1_ (802–804 cm^–1^) falls within repeatability limits of the Raman spectrometer that was used for this investigation (±2 cm^–1^). There is a drastic increase of BO_sum_^″yl″^ when comparing Np(VI)-Cs (0.143) with Np(V)-Cs (0.375), which indicates that Np(V) has significantly higher ability to engage in neptunyl–cation interactions.

## 4 Conclusions

In this study, we utilized the [NpO_2_Cl_4_]^2–^ system to evaluate the influence of NCIs on the thermochemical and vibrational properties of neptunyl(VI) solid-state compounds. By combination of [NpO_2_Cl_4_]^2–^ with organic or alkaline metal cations, seven compounds with neptunyl–hydrogen interactions and three compounds with neptunyl–cation interactions were synthesized and structurally characterized. The DFT+ T methodology was employed to compute the Δ*H*_f_ values of all neptunyl compounds. The validity of the theoretical approach was established by comparing to the experimental Δ*H*_f_ values obtained through isothermal acid calorimetry with accuracy reaching ±8%. We evaluated three descriptors of Δ*H*_f_ for systems containing neptunyl–hydrogen or neptunyl–cation interactions. For hydrogen-bonded systems, our results showed a strong correlation between Δ*H*_f_ and hydrogen bonding, where increasing the total hydrogen bonding strength within the system resulted in greater stability. For compounds containing neptunyl–cation interactions, the Δ*H*_f_ exhibited a significant correlation with total electrostatic attractions, with more electrostatic attraction energy resulting in a more stable neptunyl solid-state complex. Finally, we assessed the dependence of the neptunyl symmetric stretch (ν_1_) position on NCIs. We demonstrated quantitatively that more hydrogen bonding results in greater red-shifting of the ν_1_ band. A similar observation was seen for neptunyl–cation interactions as well.

Our results indicated that the strength of hydrogen bonding and electrostatic attraction did not vary considerably between [Np(VI)O_2_Cl_4_]^2–^ and [U(VI)O_2_Cl_4_]^2–^, but it did change significantly when transitioning to [Np(V)O_2_Cl_4_]^3–^. The *E*_H_^total^ and *E*_electrostatic_^total^ values of the neptunyl(VI)–hydrogen and neptunyl(VI)–cation phases are consistent with those of isostructural U(VI) phases, indicating that they have comparable electrostatic attraction. The BO_sum_^″yl″^ values also remain comparable between Np(VI) and U(VI) phases, showing similar degrees of axial oxygen engagement in NCIs. However, when Np(V) compounds were compared to related Np(VI) materials, a significant increase in axial oxo engagement was observed in the BO_sum_^″yl″^ values.

Overall, our findings demonstrate the importance of NCIs in the thermochemistry and vibrational properties of neptunyl solid-state complexes. In addition, we have shown that it is important to account for the whole NCI network to predict the stability and vibrational signatures of new neptunyl phases. This work can be expanded by exploring the impact of the equatorial ligands on the NCI network for actinyl phases and comparing related plutonyl systems to evaluate systematic trends for the transactinide elements.

## Supporting Information Available

Synthesis of neptunyl solid-state complexes; crystallographic information; powder X-ray diffraction; computational details; calorimetric data and thermocycles; list of NCIs; Raman spectra; DFT calculated phonon modes (PDF)
