# Supplementary material for: Three-Dimensional Noncovalent Interaction Network within [NpO2Cl4]2– Coordination Compounds: Influence on Thermochemical and Vibrational Properties
Source: Inorg Chem. 2023 Oct 10;62(42):17265–75. doi: 10.1021/acs.inorgchem.3c02502 (PMC10598792; doi:10.1021/acs.inorgchem.3c02502)
Supplement: Supplementary file 1 — ic3c02502_si_001.pdf [file ic3c02502_si_001.pdf]

Three-Dimensional Non-Covalent Interaction Network within $[NpO_2Cl_4]^{2-}$ Coordination Compounds: Influence on Thermochemical and Vibrational Properties

Authors: Harindu Rajapaksha[a], Grant C. Benthin[a], Dmytro V. Kravchuk[a], Haley Lightfoot[a], Sara E. Mason*[a,b,], Tori Z. Forbes*[a].

[a] *Department of Chemistry, University of Iowa, Iowa City, IA 52242, USA*

[b] *Center for Functional Nanomaterials, Brookhaven National Laboratory, Upton, NY 11973, USA*

*Corresponding Authors: Tori Z. Forbes Email: tori-forbes@uiowa.edu, Sara E. Mason Email: smason@bnl.gov

Table of Contents

1. Synthesis and *Single-Crystal X-Ray Diffraction* of crystalline materials 5

1.1 Synthesis: 5

1.2 Single-Crystal X-Ray Diffraction: 6

1.3 Crystallographic Information 7

Table S1: The crystallographic information of Np(VI)-Bipy, Np(VI)-Gua, Np(VI)-Morph, and Np(VI)-Apyr. 7

Table S2: The crystallographic information of Np(VI)-Imi, Np(VI)-Pipz, Np(VI)-K, and Np(VI)-Rb..............................8

2. Analysis of bulk purity 9

2.1 Powder X-Ray Diffraction (PXRD)..............................9

3. DFT method details 10

Table S3: The K-grids used for the DFT calculations of compounds. 10

3.1 Benchmarking of Hubbard *U* value 10

Figure S2: The variation of Np=O and Np-Cl bond lengths (a) and unit cell parameters (b) of Np(V)-pipz and the variation of Np=O and Np-Cl bond lengths (c) and unit cell parameters (d) of Np(VI)-pyr with Hubbard *U* value..............................11

Table S4: The optimized Np=O and Np-Cl bond lengths and optimized unit cell parameters and their percent errors with respect of experimental values. 12

3.2 The Preferred Magnetic orientation 13

Table S5: The electronic energies of FM and AFM configurations of systems with more than one neptunyl center in the unit cell..............................13

Table S6: The optimized magnetization Np(V/VI) ions (mu_B) of the neptunyl systems. .13

4. Formation energy analysis 14
4.1 Experimental formation enthalpy (ΔHf) determination 14
Table S7: The solvation enthalpy ($\Delta Hsol$) of Np(VI)-Pipz and Np(VI)-Pyr calculated at 25 °C and atmospheric pressure. The uncertainty $\Delta Hsol$ was calculated as 2σ of the mean. 14
Table S8: The solvation enthalpy ($\Delta Hsol$) of organic based calculated at 25 °C and atmospheric pressure. The uncertainty $\Delta Hsol$ was calculated as 2σ of the mean taken from literature.[6] 14
Table S9: The Thermocycle used for the calculation of solvation enthalpy of $NpO_{3(s)}$ in 2M HCl at at 25 °C and atmospheric pressure. 14
Table S10: The thermocycles for used the calculation of formation enthalpies of **Np(VI)-Pipz**. Solvation energies of **Np(VI)-Pipz** ($\Delta H5$) was found by isothermal acid calorimetry. $\Delta H2$, $\Delta H3$ and $\Delta H4$ were obtained from literature. 14
Table S11: The thermocycles used for the calculation of formation enthalpies of **Np(VI)-Pyr**. Solvation energies of **Np(VI)-Pyr** ($\Delta H5$) was found by isothermal acid calorimetry. $\Delta H2$, $\Delta H3$ and $\Delta H4$ were obtained from literature. 15
4.2 Computational formation enthalpy (ΔHf) determination: DFT+ Thermodynamics 16
Table S12: The thermocycles used for the calculation of formation enthalpies of **Np(VI)-Bipy**, **Np(VI)-Gua**, **Np(VI)-Morph**, **Np(VI)-Apyr**, **Np(VI)-Imi**, **Np(VI)-Pipz** and **Np(VI)-Pyr** 16
Table S13: The thermocycles used for the calculation of formation enthalpies of **Np(VI)-K** and **Np(VI)-Rb**. Here M = K or Rb. 16
Table S14: The thermocycles used for the calculation of formation enthalpies of **Np(VI)-Cs.** 17
Table S15: The thermocycles used for the calculation of formation enthalpies of **Np(V)-Cs**. 17
Table S16: The thermocycles used for the calculation of formation enthalpies of **Np(V)-Morph**. 18
Table S17: The thermocycles used for the calculation of formation enthalpies of **Np(V)-Morph**. 18
5. Descriptors of Δ**Hf** 19
Figure S3: The plot of ΔHf vs *PE* of uranyl-cation systems 19
6. Analysis of Non-Covalent Interaction 20
Figure S4: The Non-Covalent Interaction (NCI) network of tested Np(V) compounds. 23
6.1 Bond order calculation 23
Table S18: Full list of hydrogen bonding interactions in the unit cell of Np(V)-Morph. 24
Table S19: Full list of hydrogen bonding interactions in the unit cell of Np(V)-Pipz. 26
Table S20: Full list of cation interactions in the unit cell of Np(V)-Cs. 27
Table S21: Full list of hydrogen bonding interactions in the unit cell of Np(VI)-Bipy. 29

Table S22: Full list of hydrogen bonding interactions in the unit cell of Np(VI)-Gua.........30
Table S23: Full list of hydrogen bonding interactions in the unit cell of Np(VI)-Morph. ...31
Table S24: Full list of hydrogen bonding interactions in the unit cell of Np(VI)-Apyr. 32
Table S25: Full list of hydrogen bonding interactions in the unit cell of Np(VI)-Imi..........32
Table S26: Full list of hydrogen bonding interactions in the unit cell of Np(VI)-Piz. 33
Table S27: Full list of hydrogen bonding interactions in the unit cell of Np(VI)-Pyr..........34
Table S28: Full list of cation interactions in the unit cell of Np(VI)-K...............................35
Table S29: Full list of hydrogen interactions in the unit cell of Np(VI)-K 35
Table S30: Full list of cation interactions in the unit cell of Np(VI)-Rb.............................36
Table S31: Full list of hydrogen interactions in the unit cell of Np(VI)-Rb........................36
Table S32 Full list of cation interactions in the unit cell of Np(VI)-Cs...............................37
7. Vibrational analysis 38
7.1 Raman spectroscopic analysis of neptunyl complexes. 38
Figure S5: The fitted Raman spectrum of **Np(VI)-Bipy** in the spectral window 600-1200 cm^{-1}. 38
Figure S6: The fitted Raman spectrum of **Np(VI)-Gua** in the spectral window 600-1100 cm^{-1} 39
Figure S7: The fitted Raman spectrum of **Np(VI)-Morph** in the spectral window 600-1200 cm^{-1} 40
Figure S8: The fitted Raman spectrum of **Np(VI)-Apyr** in the spectral window 600-1200 cm^{-1}. 41
Figure S9: The fitted Raman spectrum of **Np(VI)-Imi** in the spectral window 600-1200 cm^{-1}. 42
Figure S10: The fitted Raman spectrum of **Np(VI)-Pipz** in the spectral window 600-1200 cm^{-1} 43
Figure S11: The fitted Raman spectrum of **Np(VI)-K** in the spectral window 600-1200 cm^{-1}. 44
Figure S12: The fitted Raman spectrum of **Np(VI)-Rb** in the spectral window 600-1200 cm^{-1}. 45
7.2 Computation vibration analysis: Calculated phonon frequencies, neptunyl displacement amplitudes, and oxo displacement phase angle 46
Table S33: **Np(V)-Morph** phonon mode analysis in the spectral window of 600-1200 cm^{-1}. Red modes correspond to neptunyl symmetric (ν_1) and blue modes correspond to neptunyl asymmetric (ν_3) stretches. 47
Table S34: **Np(V)-Pipz** phonon mode analysis in the spectral window of 600-1200 cm^{-1} Red modes correspond to neptunyl symmetric (ν1) and blue modes correspond to neptunyl asymmetric (ν3) stretches. 50

Table S35: **Np(V)-Cs** phonon mode analysis in the spectral window of 600-1200 cm^{-1}. Red modes correspond to neptunyl symmetric (ν1) and blue modes correspond to neptunyl asymmetric (ν3) stretches. 51

Table S36: **Np(VI)-Bipy** phonon mode analysis in the spectral window of 600-1200 cm^{-1}. Red modes correspond to neptunyl symmetric (ν_1) and blue modes correspond to neptunyl asymmetric (ν_3) stretches. 51

Table S37: **Np(VI)-Gua** phonon mode analysis in the spectral window of 600-1200 cm^{-1}. Red modes correspond to uranyl symmetric (ν_1) and asymmetric (ν_3) stretches. 54

Table S38: Np(VI)-Morph phonon mode analysis in the spectral window of 600-1200 cm-1. Red modes correspond to neptunyl symmetric (ν_1) and blue modes correspond to neptunyl asymmetric (ν_3) stretches. 54

Table S39: **Np(VI)-Apyr** phonon mode analysis in the spectral window of 600-1200 cm^{-1}. Red modes correspond to neptunyl symmetric (ν_1) and blue modes correspond to neptunyl asymmetric (ν_3) stretches. 55

Table S40: **Np(VI)-Imi** phonon mode analysis in the spectral window of 600-1200 cm^{-1}. Red modes correspond to neptunyl symmetric (ν_1) and blue modes correspond to neptunyl asymmetric (ν_3) stretches. 56

Table S41: **Np(VI)-Pipz** phonon mode analysis in the spectral window of 600-1200 cm^{-1}. Red modes correspond to neptunyl symmetric (ν_1) and blue modes correspond to neptunyl asymmetric (ν_3) stretches. 57

Table S42: **Np(VI)-Pyr** phonon mode analysis in the spectral window of 600-1200 cm^{-1}. Red modes correspond to neptunyl symmetric (ν_1) and blue modes correspond to neptunyl asymmetric (ν_3) stretches. 58

Table S43: **Np(VI)-K** phonon mode analysis in the spectral window of 600-1200 cm^{-1}. Red modes correspond to neptunyl symmetric (ν_1) and blue modes correspond to neptunyl asymmetric (ν_3) stretches. 59

Table S44: **Np(VI)-Rb** phonon mode analysis in the spectral window of 600-1200 cm^{-1}. Red modes correspond to neptunyl symmetric (ν_1) and blue modes correspond to neptunyl asymmetric (ν_3) stretches. 59

Table S45: **Np(VI)-Cs** phonon mode analysis in the spectral window of 600-1200 cm^{-1} Red modes correspond to neptunyl symmetric (ν_1) and blue modes correspond to neptunyl asymmetric (ν_3) stretches. 60

8. Reference 60

1. Synthesis and *Single-Crystal X-Ray Diffraction* of crystalline materials

1.1 Synthesis:

Caution: Neptunium-237 is a highly radioactive alpha emitter. Research with this isotope is restricted to specialized laboratories and must be handled under appropriate regulatory controls.

Np(VI)-Bipy and **Np(VI)-Pipz** were synthesized by mixing 100 µL of 0.27 M Np(VI) stock in 2 M HCl with 100 µL of 0.25 M 2',2-Bipyridne (Alfa Aesar, ≥99%) in 2 M HCl and 100 µL of 0.25 M of Piperazine (Aldrich, ≥99%) respectively. The solution was slowly evaporated, and yellow-orange crystals were formed. Given the radioactivity of the materials, quantitative yield was not calculated.

Np(VI)-Gua, Np(VI)-Morph, Np(VI)-Apyr, and **Np(VI)-Imi** were synthesized by mixing 100 µL of 0.27 M Np(VI) stock in 2 M HCl with 100 µL of 0.50 M Guanidium chloride (Aldrich, ≥99%) in 2 M HCl ,100 µL of 0.50 M of morpholine (Thermo Fisher, , ≥99%) in 2 M HCl, 100 µL of 0.50 M 4-Aminopyrdine (Acros organics, ≥98%), in 2 M HCl, and 100 µL of 0.50 M Imidazole (Acros organics) in 2 M HCl respectively. The solution was slowly evaporated, and yellow-orange crystals were formed. Given the radioactivity of the materials, quantitative yield was not calculated.

Np(VI)-Rb 100 µL of 0.27 M Np(VI) stock in 2 M HCl with 100 µL of 0.5 M of RbCl (Aldrich, ≥99%) respectively. The solution was slowly evaporated, and yellow crystals were formed. Given the radioactivity of the materials, quantitative yield was not calculated.

Np(VI)-K was synthesized by mixing 100 µL of 0.27 M Np(VI) stock in 2 M HCl with 100 µL of 0.5 M KCl (Aldrich, ≥99%) in 2 M HCl. The solution was rapidly evaporated in a desiccator under vacuum and yellow crystals were formed within 15 minutes. Attempts to slow evaporate the solutions resulted in the formation of yellow glass phase.

Synthesis of Np(VI)-Pipz and Np(VI)-Pyr for calorimetry: After the initial optimization of the synthesis, two phases were chosen based upon their yield and purity for larger scale synthesis to use in calorimetric measurements. The large scale synthesis mixed 750 μL of 0.27 M Np(VI) in 2 M HCl with 750 μL of 0.25 M of Piperazine (Aldrich, ≥99%) in 2M HCl or 750 μL of 0.50 M of pyridine (Aldrich, ≥99%) in 2M HCl and the solution was slowly evaporated. To ensure purity, crystals were isolated before the mother liquor evaporated to dryness and isolated crystals were dried in a desiccator.

1.2 Single-Crystal X-Ray Diffraction:

Single crystals were isolated from the mother liquor. The single crystals were mounted on a MiTeGen MicroMount and reflections were collected using 0.5° □ scans on Brucker® D8 Quest single X-ray diffractometer ($\lambda_{MoK\alpha}$= 0.71073 Å) with PHOTON detector and Oxford® cryo-system. Absorption corrections were performed using the SADABS program. APEX3 intrinsic phasing 1 was used for the solving of structures and the structure refinements were done SHELXL with OLEX2 software suite. The hydrogens of the aromatic rings, non-aromatic rings, amine groups and water was modeled with AFIX 43, AFIX 23, AFIX 93 and AFIX 7 respectively

1.3 Crystallographic Information

Table S1: The crystallographic information of Np(VI)-Bipy, Np(VI)-Gua, Np(VI)-Morph, and Np(VI)-Apyr.

**Compound**	**Np(VI)-Bipy**	**Np(VI)-Gua**	**Np(VI)-Morph**	**Np(VI)-Apyr**
Empirical Formula	$(C_{10}H_8N_2)[NpO_2Cl_4]$	$(CH_6N_3)_2[NpO_2Cl_4]$	$(C_4H_{10}NO)_2[NpO_2Cl_4]$	$(C_5H_7N_2)_2[NpO_2Cl_4]$
Crystal color and habit	yellow prismatic	yellow prismatic	yellow prismatic	yellow prismatic
Formula weight (g)	569.00	530.98	585.04	601.05
Crystal system	Monoclinic	Monoclinic	Monoclinic	Triclinic
a (Å)	8.9168(7)	6.8917(2)	7.4233(3)	6.8775(3)
b (Å)	11.8517(7)	10.1376(4)	8.0271(4)	6.9819(3
c (Å)	14.4934(12)	9.7740(3)	15.0490(7)	9.3995(4)
α (°)	90	90	90	89.540(2)
β (°)	94.536(4)	105.1330(10)	104.230(2)	72.317(2)
γ (°)	90	90	90	81.504(2)
Unit cell volume ($Å^3$)	1526.9(2)	659.18(4)	869.22(7)	424.98(3)
Density, ρ (g/cm^3)	2.475	2.675	2.235	2.349
Space group	$P2_1/n$	$P2_1/n$	$P2_1/n$	*P*-1
Z	4	2	2	1
μ (mm^{-1})	7.502	8.685	6.600	6.748
F(000)	1044	486	546	279
Θ range (°)	5.188 to 52.742	5.898 to 50.692	5.586 to 50.696	5.904 to 50.698
Limiting indices	$-7 \leq h \leq 11$, $-13 \leq k \leq 12$, $-17 \leq l \leq 12$	$-8 \leq h \leq 8$, $-12 \leq k \leq 12$, $-11 \leq l \leq 11$	$-8 \leq h \leq 8$, $-9 \leq k \leq 9$, $-18 \leq l \leq 17$	$-8 \leq h \leq 8$, $-8 \leq k \leq 8$, $-11 \leq l \leq 11$
Ref. collected/unique	5667	25637	17490	15056
R_{int}	0.0265	0.0299	0.0491	0.0205
Data/restraints/parameters	2368/0/184	1216/0/71	1588/0/89	1557/0/98
GOF on F^2	1.036	1.133	1.069	1.081
R_1 ([I>2σ(I)])	0.0258	0.0092	0.0333	0.0075
wR_2 ([I>2σ(I)])	0.0577	0.0209	0.0655	0.0197
R_1 (all data)	0.0457	0.0108	0.0450	0.0075
wR_2 (all data)	0.0674	0.0214	0.0713	0.0197
Largest diff. peak/hole (e $Å^{-3}$)	0.81/-0.94	0.41/-0.30	1.27/-0.93	0.29/-0.52
CCDC deposition number	2260671	2260672	2260674	2260670

Table S2: The crystallographic information of Np(VI)-Imi, Np(VI)-Pipz, Np(VI)-K, and Np(VI)-Rb.

**Compound**	**Np(VI)-Imi**	**Np(VI)-Pipz**	**Np(VI)-K**	**Np(VI)-Rb**
Empirical Formula	$(C_3H_5N_2)_2[NpO_2Cl_4]$	$(C_4H_{12}N_2)[NpO_2Cl_4]$	$K_2[NpO_2Cl_4](H_2O)_2$	$Rb_2[NpO_2Cl_4](H_2O)_2$
Crystal color and habit	yellow prismatic	yellow prismatic	yellow prismatic	yellow prismatic
Formula weight (g)	548.98	498.96	525.03	599.76
Crystal system	Triclinic	Triclinic	Triclinic	Triclinic
a (Å)	7.5964(3)	6.5914(3)	6.6134(3)	6.7094(3)
b (Å)	9.6152(4)	6.6658(3)	6.7053(3)	6.8694(3)
c (Å)	10.7958(5)	7.6683(4)	7.2473(3)	7.4462(3)
α (°)	79.645(2)	84.955(2)	92.8350(10)	92.469(2)
β (°)	71.365(2)	89.210(2)	101.6420(10)	101.557(2)
γ (°)	73.950(2)	61.637(2)	118.6120(10)	118.536(2)
Unit cell volume ($Å^3$)	714.44(5)	295.18(2)	272.31(2)	291.52(2)
Density, ρ (g/cm^3)	2.552	2.807	3.202	3.416
Space group	*P*-1	*P*-1	*P*-1	*P*-1
Z	2	1	1	1
μ (mm^{-1})	8.015	9.679	11.253	18.081
F(000)	502	227	235	139
Θ range (°)	4.43 to 50.684	5.336 to 50.678	5.824 to 50.694	5.658 to 50.698
Limiting indices	$-9 \leq h \leq 9$, $-11 \leq k \leq 11$, $-13 \leq l \leq 13$	$-7 \leq h \leq 7$, $-8 \leq k \leq 8$, $-9 \leq l \leq 9$	$-7 \leq h \leq 7$, $-8 \leq k \leq 8$, $-8 \leq l \leq 8$	$-8 \leq h \leq 7$, $-8 \leq k \leq 8$, $0 \leq l \leq 8$
Ref. collected/unique	27720	11546	10663	19512
R_{int}	0.0324	0.0354	0.0263	0.0507
Data/restraints/parameters	2618/0/170	1073/0/61	992/0/62	1062/0/55
GOF on F^2	1.140	1.155	1.383	0.822
R_1 ([I>2σ(I)])	0.0128	0.0099	0.0135	0.0232
wR_2 ([I>2σ(I)])	0.0243	0.0237	0.0396	0.0849
R_1 (all data)	0.0173	0.0099	0.0135	0.024
wR_2 (all data)	0.0258	0.0237	0.0396	0.0863
Largest diff. peak/hole (e $Å^{-3}$)	0.82/-0.38	0.27/-0.64	0.34/-1.45	1.07/-1.33
CCDC deposition number	2260673	2260675	2260669	2260490

2. Analysis of bulk purity

2.1 Powder X-Ray Diffraction (PXRD)

Np(VI)-Pipz and Np(VI)-Pyr were characterized by PXRD before calorimetric measurements to ensure purity of the samples. Crystallites were randomly selected and glued to a glass fiber with 5-minute epoxy. The Glass fiber was mounted to Rigaku® XtaLAB Synergy-I diffractometer with Cu radiation source ($\lambda_{CuK\alpha}$= 1.5406 Å). The powder pattern for the crystalized were generated through Gandolfi powders method with 2.5 hr scan with detector 150 mm. The generated background subtracted powder pattern overlapped with calculated pattern to analyze the composition of the crystallites. Experimental and calculated patterns show good agreement proving the purity of **Np(VI)-Pipz** and **Np(VI)-Pyr** phase used for the calorimetry.

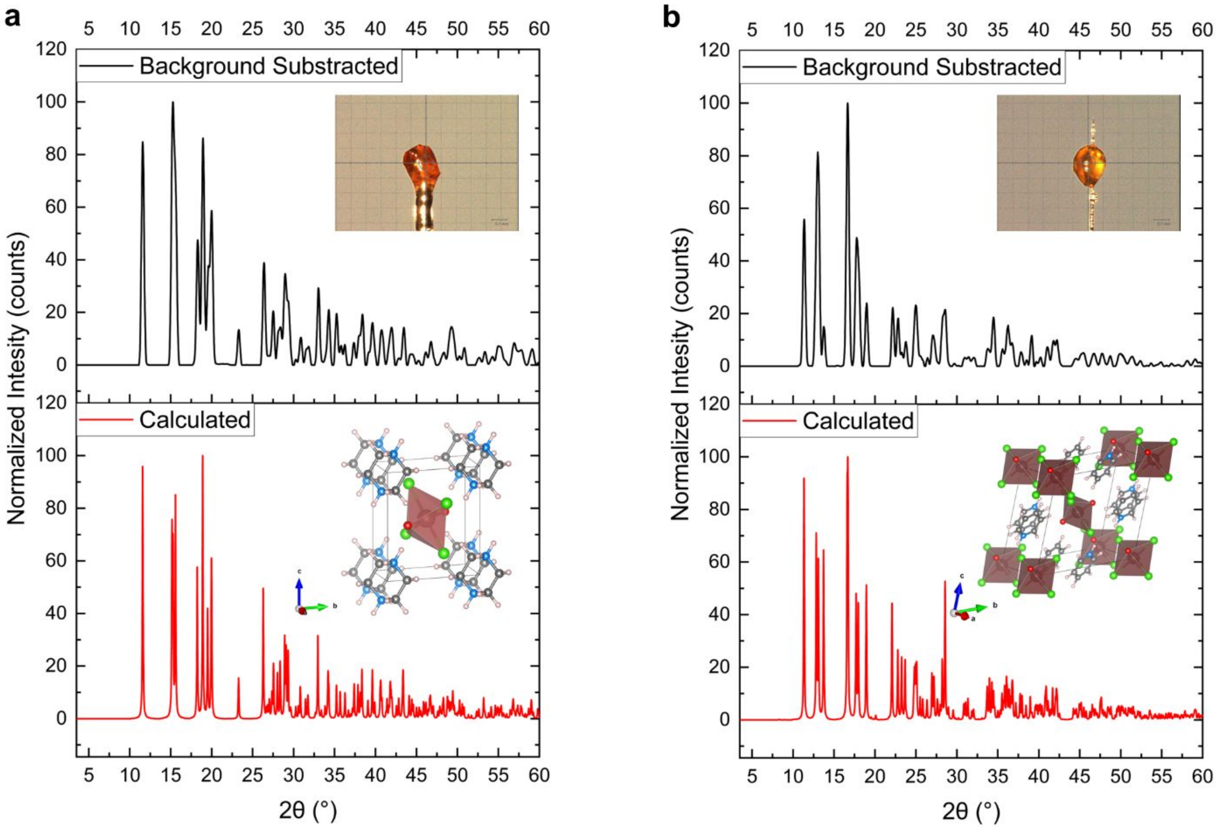

Figure S1: Experimental and calculated powder patterns of (a) **Np(VI)-Pipz** and (b) **Np(VI)-pyr** phases respectively.

3. DFT method details

Table S3: The K-grids used for the DFT calculations of compounds.

Compound	K-grid	Compound	K-grid
Np(V)-Morph	5 x 3 x 3	Np(VI)-Imi	6 x 4 x 4
Np(V)-Pipz	6 x 4 x 3	Np(VI)-Pipz	7 x 7 x 5
Np(V)-Cs	3 x 6 x 4	Np(VI)-Pyr	6 x 4 x 4
Np(VI)-Bipy	5 x 3 x 3	Np(VI)-K	7 x 7 x 6
Np(VI)-Gua	6 x 4 x 4	Np(VI)-Rb	7 x 7 x 6
Np(VI)-Morph	6 x 5 x 3	Np(VI)-Cs	3 x 5 x 7
Np(VI)-Apyr	6 x 6 x 4		

3.1 Benchmarking of Hubbard *U* value

The accuracy of Hubbard *U* corrections[1-3] is typically benchmarked by comparing the experimental bandgap with calculated bandgap. Due to the unavailability of experimental bandgap data for Np(V/VI) hybrid materials, we have used unit cell parameters and bonding within the Neptunyl unit as the metric of assessing the accuracy of Hubbard *U* correction. A greater attention was paid to Np≡O bond as it exhibits the greatest covalency within the inner coordination sphere of $[NpO_2Cl_4]^{-/2-}$ unit.[4, 5]

A Hubbard *U* correction was applied to the neptunium *f* states of Np(V)-pipz and Np(VI)-pyr systems following the approach of Dudarev et al. by varying U-J value from 2.5 to 5.0 with increments of 0.25. Figure S2 shows the variation of percent error of $[NpO_2Cl_4]^{-/2-}$ bond distances and unit cell parameters. Hubbard *U* value of 3.75 was chosen for both Np(V) and Np(VI) compounds as it results in the least overalls errors respect both Np=O and Np-Cl bonding and resulted in percent errors of < 3.5% for unit cell parameters. With Hubbard *U* value of 3.75 all the neptunyl systems had Np=O and Np-Cl bond lengths errors < 1% and <1.3% respectively (Table S4). The optimized unit cell parameters were <3.5% in all the cases.

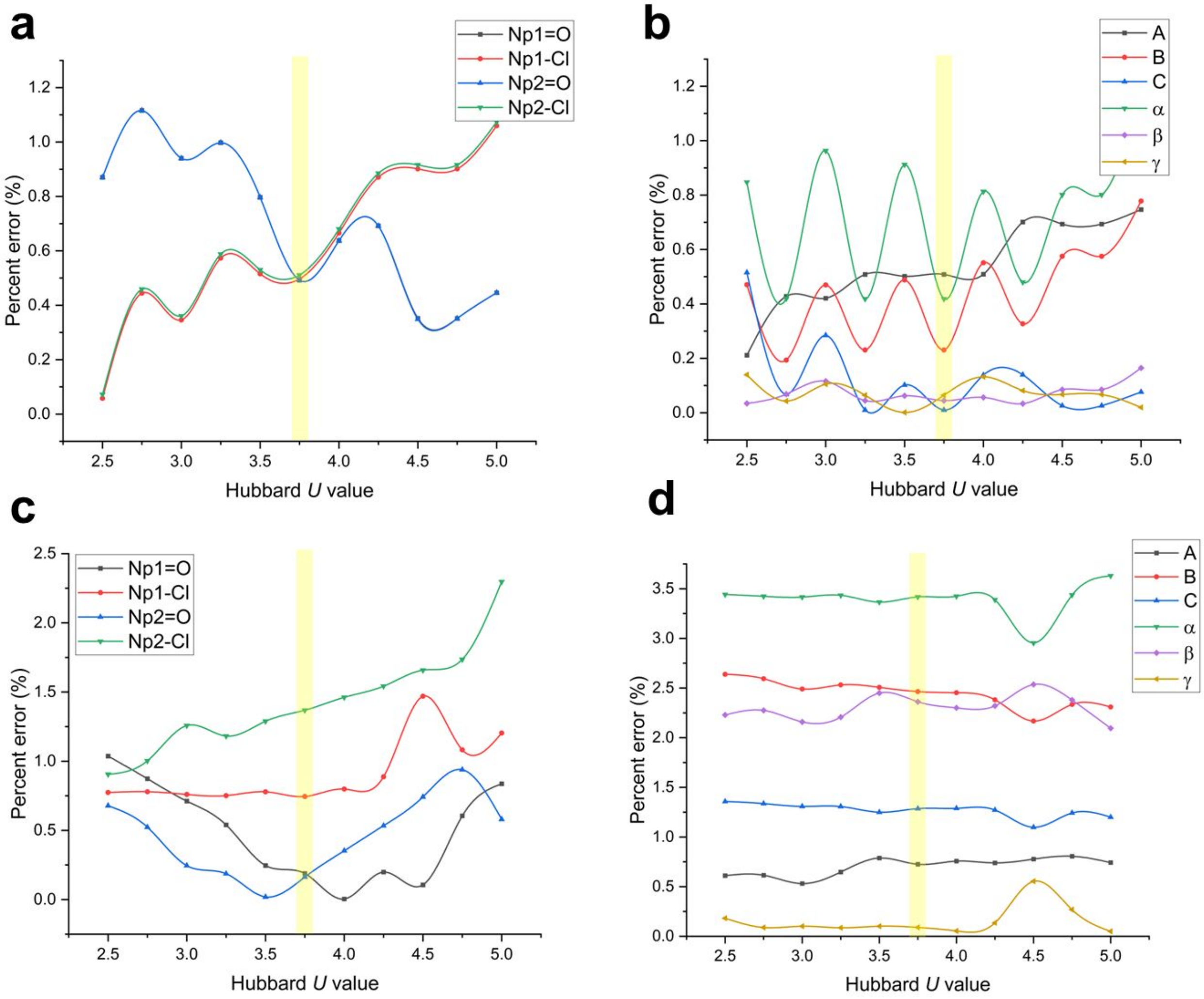

Figure S2: The variation of Np=O and Np-Cl bond lengths (a) and unit cell parameters (b) of Np(V)-pipz and the variation of Np=O and Np-Cl bond lengths (c) and unit cell parameters (d) of Np(VI)-pyr with Hubbard *U* value.

Table S4: The optimized Np=O and Np-Cl bond lengths and optimized unit cell parameters and their percent errors with respect of experimental values.

system		Np=O (Å)	Np-Cl (Å)	A (Å)	B (Å)	C (Å)	α (⁰)	β (⁰)	γ (⁰)
Np(V)-Morph	Opt Value	1.840	2.778	7.321	20.469	14.294	90.000	96.631	90.000
	|% error|	0.319	0.672	0.505	0.160	0.845	0.000	0.061	0.000
Np(V)-Pipz	Opt Value	1.852	2.752	7.381	10.498	12.295	75.770	89.377	71.734
	|% error|	0.606	0.164	0.317	0.503	0.495	-0.946	0.071	0.085
Np(V)-Cs	Opt Value	1.829	2.778	15.605	7.309	12.770	90.000	117.569	90.000
	|% error|	0.147	0.729	0.851	0.885	0.564	0.000	0.252	0.000
Np(VI)-Bipy	Opt Value	1.756	2.682	8.890	11.948	14.369	90.000	94.415	90.000
	|% error|	0.032	0.850	0.302	0.813	0.858	0.000	0.000	0.000
Np(VI)-Gua	Opt Value	1.753	2.684	6.868	10.162	9.701	90.000	104.671	90.000
	|% error|	0.080	0.885	0.350	0.239	0.751	0.000	0.440	0.000
Np(VI)-Morph	Opt Value	1.755	2.689	7.592	7.612	15.255	90.000	106.432	90.000
	|% error|	0.922	1.248	0.225	5.172	1.368	0.000	2.112	0.000
Np(VI)-Apyr	Opt Value	1.752	2.693	6.875	6.927	9.383	89.042	72.424	81.562
	|% error|	0.200	1.164	0.043	0.788	0.175	0.557	0.148	0.072
Np(VI)-Imi	Opt Value	1.754	2.686	7.749	9.536	10.797	79.153	69.849	73.710
	|% error|	0.114	1.135	2.008	-0.826	0.013	0.618	2.125	0.324
Np(VI)-Pipz	Opt Value	1.770	2.660	6.594	6.682	7.491	83.016	86.907	62.659
	|% error|	0.086	0.981	0.038	0.242	2.307	2.283	2.582	1.659
Np(VI)-Pyr	Opt Value	1.756	2.687	7.782	10.024	11.118	75.338	70.302	75.875
	|% error|	0.237	1.126	0.855	2.556	1.382	3.416	2.234	0.215
Np(VI)-K	Opt Value	1.757	2.676	6.614	6.724	7.247	92.522	100.859	119.379
	|% error|	0.173	1.131	0.003	0.273	0.006	0.337	0.337	0.647
Np(VI)-Rb	Opt Value	1.756	2.681	11.939	7.751	5.804	90.000	99.471	90.000
	|% error|	0.310	1.264	0.941	1.243	0.621	0.000	0.085	0.000
Np(VI)-Cs	Opt Value	1.764	2.687	11.939	7.751	5.804	90.000	99.471	90.000
	|% error|	0.647	1.288	0.941	1.243	0.621	0.000	0.085	0.000

3.2 The Preferred Magnetic orientation

The preferred magnetic orientation of Np(V/VI) ions were tested when there is more than one neptunyl center in the unit cell. The ferromagnetic configuration (FM) was the most electronically favored state across all the testes systems (Table S5), thus all further calculations were performed with FM configuration. The optimized magnetization of each system is listed in Table S6.

Table S5: The electronic energies of FM and AFM configurations of systems with more than one neptunyl center in the unit cell.

System	No. of neptunyl centers in the unit cell	Electronic energy of FM configuration (eV)	Electronic energy of AFM configuration (eV)
Np(V)-Morph	4	-1240.5407	-1240.0300
Np(V)-Pipz	2	-488.6385	-488.0898
Np(V)-Cs	4	-216.1933	-215.6231
Np(VI)-Bipy	4	-748.9396	-748.9366
Np(VI)-Gua	2	-318.0940	-318.0928
Np(VI)-Morph	2	-439.1841	-439.1827
Np(VI)-Imi	2	-337.1496	-337.1442
Np(VI)-Pyr	2	-389.8027	-389.8013
Np(VI)-Cs	2	-99.6220	-99.6212

Table S6: The optimized magnetization Np(V/VI) ions (mu_B) of the neptunyl systems.

System	Optimized Magnetization (mu_B)	System	Optimized Magnetization (mu_B)
Np(V)-Morph	2.175, 2.175, 2.175, 2.175	Np(VI)-Imi	1.156, 1.148
Np(V)-Pipz	2.183, 2.183	Np(VI)-Pipz	1.167
Np(V)-Cs	2.164, 2.164, 2.164, 2.164,	Np(VI)-Pyr	1.155, 1.149
Np(VI)-Bipy	1.159, 1.159, 1.149, 1.149	Np(VI)-K	1.154
Np(VI)-Gua	1.151, 1.151	Np(VI)-Rb	1.153
Np(VI)-Morph	1.164, 1.164	Np(VI)-Cs	1.150, 1.150
Np(VI)-Apyr	1.150		

4. Formation energy analysis

4.1 Experimental formation enthalpy (ΔH_f) determination

Table S7: The solvation enthalpy (ΔH_{sol}) of Np(VI)-Pipz and Np(VI)-Pyr calculated at 25 °C and atmospheric pressure. The uncertainty ΔH_{sol} was calculated as 2σ of the mean.

Sample	**Enthalpy of solvation** (ΔH_{sol}) (kJ/mol)
**Np(VI)-Pipz**	-3.900
**Np(VI)-Pyr**	-10.081 ±1.089

Table S8: The solvation enthalpy (ΔH_{sol}) of organic based calculated at 25 °C and atmospheric pressure. The uncertainty ΔH_{sol} was calculated as 2σ of the mean taken from literature.[6]

Sample	**Enthalpy of solvation** (ΔH_{sol}) (kJ/mol)
**Piperazine**	-99.568 ±0.463
**2'2-Bipyrdine**	-9.214 ±0.174
**Imidazole**	-28.262 ±0.369
**Morpholine**	-78.217 ±0.455
**Pyridine**	-33.381 ±0.217
**4-Aminopyridine**	-31.828 ±0.287

Table S9: The Thermocycle used for the calculation of solvation enthalpy of $NpO_{3(s)}$ in 2M HCl at at 25 °C and atmospheric pressure.

ΔH₁: $Np_{(s)} + (\frac{3}{2})O_{2(g)} \rightarrow NpO_{3(s)}$ $\quad$ $\mathbf{\Delta H_1} = -1068.0 \pm 7.00$ kJ/mol[7]

ΔH₂: $H_{2(g)} \rightarrow 2H^+_{(aq)}$ $\quad$ $\mathbf{\Delta H_2} = 0$ kJ/mol[8]

ΔH₃: $Np_{(s)} + O_{2(g)} \rightarrow NpO^{2+}_{2(aq)}$ $\quad$ $\mathbf{\Delta H_3} = -860.733 \pm 4.662$ kJ/mol[7]

ΔH₄: $H_{2(g)} + (\frac{1}{2})O_{2(g)} \rightarrow H_2O_{(aq)}$ $\quad$ $\mathbf{\Delta H_4} = -285.830$ kJ/mol[8]

Overall: ($\mathbf{\Delta H_5 = \Delta H_3 + \Delta H_4 - \Delta H_1 - \Delta H_2}$)

$$NpO_{3(s)} + 2H^+_{(aq)} \rightarrow NpO^{2+}_{2(aq)} + H_2O_{(aq)} \quad \mathbf{\Delta H_5} = -78.563 \pm 8.41 \text{ kJ/mol}$$

Table S10: The thermocycles for used the calculation of formation enthalpies of **Np(VI)-Pipz**. Solvation energies of **Np(VI)-Pipz** (ΔH_5) was found by isothermal acid calorimetry. ΔH_2, ΔH_3 and ΔH_4 were obtained from literature.

ΔH₅: $NpO_{3(s)} + 2H^+_{(aq)} \rightarrow NpO^{2+}_{2(aq)} + H_2O_{(aq)}$ $\quad$ $\mathbf{\Delta H_5} = -78.563 \pm 8.41$ kJ/mol

ΔH₆: $C_4H_{10}N_{2(s)} + 2H^+_{(aq)} \rightarrow (C_4H_{12}N_2)^{2+}_{(aq)}$ $\quad$ $\mathbf{\Delta H_6} =$ -99.568 ±0.463 kJ/mol[6]

ΔH₇: $H_2O_{(aq)} \rightarrow H_2O_{(l)}$ $\quad$ $\mathbf{\Delta H_7} = 0.4$ kJ/mol[9, 10]

ΔH₈: $4HCl_{(aq,\ 2N)} \rightarrow 4HCl_{(final)}$ $\quad$ $4\mathbf{\Delta H_8} = 4 \times 2.16$ kJ/mol[9, 10]

ΔH₉: $(C_4H_{12}N_2)[NpO_2Cl_4]_{(s)} + 4H^+_{(aq)} \rightarrow (C_4H_{12}N_2)^{2+}_{(aq)} + NpO^{2+}_{2(aq)} + 4HCl_{(final)}$

$\mathbf{\Delta H_9} = \Delta H_{sol}$

Overall: ($\Delta H_5 + \Delta H_6 + \Delta H_7 + 4\Delta H_8 - \Delta H_9$)

$$NpO_{3(s)} + C_2H_{10}N_{2(s)} \ + \ 4HCl_{(l)} \ \rightarrow \ (C_4H_{12}N_2)[NpO_2Cl_4]_{(s)} + \ H_2O_{(l)}$$

Table S11: The thermocycles used for the calculation of formation enthalpies of **Np(VI)-Pyr**. Solvation energies of **Np(VI)-Pyr** (ΔH_5) was found by isothermal acid calorimetry. ΔH_2, ΔH_3 and ΔH_4 were obtained from literature.

ΔH_5: $NpO_{3(s)} + 2H^+_{(aq)} \rightarrow NpO^{2+}_{2(aq)} + H_2O_{(aq)}$ $\quad$ $\mathbf{\Delta H_5} = -78.563 \ \pm 8.41$ kJ/mol

ΔH_6: $C_5H_5N_{(s)} + H^+_{(aq)} \rightarrow (C_5H_6N)^+_{(aq)}$ $\quad$ $\mathbf{\Delta H_6}$ = -33.381 ±0.217 kJ/mol[6]

ΔH_7: $H_2O_{(aq)} \rightarrow H_2O_{(l)}$ $\quad$ $\mathbf{\Delta H_7} = 0.4$ kJ/mol[9, 10]

ΔH_8: $4HCl_{(aq,\ 2N)} \rightarrow 4HCl_{(final)}$ $\quad$ $4\mathbf{\Delta H_8} = 4 \times 2.16$ kJ/mol[9, 10]

ΔH_9: $(C_5H_6N)_2[NpO_2Cl_4]_{(s)} + 4H^+_{(aq)} \rightarrow \ 2(C_5H_6N)^+_{(aq)} + NpO^{2+}_{2(aq)} + \ 4HCl_{(final)}$

$\mathbf{\Delta H_9} = \Delta H_{sol}$

Overall: ($\Delta H_5 + 2\Delta H_6 + \Delta H_7 + 4\Delta H_8 - \Delta H_9$)

$$NpO_{3(s)} + \ 2C_5H_5N_{(s)} \ + \ 4HCl_{(l)} \ \rightarrow \ (C_5H_6N)_2[NpO_2Cl_4]_{(s)} + \ H_2O_{(l)}$$

4.2 Computational formation enthalpy (ΔH_f) determination: DFT+ Thermodynamics

The thermocycles used in the formation enthalpy calculation are as follows. The standard formation enthalpy of $HCl_{(aq,\ 298\ K\ ,\ 1\ bar)}$ (ΔH_5) and $H_2O_{(aq,\ 298\ K\ ,1\ bar)}$ (ΔH_6) together with ΔH_2, ΔH_3 and ΔH_4 were taken from NBS tables of chemical thermodynamic properties.[8]

Table S12: The thermocycles used for the calculation of formation enthalpies of **Np(VI)-Bipy**, **Np(VI)-Gua**, **Np(VI)-Morph**, **Np(VI)-Apyr**, **Np(VI)-Imi**, **Np(VI)-Pipz** and **Np(VI)-Pyr**.

ΔH₁: $NpO_{3(s,\ 0\ K)} + aC_xH_yN_{z(s,\ 0\ K)} + 2H_{2(g,\ 0\ K)} + 2Cl_{2(g,\ 0\ K)} \rightarrow ((C_xH_{y+2}N_z)_a[NpO_2Cl_4])_{(s,0K)} + H_{2(g,\ 0\ K)} + (\frac{1}{2})O_{2(g,0\ K)}$

ΔH₂: $H_{2(g,\ 0\ K)} \rightarrow H_{2(g,\ 298\ K,\ 1\ bar)}$ **ΔH_2 = 0.088 eV**

ΔH₃: $Cl_{2(g,0\ K)} \rightarrow Cl_{2(g,298\ K,\ 1\ bar)}$ **ΔH_3 = 0.095 eV**

ΔH₄: $O_{2(g,0\ K)} \rightarrow O_{2(g,298\ K,\ 1\ bar)}$ **ΔH_4 = 0.090 eV**

ΔH₅: $(\frac{1}{2})H_{2(g,298\ K,\ 1\ bar)} + (\frac{1}{2})Cl_{2(g,298\ K,\ 1\ bar)} \rightarrow HCl_{(aq,298\ K,\ 1\ bar)}$ **ΔH_5 = -1.732 eV**

ΔH₆: $H_{2(g,\ ,298\ K,\ 1\ bar)} + (\frac{1}{2})O_{2(g,298\ K,\ 1\ bar)} \rightarrow H_2O_{(l,298\ K,1\ bar)}$ **ΔH_6 = -2.962 eV**

Overall: ($\Delta H_1 - \Delta H_2 - 2\Delta H_3 + (\frac{1}{2})\Delta H_4 - 4\Delta H_5 + \Delta H_6$)

$NpO_{3(s,\ 0\ K)} + aC_xH_yN_{z(s,\ 0\ K)} + 4HCl_{(aq,298\ K,\ 1\ bar)} \rightarrow ((C_xH_{y+2}N_z)_a[NpO_2Cl_4])_{(s,0\ K)} + H_2O_{(l,\ 298\ K,\ 1\ bar)}$

Table S13: The thermocycles used for the calculation of formation enthalpies of **Np(VI)-K** and **Np(VI)-Rb**. Here M = K or Rb.

ΔH₁: $NpO_{3(s,\ 0\ K)} + 2MCl_{(s,\ 0\ K)} + 2H_{2(g,\ 0\ K)} + Cl_{2(g,\ 0\ K)} + (\frac{1}{2})O_{2(g,0\ K)} \rightarrow M_2[NpO_2Cl_4]H_2O_{(s,\ 0\ K)}$

ΔH₂: $H_{2(g,\ 0\ K)} \rightarrow H_{2(g,\ 298\ K,\ 1\ bar)}$ **ΔH_2 = 0.088 eV**

ΔH₃: $Cl_{2(g,0\ K)} \rightarrow Cl_{2(g,298\ K,\ 1\ bar)}$ **ΔH_3 = 0.095 eV**

ΔH₄: $O_{2(g,0\ K)} \rightarrow O_{2(g,298\ K,\ 1\ bar)}$ **ΔH_4 = 0.090 eV**

ΔH₅: $(\frac{1}{2})H_{2(g,298\ K,\ 1\ bar)} + (\frac{1}{2})Cl_{2(g,298\ K,\ 1\ bar)} \rightarrow HCl_{(aq,298\ K,\ 1\ bar)}$ **ΔH_5 = -1.732 eV**

ΔH₆: $H_{2(g,\ ,298\ K,\ 1\ bar)} + (\frac{1}{2})O_{2(g,298\ K,\ 1\ bar)} \rightarrow H_2O_{(l,298\ K,1\ bar)}$ **ΔH_6 = -2.962 eV**

Overall: ($\Delta H_1 - 2\Delta H_2 - 2\Delta H_3 - \Delta H_4 - 2\Delta H_5 - \Delta H_6$)

$NpO_{3(s,\ 0\ K)} + 2MCl + 2HCl_{(aq,298\ K,\ 1\ bar)} + H_2O_{(aq,298\ K,\ 1\ bar)} \rightarrow M_2[NpO_2Cl_4]H_2O_{(s,\ 0\ K)}$

Table S14: The thermocycles used for the calculation of formation enthalpies of **Np(VI)-Cs.**

ΔH_1: $NpO_{3(s,\ 0\ K)} + 2CsCl_{(s,\ 0\ K)} + H_{2(g,\ 0\ K)} + Cl_{2(g,\ 0\ K)} \rightarrow Cs_2[NpO_2Cl_4]_{(s,\ 0\ K)} + H_{2(g,\ 0\ K)} + (\frac{1}{2}) O_{2(g,0\ K)}$

ΔH_2: $Cl_{2(g,0\ K)} \rightarrow Cl_{2(g,298\ K,\ 1\ bar)}$ **$\Delta H_3 = 0.095$ eV**

ΔH_3: $O_{2(g,0\ K)} \rightarrow O_{2(g,298\ K,\ 1\ bar)}$ **$\Delta H_4 = 0.090$ eV**

ΔH_4: $(\frac{1}{2})H_{2(g,298\ K,\ 1\ bar)} + (\frac{1}{2})Cl_{2(g,298\ K,\ 1\ bar)} \rightarrow HCl_{(aq,298\ K,\ 1\ bar)}$ **$\Delta H_5 = -1.732$ eV**

ΔH_5: $H_{2(g,\ ,298\ K,\ 1\ bar)} + (\frac{1}{2})O_{2(g,298\ K,\ 1\ bar)} \rightarrow H_2O_{(l,298\ K,1\ bar)}$ **$\Delta H_6 = -2.962$ eV**

Overall: ($\Delta H_1 - \Delta H_2 + (\frac{1}{2})\Delta H_3 - 2\Delta H_4 + \Delta H_5$)

$NpO_{3(s,\ 0\ K)} + 2CsCl_{(s,\ 0\ K)} + 2HCl_{(aq,298\ K,\ 1\ bar)} \rightarrow Cs_2[NpO_2Cl_4]_{(s,\ 0\ K)} + H_2O_{(l,\ 298\ K,\ 1\ bar)}$

Table S15: The thermocycles used for the calculation of formation enthalpies of **Np(V)-Cs**.

ΔH_1: $(\frac{1}{2})Np_2O_{5(s,\ 0\ K)} + 3CsCl_{(s,\ 0\ K)} + H_{2(g,\ 0\ K)} + (\frac{1}{2})Cl_{2(g,\ 0\ K)} \rightarrow Cs_3[NpO_2Cl_4]_{(s,\ 0\ K)} + (\frac{1}{2})H_{2(g,\ 0\ K)} + (\frac{1}{4})O_{2(g,0\ K)}$

ΔH_2: $H_{2(g,\ 0\ K)} \rightarrow H_{2(g,\ 298\ K,\ 1\ bar)}$ **$\Delta H_2 = 0.088$ eV**

ΔH_3: $Cl_{2(g,0\ K)} \rightarrow Cl_{2(g,298\ K,\ 1\ bar)}$ **$\Delta H_3 = 0.095$ eV**

ΔH_4: $O_{2(g,0\ K)} \rightarrow O_{2(g,298\ K,\ 1\ bar)}$ **$\Delta H_4 = 0.090$ eV**

ΔH_5: $(\frac{1}{2})H_{2(g,298\ K,\ 1\ bar)} + (\frac{1}{2})Cl_{2(g,298\ K,\ 1\ bar)} \rightarrow HCl_{(aq,298\ K,\ 1\ bar)}$ **$\Delta H_5 = -1.732$ eV**

ΔH_6: $H_{2(g,\ ,298\ K,\ 1\ bar)} + (\frac{1}{2})O_{2(g,298\ K,\ 1\ bar)} \rightarrow H_2O_{(l,298\ K,1\ bar)}$ **$\Delta H_6 = -2.962$ eV**

Overall: ($\Delta H_1 - (\frac{1}{2})\Delta H2 - (\frac{1}{2})\Delta H_3 + (\frac{1}{4})\Delta H_4 - \Delta H_5 + (\frac{1}{2})\Delta H_6$)

$(\frac{1}{2})Np_2O_{5(s,\ 0\ K)} + 3CsCl_{(s,\ 0\ K)} + HCl_{(aq,298\ K,\ 1\ bar)} \rightarrow Cs_3[NpO_2Cl_4]_{(s,\ 0\ K)} + (\frac{1}{2})H_2O_{(l,\ 298\ K,\ 1\ bar)}$

Table S16: The thermocycles used for the calculation of formation enthalpies of **Np(V)-Morph**.

ΔH₁: $(\frac{1}{2})Np_2O_{5(s,\ 0\ K)} + + 3C_4H_9NO_{(s,\ 0\ K)} + 2H_{2(g,\ 0\ K)} + 2Cl_{2(g,\ 0\ K)} \rightarrow ((C_4H_{10}NO)_3[NpO_2Cl_4])_{(s,0K)} + (\frac{1}{2})H_{2(g,\ 0\ K)} + (\frac{1}{4})O_{2(g,0\ K)}$

ΔH_2: $H_{2(g,\ 0\ K)} \rightarrow H_{2(g,\ 298\ K,\ 1\ bar)}$ **ΔH_2 = 0.088 eV**

ΔH_3: $Cl_{2(g,0\ K)} \rightarrow Cl_{2(g,298\ K,\ 1\ bar)}$ **ΔH_3 = 0.095 eV**

ΔH_4: $O_{2(g,0\ K)} \rightarrow O_{2(g,298\ K,\ 1\ bar)}$ **ΔH_4 = 0.090 eV**

ΔH_5: $(\frac{1}{2})H_{2(g,298\ K,\ 1\ bar)} + (\frac{1}{2})Cl_{2(g,298\ K,\ 1\ bar)} \rightarrow HCl_{(aq,298\ K,\ 1\ bar)}$ **ΔH_5 = -1.732 eV**

ΔH_6: $H_{2(g,\ ,298\ K,\ 1\ bar)} + (\frac{1}{2})O_{2(g,298\ K,\ 1\ bar)} \rightarrow H_2O_{(l,298\ K,1\ bar)}$ **ΔH_6 = -2.962 eV**

Overall: $(\Delta H_1 - (\frac{3}{2})\Delta H_2 - 2\Delta H_3 + (\frac{1}{4})\Delta H_4 - 4\Delta H_5 + (\frac{1}{2})\ \Delta H_6)$

$(\frac{1}{2})Np_2O_{5(s,\ 0\ K)} + 3C_4H_9NO_{(s,\ 0\ K)} + 4HCl_{(aq,298\ K,\ 1\ bar)} \rightarrow ((C_4H_{10}NO)_3[NpO_2Cl_4])_{(s,0K)} + (\frac{1}{2})H_2O_{(l,\ 298\ K,\ 1\ bar)}$

Table S17: The thermocycles used for the calculation of formation enthalpies of **Np(V)-Morph**.

ΔH₁: $(\frac{1}{2})Np_2O_{5(s,\ 0\ K)} + + C_4H_{10}N_{2(s,\ 0\ K)} + (\frac{5}{2})H_{2(g,\ 0\ K)} + (\frac{5}{2})Cl_{2(g,\ 0\ K)} \rightarrow ((C_4H_{12}N_2)_2[NpO_2Cl_4]Cl)_{(s,0K)} + (\frac{1}{2})H_{2(g,\ 0\ K)} + (\frac{1}{4})O_{2(g,0\ K)}$

ΔH_2: $H_{2(g,\ 0\ K)} \rightarrow H_{2(g,\ 298\ K,\ 1\ bar)}$ **ΔH_2 = 0.088 eV**

ΔH_3: $Cl_{2(g,0\ K)} \rightarrow Cl_{2(g,298\ K,\ 1\ bar)}$ **ΔH_3 = 0.095 eV**

ΔH_4: $O_{2(g,0\ K)} \rightarrow O_{2(g,298\ K,\ 1\ bar)}$ **ΔH_4 = 0.090 eV**

ΔH_5: $(\frac{1}{2})H_{2(g,298\ K,\ 1\ bar)} + (\frac{1}{2})Cl_{2(g,298\ K,\ 1\ bar)} \rightarrow HCl_{(aq,298\ K,\ 1\ bar)}$ **ΔH_5 = -1.732 eV**

ΔH_6: $H_{2(g,\ ,298\ K,\ 1\ bar)} + (\frac{1}{2})O_{2(g,298\ K,\ 1\ bar)} \rightarrow H_2O_{(l,298\ K,1\ bar)}$ **ΔH_6 = -2.962 eV**

Overall: $(\Delta H_1 - 2\Delta H_2 - (\frac{5}{2})\Delta H_3 + (\frac{1}{4})\Delta H_4 - 5\Delta H_5 + (\frac{1}{2})\ \Delta H_6)$

$(\frac{1}{2})Np_2O_{5(s,\ 0\ K)} + 3C_4H_{10}N_{2(s,\ 0\ K)} + 5HCl_{(aq,298\ K,\ 1\ bar)} \rightarrow ((C_4H_{12}N_2)_2[NpO_2Cl_4]Cl)_{(s,0K)} + (\frac{1}{2})H_2O_{(l,\ 298\ K,\ 1\ bar)}$

5. Descriptors of ΔH_f

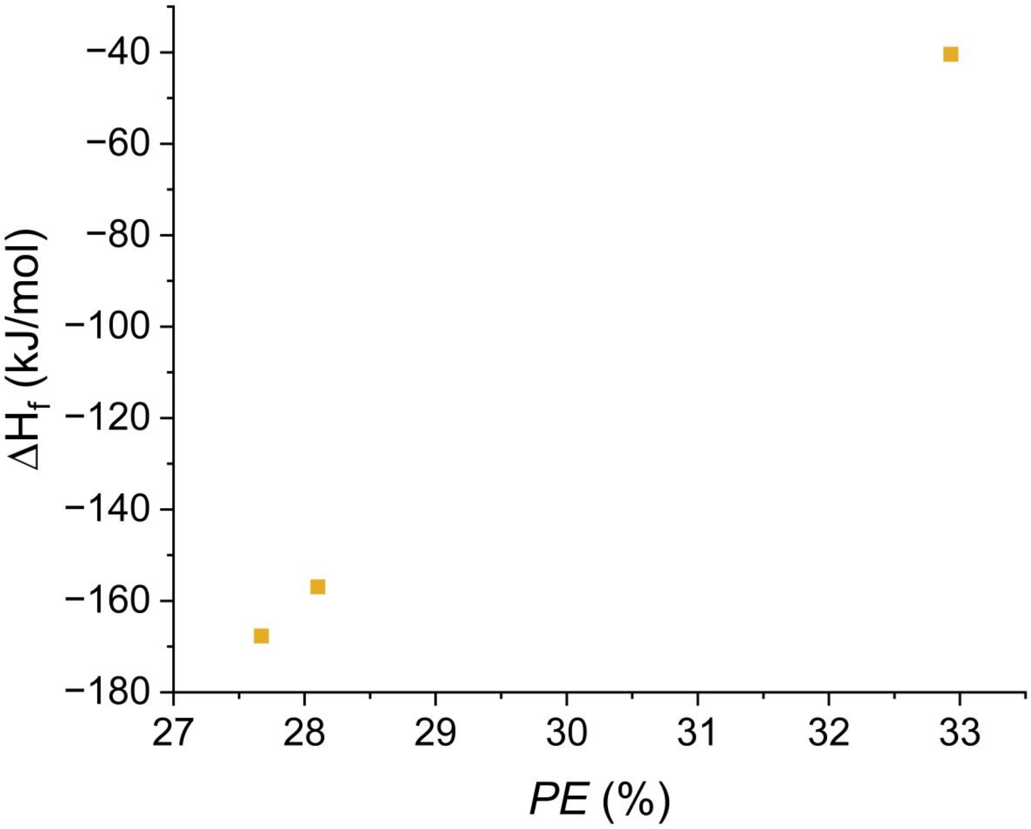

Figure S3: The plot of ΔH_f vs *PE* of uranyl-cation systems

6. Analysis of Non-Covalent Interaction

Np(V)-Morph: Compound has strong participation of axial oxygen for $N-H\cdots O_{yl}$ type bonding with bonding distances and bond orders of 1.671 Å to 0.1949. Strongest $N-H\cdots Cl_{eq}$ interaction has bond distances and bond orders of 2.225 Å and 0.1250 respectively. Strongest $C-H\cdots Cl_{eq}$ bond orders are typically between 0.050-0.0650.

Np(V)-Pipz: Material has uncoordinated chloride atoms in the unit cell that are acting as strong hydrogen bond accepters. With the $[NpO_2Cl_4]^+$ unit, the participation of axial oxygen for hydrogen bonding is prominent where $N-H\cdots O_{yl}$ and $C-H\cdots O_{yl}$ type bonding are observed. $N-H\cdots O_{yl}$ has bond distance of 1.671-1.813 Å (BO = 0.1421-0.1850), while $C-H\cdots O_{yl}$ has bond distance of 2.297 Å (BO = 0.0658). $C/N-H\cdots Cl_{eq}$ interactions are also present.

Np(V)-Cs: Both $Cs\cdots Cl_{eq}$ and $Cs\cdots O_{yl}$ interactions are present in the structure. $Cs\cdots Cl_{eq}$ interaction length varies from 3.440 Å to 3.832 Å and bond orders vary from 0.0588 to 0.1267. $Cs\cdots O_{yl}$ interactions are shorter (from 3.065 Å to 3.179 Å) and weaker (BO = 0.1179 -0.1408).

Np(VI)-Bipy: The strongest hydrogen bonding interactions are $N-H\cdots Cl_{eq}$, where the average bond distances and bond orders are 2.103 Å and 0.159 respectively. Due to overall positive charge of the cation, activation of is $C-H\cdots Cl_{eq}$, and is $C-H\cdots O_{yl}$ hydrogen bonding is observed and agrees with our previous report of $[UO_2Cl_4]^{2-}$ systems.[6] The strongest $C-H\cdots Cl_{eq}$ bond has bond distance and bond order of 2.672 Å and 0.0668 respectively. The $C-H\cdots O_{yl}$ interaction are shorter (2.235 Å) and weaker in terms of bond orders (0.0548).

Np(VI)-Gua: The hydrogen bonding network is like the isomorphic uranyl system.[6] The $\mathrm{N-H\cdots Cl_{eq}}$ type hydrogen bonds have distances ranging from 2.307 Å (BO = 0.1034) to 2.856 Å (BO = 0.0254). The $\mathrm{N-H\cdots O_{yl}}$ type bonding is shorter (2.245 Å) and has BO = 0.0536.

Np(VI)-Morph: Strongest hydrogen bonds are formed between the morpholinium cation linking them into supramolecular chains and these hydrogen bonds have bond distance of 1.786 Å (BO = 0.1277). The neptunyl oxo group only participate in $\mathrm{C-H\cdots O_{yl}}$ type bonding where typical bond distance is 2.291 Å (BO = 0.0691). The hydrogen bonding here is much weaker compared to the previously discussed Np(V) compound **Np(V)-Morph**.

Np(VI)-Apyr: The main hydrogen bond donor is the N atoms, where the -NH_2 interacts with equatorial Cl (2.293 Å and BO = 0.1109) and axial O (2.661 Å and BO = 0.0218). The amine functional group on the ring has similar hydrogen bonding where bond where it interacts with equatorial Cl (2.311 Å and BO = 0.1061) and axial O (2.661 Å and BO = 0.0218).

Np(VI)-Imi: Having two N atoms in the ring, $\mathrm{N-H\cdots Cl_{eq}}$ type hydrogen bonding is prominent with bond distance ranging from 2.133 Å (BO = 0.1532) to 2.198 Å (BO = 0.1332). With charge delocalization, $\mathrm{C-H\cdots Cl_{eq}}$ also present with strongest begin 2.583 Å long (BO = 0.0675). One of the weakest $\mathrm{N-H\cdots O_{yl}}$ hydrogen bonding is observed here (2.670 Å and BO = 0.0206).

Np(VI)-Pipz: Strongest $\mathrm{N-H\cdots O_{yl}}$ bonding is observed here where the bond distances and bond orders are 1.930 Å and 0.1013, respectively. Additionally, strong $\mathrm{N-H\cdots Cl_{eq}}$ type hydrogen bonding is observed (2.137 Å and BO = 0.1445). $\mathrm{C-H\cdots Cl_{eq}}$ bonds are also observed in varying degrees of strength. The overall hydrogen bonding here is much weaker compared to the previously discussed Np(V) compound **Np(V)-Piz.**

Np(VI)-Pyr: Observation are like other neptunyl hybrid materials, where the strongest hydrogen bonds are $\mathrm{N-H\cdots Cl_{eq}}$ types once with distances ranging from 2.417 Å to 2.833 Å and bond orders

ranging from 0.049 to 0.0831. $\mathrm{C-H\cdots Cl_{eq}}$ type bonding is strengthened due to charge delocalization. Hydrogen bonding with axial oxygen happens only through $\mathrm{C-H\cdots O_{yl}}$ bonding (from 2.548 Å, BO = 0.0685 to 2.670 Å, BO = 0.0499).

Np(VI)-K: Compound has both cation interaction and hydrogen bonding present in the lattice. The K^+ interaction with axial oxygen (2.945-3.005Å and BO = 0.0581-0.0681), equatorial Cl (3.221-4.434 Å and BO = 0.0433-0.0806) and water O (2.727-2.780 Å and BO = 0.0919-0.1037). Hydrogen bonding interaction form between water molecules and equatorial chloride (2.244-2.308 Å and BO = 0.0961-0.1166).

Np(VI)-Rb: Both cation interaction and hydrogen bonding are again present, due to the inclusion of a water molecule in the lattice. The Rb^+ interaction with axial oxygen (3.057-3.098Å and BO = 0.0611-0.0692), equatorial Cl (3.345-4.495 Å and BO = 0.0552-0.0922) and water O (2.837-2.948 Å and BO = 0.0937-0.1110). Hydrogen bonding interaction form between water molecules and equatorial chloride (2.317-2.257 Å and BO = 0.0972-0.1165).

***Np(VI)-Cs**:* This is an anhydrous compound that contains $\mathrm{Cs\cdots Cl_{eq}}$ and $\mathrm{Cs\cdots O_{yl}}$ interactions. $\mathrm{Cs\cdots Cl_{eq}}$ interaction length varies from 3.544 Å to 3.679 Å and bond orders vary from 0.0729 to 0.0961. $\mathrm{Cs\cdots O_{yl}}$ interactions are longer (from 3.407 Å to 4.552 Å) and weaker (BO = 0.0713). The overall cation interaction here is much weaker compared to the previously discussed Np(V) compound Np(V)-Cs.

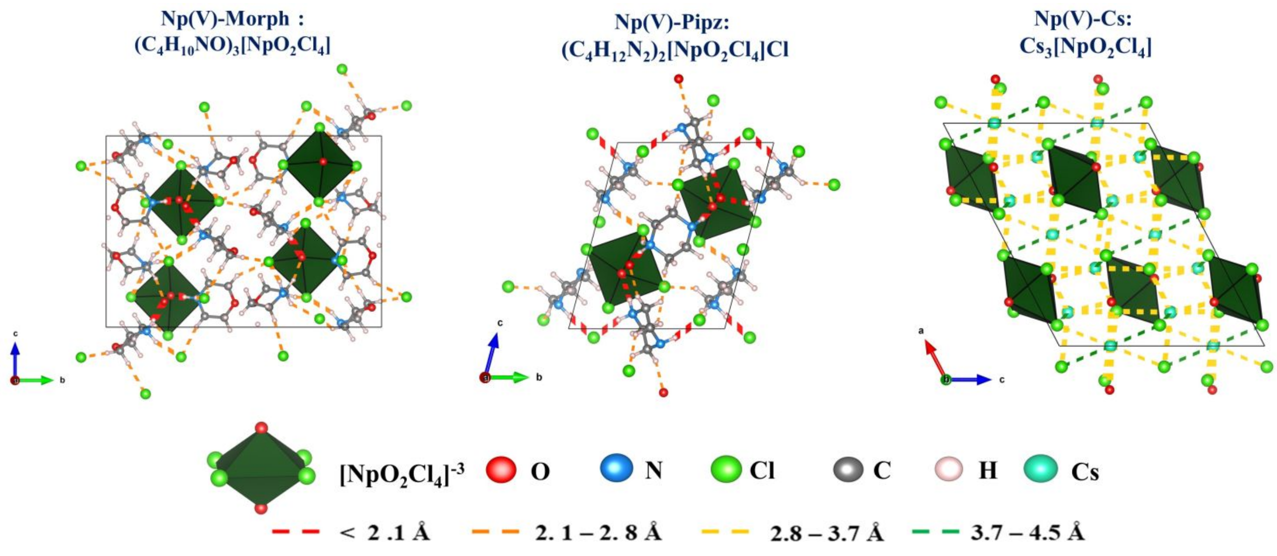

Figure S4: The Non-Covalent Interaction (NCI) network of tested Np(V) compounds.

6.1 Bond order calculation

The bond order between A and j atoms ($B_{A,j}$) is defined as following equation[11-14].

$$B_{A,j} = 2\oint\oint \frac{\rho_A^{\rightarrow avg}(r_A)\cdot \rho_j^{\rightarrow avg}(r'_j)}{\rho^{\rightarrow avg}(\vec{r})\cdot \rho^{\rightarrow avg}(\overrightarrow{r'})} \rho(\vec{r})\rho^{DX_{hole}}(\vec{r},\overrightarrow{r'})\, d^3\vec{r}' d^3\vec{r} \qquad \text{Eq SI 1}$$

$\rho^{\rightarrow avg}(\vec{r})$ = spherically average electron density at r
$\rho_A^{\rightarrow avg}(r_A)$ = spherically average electron density of atom A at r_A distance from A,
$\rho(\vec{r})$ = electron density at r
$\rho^{DX_{hole}}(\vec{r},\overrightarrow{r'})$ = the dressed exchange holes

Table S18: Full list of hydrogen bonding interactions in the unit cell of Np(V)-Morph.

Hydrogen atom	H accepter	Interaction distance (Å)	Bond order
53 H	17 Cl	2.4714968	0.0708
53 H	12 Cl	2.63928929	0.0485
54 H	18 Cl	2.47149706	0.0708
54 H	11 Cl	2.63928929	0.0485
55 H	10 Cl	2.63928929	0.0485
55 H	19 Cl	2.4714968	0.0708
56 H	9 Cl	2.63928929	0.0485
56 H	20 Cl	2.47149706	0.0708
57 H	5 Cl	2.23932304	0.1235
57 H	21 O	2.54627858	0.0253
58 H	6 Cl	2.23932274	0.1235
58 H	22 O	2.54627879	0.0253
59 H	7 Cl	2.23932304	0.1235
59 H	23 O	2.54627858	0.0253
60 H	8 Cl	2.23932274	0.1235
60 H	24 O	2.54627879	0.0253
61 H	21 O	1.68808002	0.1749
62 H	22 O	1.68807988	0.1749
63 H	23 O	1.68808005	0.1749
64 H	24 O	1.68807988	0.1749
65 H	13 Cl	2.27004595	0.1146
65 H	25 O	2.53001633	0.0293
66 H	14 Cl	2.27004594	0.1146
66 H	26 O	2.5300163	0.0293
67 H	15 Cl	2.27004595	0.1146
67 H	27 O	2.53001633	0.0293
68 H	16 Cl	2.27004583	0.1146
68 H	28 O	2.53001626	0.0293
69 H	25 O	1.67009882	0.1949
70 H	26 O	1.67009848	0.1949
71 H	27 O	1.67009882	0.1949
72 H	28 O	1.67009848	0.1949
73 H	20 Cl	2.22462666	0.125
74 H	19 Cl	2.22462682	0.125
75 H	18 Cl	2.22462666	0.125
76 H	17 Cl	2.22462682	0.125
77 H	12 Cl	2.70104379	0.0519
77 H	17 Cl	2.99405543	0.0268
78 H	11 Cl	2.70104379	0.0519
78 H	18 Cl	2.99405505	0.0268
79 H	10 Cl	2.70104375	0.0519
79 H	19 Cl	2.99405545	0.0268
80 H	9 Cl	2.70104375	0.0519
80 H	20 Cl	2.99405514	0.0268
81 H	25 O	2.26304811	0.0663
82 H	26 O	2.26304807	0.0663
83 H	27 O	2.26304811	0.0663
84 H	28 O	2.26304811	0.0663
85 H	29 O	2.08252247	0.0274
85 H	38 O	2.66436349	0.0266
86 H	30 O	2.08252204	0.0274
86 H	37 O	2.66436348	0.0266
87 H	31 O	2.08252247	0.0274
87 H	40 O	2.66436349	0.0266
88 H	32 O	2.08252204	0.0274
88 H	39 O	2.66436348	0.0266
89 H	5 Cl	2.94980909	0.035
89 H	29 O	2.05153806	0.0328
90 H	6 Cl	2.94980925	0.035
90 H	30 O	2.05153781	0.0328
91 H	31 O	2.05153806	0.0328
91 H	7 Cl	2.94980909	0.035
92 H	32 O	2.05153781	0.0328
92 H	8 Cl	2.94980925	0.035
93 H	29 O	2.05109306	0.0315
94 H	30 O	2.05109322	0.0315
95 H	31 O	2.0510932	0.0315
96 H	32 O	2.05109322	0.0315
97 H	29 O	2.0834643	0.0275
98 H	30 O	2.08346443	0.0275
99 H	31 O	2.08346425	0.0275
100 H	32 O	2.08346443	0.0275
101 H	35 O	2.64065685	0.0237
102 H	36 O	2.64065686	0.0237
103 H	33 O	2.64065687	0.0237
104 H	34 O	2.64065686	0.0237
105 H	16 Cl	2.71788728	0.0582
106 H	15 Cl	2.71788711	0.0582
107 H	14 Cl	2.71788711	0.0582
108 H	13 Cl	2.71788711	0.0582

109 H	13 Cl	2.93837604	0.0351
110 H	14 Cl	2.93837614	0.0351
111 H	15 Cl	2.93837614	0.0351
112 H	16 Cl	2.93837614	0.0351
113 H	20 Cl	2.58029435	0.0711
114 H	19 Cl	2.58029482	0.0711
115 H	18 Cl	2.58029434	0.0711
116 H	17 Cl	2.58029482	0.0711
117 H	13 Cl	2.97872176	0.0283
117 H	33 O	2.07479005	0.0282
118 H	14 Cl	2.97872186	0.0283
118 H	34 O	2.07479	0.0282
119 H	15 Cl	2.97872176	0.0283
119 H	35 O	2.07479009	0.0282
120 H	16 Cl	2.97872193	0.0283
120 H	36 O	2.07478992	0.0282
121 H	32 O	2.45928555	0.0381
121 H	33 O	2.04436808	0.0339
121 H	34 O	2.59284451	0.0218
122 H	31 O	2.45928521	0.0381
122 H	34 O	2.04436806	0.0339
122 H	33 O	2.59284465	0.0218
123 H	30 O	2.45928561	0.0381
123 H	36 O	2.59284448	0.0218
123 H	35 O	2.04436809	0.0339
124 H	29 O	2.45928521	0.0381
124 H	35 O	2.59284468	0.0218
124 H	36 O	2.04436806	0.0339
125 H	33 O	2.04015501	0.0332
125 H	7 Cl	2.65496079	0.0615
126 H	34 O	2.04015521	0.0332
126 H	8 Cl	2.65496073	0.0615
127 H	5 Cl	2.65496068	0.0615
127 H	35 O	2.04015522	0.0332
128 H	6 Cl	2.65496073	0.0615
128 H	36 O	2.04015521	0.0332
129 H	13 Cl	2.97392352	0.0291
129 H	33 O	2.08210663	0.0277
130 H	14 Cl	2.97392342	0.0291
130 H	34 O	2.08210643	0.0277
131 H	15 Cl	2.97392352	0.0291
131 H	35 O	2.08210667	0.0277
132 H	16 Cl	2.97392335	0.0291

132 H	36 O	2.08210651	0.0277
133 H	12 Cl	2.79399746	0.0464
134 H	11 Cl	2.79399762	0.0464
135 H	10 Cl	2.79399764	0.0464
136 H	9 Cl	2.79399761	0.0464
137 H	13 Cl	2.73583603	0.052
138 H	14 Cl	2.73583603	0.052
139 H	15 Cl	2.73583603	0.052
140 H	16 Cl	2.73583603	0.052
141 H	5 Cl	3.16611617	0.0211
141 H	38 O	2.55257938	0.0323
142 H	6 Cl	3.16611634	0.0211
142 H	37 O	2.55257938	0.0323
143 H	7 Cl	3.16611617	0.0211
143 H	40 O	2.55257938	0.0323
144 H	8 Cl	3.16611634	0.0211
144 H	39 O	2.55257938	0.0323
145 H	9 Cl	2.76798742	0.0453
146 H	10 Cl	2.76798742	0.0453
147 H	11 Cl	2.76798742	0.0453
148 H	12 Cl	2.76798742	0.0453
149 H	16 Cl	2.7336342	0.0547
149 H	37 O	2.07795418	0.0288
150 H	15 Cl	2.73363454	0.0547
150 H	38 O	2.07795418	0.0288
151 H	14 Cl	2.7336342	0.0547
151 H	39 O	2.07795418	0.0288
152 H	13 Cl	2.73363454	0.0547
152 H	40 O	2.07795418	0.0288
153 H	6 Cl	2.64244209	0.0664
153 H	37 O	2.04645202	0.0345
154 H	5 Cl	2.64244224	0.0664
154 H	38 O	2.04645202	0.0345
155 H	39 O	2.04645202	0.0345
155 H	8 Cl	2.64244209	0.0664
156 H	40 O	2.04645202	0.0345
156 H	7 Cl	2.64244224	0.0664
157 H	37 O	2.04413146	0.0339
158 H	38 O	2.04413146	0.0339
159 H	39 O	2.04413146	0.0339
160 H	40 O	2.04413146	0.0339
161 H	16 Cl	3.19339067	0.0205
161 H	37 O	2.08268985	0.029

162 H	15 Cl	3.19339108	0.0205
162 H	38 O	2.08268985	0.029
163 H	14 Cl	3.19339067	0.0205
163 H	39 O	2.08268985	0.029
164 H	13 Cl	3.19339108	0.0205
164 H	40 O	2.08268985	0.029
165 H	9 Cl	2.68146975	0.0566
165 H	21 O	2.67172706	0.029
166 H	10 Cl	2.68146982	0.0566
166 H	22 O	2.67172631	0.029
167 H	23 O	2.67172706	0.029
167 H	11 Cl	2.68146975	0.0566
168 H	24 O	2.67172631	0.029
168 H	12 Cl	2.68146982	0.0566
169 H	5 Cl	2.99822448	0.025
170 H	6 Cl	2.99822451	0.025
171 H	7 Cl	2.99822448	0.025
172 H	8 Cl	2.99822451	0.025

Table S19: Full list of hydrogen bonding interactions in the unit cell of Np(V)-Pipz.

Hydrogen atom	H accepter	Interaction distance (Å)	Bond order
25 H	12 Cl	2.062677	0.1632
26 H	11 Cl	2.062677	0.1632
27 H	4 Cl	2.875954	0.0261
27 H	11 Cl	2.431971	0.0692
27 H	5 Cl	2.83435	0.0306
28 H	3 Cl	2.875954	0.0261
28 H	12 Cl	2.431971	0.0692
28 H	6 Cl	2.83435	0.0306
29 H	15 O	1.825872	0.1421
30 H	16 O	1.825872	0.1421
31 H	11 Cl	2.03781	0.1717
32 H	12 Cl	2.03781	0.1717
33 H	7 Cl	2.227965	0.1214
33 H	15 O	2.56933	0.0274
34 H	8 Cl	2.227965	0.1214
34 H	16 O	2.56933	0.0274
35 H	13 O	1.670464	0.185
36 H	14 O	1.670464	0.185
37 H	10 Cl	2.731952	0.0326
37 H	16 O	1.813466	0.1407
38 H	9 Cl	2.731953	0.0326
38 H	15 O	1.813466	0.1407
39 H	7 Cl	2.140233	0.1461
40 H	8 Cl	2.140233	0.1461
41 H	4 Cl	2.558073	0.0686
41 H	7 Cl	3.035622	0.0217
42 H	3 Cl	2.558073	0.0686
42 H	8 Cl	3.035622	0.0217
43 H	5 Cl	3.061156	0.0231
43 H	11 Cl	2.64331	0.0538
44 H	6 Cl	3.061156	0.0231
44 H	12 Cl	2.643309	0.0538
45 H	12 Cl	2.814977	0.0368
46 H	11 Cl	2.814977	0.0368
47 H	13 O	2.29704	0.0658
48 H	14 O	2.29704	0.0658
49 H	7 Cl	2.834269	0.0385
49 H	8 Cl	2.91539	0.0366

50 H	7 Cl	2.91539	0.0366
50 H	8 Cl	2.834268	0.0385
51 H	9 Cl	2.789436	0.041
52 H	10 Cl	2.789436	0.041
53 H	4 Cl	3.132815	0.0222
53 H	5 Cl	2.753095	0.047
54 H	3 Cl	3.132815	0.0222
54 H	6 Cl	2.753096	0.047
55 H	3 Cl	2.667282	0.0597
55 H	12 Cl	3.048694	0.0211
55 H	6 Cl	2.985163	0.0294
56 H	4 Cl	2.667282	0.0597
56 H	11 Cl	3.048695	0.0211
56 H	5 Cl	2.985164	0.0294
57 H	6 Cl	2.883166	0.0369
57 H	12 Cl	2.932197	0.0271
58 H	5 Cl	2.883166	0.0369
58 H	11 Cl	2.932198	0.0271
59 H	10 Cl	2.850993	0.0364
59 H	11 Cl	3.017697	0.0222
60 H	9 Cl	2.850993	0.0364
60 H	12 Cl	3.017697	0.0222
61 H	9 Cl	2.848107	0.0391
61 H	14 O	2.650844	0.0276
61 H	10 Cl	2.735726	0.0485
62 H	10 Cl	2.848107	0.0391
62 H	13 O	2.650844	0.0276
62 H	9 Cl	2.735726	0.0485
63 H	6 Cl	2.998399	0.0242
64 H	5 Cl	2.9984	0.0242
65 H	11 Cl	2.795643	0.0372
65 H	6 Cl	2.839396	0.0408
66 H	12 Cl	2.795643	0.0372
66 H	5 Cl	2.839396	0.0408
67 H	4 Cl	3.038876	0.0214
67 H	7 Cl	2.93643	0.0281
68 H	3 Cl	3.038876	0.0214
68 H	8 Cl	2.93643	0.0281
69 H	8 Cl	2.838378	0.0357
70 H	7 Cl	2.838378	0.0357
71 H	3 Cl	2.700895	0.0539
72 H	4 Cl	2.700895	0.0539

Table S20: Full list of cation interactions in the unit cell of Np(V)-Cs.

Metal ion	Cl or O atom	Interaction distance (Å)	Bond order
5 Cs	22 Cl	3.440082	0.1267
5 Cs	24 Cl	3.440082	0.1267
5 Cs	29 Cl	3.643778	0.0852
5 Cs	30 Cl	3.832269	0.0558
5 Cs	31 Cl	3.643778	0.0852
5 Cs	33 O	3.065002	0.1408
5 Cs	35 O	3.065002	0.1408
5 Cs	32 Cl	3.832269	0.0558
6 Cs	21 Cl	3.440082	0.1267
6 Cs	23 Cl	3.440082	0.1267
6 Cs	29 Cl	3.832269	0.0558
6 Cs	30 Cl	3.643778	0.0852
6 Cs	32 Cl	3.643778	0.0852
6 Cs	34 O	3.065002	0.1408
6 Cs	36 O	3.065002	0.1408
6 Cs	31 Cl	3.832269	0.0558
7 Cs	20 Cl	3.440082	0.1267
7 Cs	25 Cl	3.643778	0.0852
7 Cs	18 Cl	3.440082	0.1267
7 Cs	26 Cl	3.832269	0.0558
7 Cs	27 Cl	3.643778	0.0852
7 Cs	28 Cl	3.832269	0.0558
7 Cs	37 O	3.065002	0.1408
7 Cs	39 O	3.065002	0.1408
8 Cs	17 Cl	3.440082	0.1267
8 Cs	28 Cl	3.643778	0.0852
8 Cs	19 Cl	3.440082	0.1267
8 Cs	25 Cl	3.832269	0.0558
8 Cs	26 Cl	3.643778	0.0852
8 Cs	27 Cl	3.832269	0.0558
8 Cs	38 O	3.065002	0.1408
8 Cs	40 O	3.065002	0.1408
9 Cs	20 Cl	3.539841	0.108
9 Cs	25 Cl	4.244448	0.0215
9 Cs	31 Cl	3.760151	0.0578
9 Cs	31 Cl	3.760151	0.0693

9 Cs	33 O	3.154773	0.1179
9 Cs	22 Cl	3.483756	0.1193
9 Cs	23 Cl	3.513744	0.1116
9 Cs	31 Cl	3.844923	0.0578
9 Cs	31 Cl	3.844923	0.0693
9 Cs	32 Cl	3.5516	0.1144
9 Cs	39 O	3.179314	0.1088
10 Cs	19 Cl	3.53984	0.108
10 Cs	26 Cl	4.244448	0.0215
10 Cs	32 Cl	3.76015	0.0693
10 Cs	32 Cl	3.76015	0.0578
10 Cs	34 O	3.154773	0.1179
10 Cs	21 Cl	3.483756	0.1193
10 Cs	24 Cl	3.513744	0.1116
10 Cs	31 Cl	3.551599	0.1144
10 Cs	32 Cl	3.844923	0.0693
10 Cs	32 Cl	3.844923	0.0578
10 Cs	40 O	3.179314	0.1088
11 Cs	18 Cl	3.539841	0.108
11 Cs	21 Cl	3.513744	0.1116
11 Cs	27 Cl	4.244448	0.0215
11 Cs	29 Cl	3.76015	0.0578
11 Cs	29 Cl	3.76015	0.0693
11 Cs	30 Cl	3.551599	0.1144
11 Cs	35 O	3.154773	0.1179
11 Cs	24 Cl	3.483756	0.1193
11 Cs	29 Cl	3.844923	0.0578
11 Cs	29 Cl	3.844923	0.0693
11 Cs	37 O	3.179314	0.1088
12 Cs	17 Cl	3.539841	0.108
12 Cs	22 Cl	3.513744	0.1116
12 Cs	28 Cl	4.244448	0.0215
12 Cs	29 Cl	3.5516	0.1144
12 Cs	30 Cl	3.76015	0.0693
12 Cs	30 Cl	3.76015	0.0578
12 Cs	36 O	3.154773	0.1179
12 Cs	23 Cl	3.483756	0.1193
12 Cs	30 Cl	3.844923	0.0693
12 Cs	30 Cl	3.844923	0.0578
12 Cs	38 O	3.179314	0.1088
13 Cs	18 Cl	3.483756	0.1193
13 Cs	24 Cl	3.53984	0.108
13 Cs	27 Cl	3.844923	0.0578

13 Cs	27 Cl	3.844923	0.0693
13 Cs	29 Cl	4.244448	0.0215
13 Cs	35 O	3.179314	0.1088
13 Cs	37 O	3.154773	0.1179
13 Cs	19 Cl	3.513744	0.1116
13 Cs	27 Cl	3.76015	0.0578
13 Cs	27 Cl	3.76015	0.0693
13 Cs	28 Cl	3.551599	0.1144
14 Cs	17 Cl	3.483756	0.1193
14 Cs	23 Cl	3.539841	0.108
14 Cs	28 Cl	3.844923	0.0693
14 Cs	28 Cl	3.844923	0.0578
14 Cs	30 Cl	4.244447	0.0215
14 Cs	36 O	3.179314	0.1088
14 Cs	38 O	3.154773	0.1179
14 Cs	20 Cl	3.513744	0.1116
14 Cs	27 Cl	3.5516	0.1144
14 Cs	28 Cl	3.76015	0.0693
14 Cs	28 Cl	3.76015	0.0578
15 Cs	17 Cl	3.513744	0.1116
15 Cs	20 Cl	3.483756	0.1193
15 Cs	22 Cl	3.539841	0.108
15 Cs	25 Cl	3.844923	0.0578
15 Cs	25 Cl	3.844923	0.0693
15 Cs	31 Cl	4.244447	0.0215
15 Cs	33 O	3.179314	0.1088
15 Cs	39 O	3.154773	0.1179
15 Cs	25 Cl	3.76015	0.0578
15 Cs	25 Cl	3.76015	0.0693
15 Cs	26 Cl	3.5516	0.1144
16 Cs	18 Cl	3.513744	0.1116
16 Cs	19 Cl	3.483756	0.1193
16 Cs	21 Cl	3.53984	0.108
16 Cs	26 Cl	3.844923	0.0693
16 Cs	26 Cl	3.844923	0.0578
16 Cs	32 Cl	4.244448	0.0215
16 Cs	34 O	3.179314	0.1088
16 Cs	40 O	3.154773	0.1179
16 Cs	25 Cl	3.551599	0.1144
16 Cs	26 Cl	3.76015	0.0693
16 Cs	26 Cl	3.76015	0.0578

Table S21: Full list of hydrogen bonding interactions in the unit cell of Np(VI)-Bipy.

Hydrogen atom	H accepter	Interaction distance (Å)	Bond order
5 H	114 Cl	2.1028594	0.159
6 H	113 Cl	2.1028596	0.159
7 H	116 Cl	2.1028595	0.159
8 H	115 Cl	2.1028595	0.159
9 H	93 O	2.3543508	0.0548
9 H	105 Cl	2.9004523	0.0319
10 H	94 O	2.3543509	0.0548
10 H	106 Cl	2.9004522	0.0319
11 H	95 O	2.3543507	0.0548
11 H	107 Cl	2.9004522	0.0319
12 H	96 O	2.354351	0.0548
12 H	108 Cl	2.9004522	0.0319
13 H	107 Cl	2.6933737	0.0502
13 H	111 Cl	3.0292452	0.0277
13 H	115 Cl	3.1370044	0.0203
14 H	108 Cl	2.6933739	0.0502
14 H	112 Cl	3.029245	0.0277
14 H	116 Cl	3.1370043	0.0203
15 H	105 Cl	2.6933738	0.0502
15 H	109 Cl	3.029245	0.0277
15 H	113 Cl	3.1370042	0.0203
16 H	106 Cl	2.6933739	0.0502
16 H	110 Cl	3.0292453	0.0277
16 H	114 Cl	3.1370046	0.0203
17 H	101 Cl	2.6101839	0.0692
18 H	102 Cl	2.6101837	0.0692
19 H	103 Cl	2.6101839	0.0692
20 H	104 Cl	2.6101839	0.0692
21 H	107 Cl	2.1390428	0.152
22 H	108 Cl	2.1390428	0.152
23 H	105 Cl	2.139043	0.152
24 H	106 Cl	2.1390428	0.152
25 H	110 Cl	2.6717738	0.0668
26 H	109 Cl	2.6717737	0.0668
27 H	112 Cl	2.6717739	0.0668
28 H	111 Cl	2.6717739	0.0668
29 H	96 O	2.8310904	0.0209
29 H	103 Cl	3.0921197	0.0214
30 H	95 O	2.8310907	0.0209
30 H	104 Cl	3.0921197	0.0214
31 H	94 O	2.8310905	0.0209
31 H	101 Cl	3.0921197	0.0214
32 H	93 O	2.8310907	0.0209
32 H	102 Cl	3.0921197	0.0214
33 H	97 O	2.2694797	0.0655
33 H	103 Cl	2.9091224	0.032
33 H	113 Cl	3.0694025	0.0214
34 H	98 O	2.2694792	0.0655
34 H	104 Cl	2.9091225	0.032
34 H	114 Cl	3.0694022	0.0214
35 H	99 O	2.2694794	0.0655
35 H	101 Cl	2.9091227	0.032
35 H	115 Cl	3.0694022	0.0214
36 H	100 O	2.2694792	0.0655
36 H	102 Cl	2.9091225	0.032
36 H	116 Cl	3.0694025	0.0214
37 H	96 O	2.239473	0.0704
37 H	115 Cl	3.0040208	0.0232
38 H	95 O	2.2394733	0.0704
38 H	116 Cl	3.0040206	0.0232
39 H	94 O	2.239473	0.0704
39 H	113 Cl	3.0040206	0.0232
40 H	93 O	2.2394733	0.0704
40 H	114 Cl	3.0040207	0.0232
41 H	106 Cl	2.5682195	0.0739
42 H	105 Cl	2.568219	0.0739
43 H	108 Cl	2.5682196	0.0739
44 H	107 Cl	2.5682191	0.0739

Table S22: Full list of hydrogen bonding interactions in the unit cell of Np(VI)-Gua.

Hydrogen atom	H accepter	Interaction distance (Å)	Bond order
27 H	3 Cl	2.522565514	0.0661
27 H	9 Cl	2.782495776	0.0359
28 H	4 Cl	2.522565459	0.0661
28 H	10 Cl	2.782496058	0.0359
29 H	7 Cl	2.782495776	0.0359
29 H	5 Cl	2.522565514	0.0661
30 H	8 Cl	2.782496058	0.0359
30 H	6 Cl	2.522565459	0.0661
31 H	7 Cl	2.856800607	0.0254
31 H	14 O	2.245533013	0.0536
32 H	8 Cl	2.856800553	0.0254
32 H	13 O	2.245532949	0.0536
33 H	9 Cl	2.856800607	0.0254
33 H	12 O	2.245533013	0.0536
34 H	10 Cl	2.856800553	0.0254
34 H	11 O	2.245532949	0.0536
35 H	5 Cl	2.576096489	0.055
35 H	10 Cl	2.756182788	0.0348
36 H	6 Cl	2.576096489	0.055
36 H	9 Cl	2.756182977	0.0348
37 H	3 Cl	2.576096489	0.055
37 H	8 Cl	2.756182788	0.0348
38 H	4 Cl	2.576096489	0.055
38 H	7 Cl	2.756182977	0.0348
39 H	7 Cl	2.306653804	0.1034
40 H	8 Cl	2.306653778	0.1034
41 H	9 Cl	2.30665383	0.1034
42 H	10 Cl	2.306653778	0.1034
43 H	6 Cl	2.456949878	0.0762
44 H	5 Cl	2.456949878	0.0762
45 H	4 Cl	2.456949878	0.0762
46 H	3 Cl	2.456949878	0.0762
47 H	3 Cl	2.579947446	0.0574
47 H	8 Cl	2.711865029	0.0461
48 H	4 Cl	2.579947446	0.0574
48 H	7 Cl	2.711864853	0.0461
49 H	5 Cl	2.579947446	0.0574
49 H	10 Cl	2.711865029	0.0461
50 H	6 Cl	2.579947446	0.0574
50 H	9 Cl	2.711864853	0.0461

Table S23: Full list of hydrogen bonding interactions in the unit cell of Np(VI)-Morph.

Hydrogen atom	H accepter	Interaction distance (Å)	Bond order
23 H	13 O	1.786686754	0.1277
24 H	14 O	1.786686745	0.1277
25 H	11 O	1.786686754	0.1277
26 H	12 O	1.786686745	0.1277
27 H	5 Cl	2.916803964	0.0233
27 H	9 Cl	2.371033053	0.0907
27 H	10 Cl	2.880186658	0.0293
28 H	6 Cl	2.916803757	0.0233
28 H	9 Cl	2.880186579	0.0293
28 H	10 Cl	2.371033008	0.0907
29 H	3 Cl	2.916803757	0.0233
29 H	7 Cl	2.371033008	0.0907
29 H	8 Cl	2.880186579	0.0293
30 H	4 Cl	2.916803923	0.0233
30 H	8 Cl	2.371033008	0.0907
30 H	7 Cl	2.880186579	0.0293
31 H	6 Cl	3.095059165	0.0228
31 H	11 O	2.067557024	0.0275
32 H	5 Cl	3.095059359	0.0228
32 H	12 O	2.067556574	0.0275
33 H	4 Cl	3.095059346	0.0228
33 H	13 O	2.067556905	0.0275
34 H	3 Cl	3.095059355	0.0228
34 H	14 O	2.067556611	0.0275
35 H	9 Cl	3.075095205	0.0216
35 H	5 Cl	2.723476148	0.045
36 H	10 Cl	3.07509523	0.0216
36 H	6 Cl	2.72347591	0.045
37 H	3 Cl	2.72347591	0.045
37 H	7 Cl	3.07509523	0.0216
38 H	4 Cl	2.723476089	0.045
38 H	8 Cl	3.07509523	0.0216
39 H	6 Cl	2.854488564	0.0348
39 H	10 Cl	2.678968988	0.0541
40 H	5 Cl	2.854488735	0.0348
40 H	9 Cl	2.678968683	0.0541
41 H	8 Cl	2.678969105	0.0541
41 H	4 Cl	2.854488775	0.0348
42 H	7 Cl	2.678968665	0.0541
42 H	3 Cl	2.854488533	0.0348
43 H	7 Cl	2.689944825	0.0605
43 H	3 Cl	3.066822031	0.0224
43 H	11 O	2.055224159	0.0314
44 H	4 Cl	3.066822031	0.0224
44 H	12 O	2.055224286	0.0314
44 H	8 Cl	2.689944923	0.0605
45 H	9 Cl	2.689944923	0.0605
45 H	13 O	2.055224148	0.0314
45 H	5 Cl	3.066822031	0.0224
46 H	10 Cl	2.689944825	0.0605
46 H	14 O	2.055224297	0.0314
46 H	6 Cl	3.066822031	0.0224
47 H	11 O	2.071441289	0.0288
47 H	4 Cl	2.783161005	0.0445
48 H	12 O	2.071441397	0.0288
48 H	3 Cl	2.783161076	0.0445
49 H	6 Cl	2.783161076	0.0445
49 H	13 O	2.071441289	0.0288
50 H	5 Cl	2.783161005	0.0445
50 H	14 O	2.071441397	0.0288
55 H	17 O	2.292132003	0.0691
56 H	18 O	2.292132003	0.0691
57 H	15 O	2.292132003	0.0691
58 H	16 O	2.292132003	0.0691

Table S24: Full list of hydrogen bonding interactions in the unit cell of Np(VI)-Apyr.

Hydrogen atom	H accepter	Interaction distance (Å)	Bond order
12 H	4 Cl	2.293251074	0.1109
13 H	5 Cl	2.293251047	0.1109
14 H	3 Cl	2.311633853	0.1061
14 H	6 O	2.661247879	0.0218
15 H	2 Cl	2.311633701	0.1061
15 H	7 O	2.661247986	0.0218
16 H	4 Cl	2.231791217	0.1255
17 H	5 Cl	2.231791157	0.1255
18 H	3 Cl	2.853769199	0.0361
19 H	2 Cl	2.853769127	0.0361
20 H	2 Cl	2.830116263	0.0376
20 H	3 Cl	2.799335601	0.0404
21 H	2 Cl	2.799335427	0.0404
21 H	3 Cl	2.830116501	0.0376
22 H	5 Cl	2.6314511	0.0672
23 H	4 Cl	2.631451032	0.0672
24 H	6 O	2.461209633	0.0479
24 H	2 Cl	3.020888257	0.0234
25 H	7 O	2.46120976	0.0479
25 H	3 Cl	3.020888191	0.0234

Table S25: Full list of hydrogen bonding interactions in the unit cell of Np(VI)-Imi.

Hydrogen atom	H accepter	Interaction distance (Å)	Bond order
23 H	5 Cl	2.175787213	0.1402
24 H	6 Cl	2.175787295	0.1402
25 H	13 O	2.463003749	0.0469
26 H	14 O	2.463003681	0.0469
27 H	6 Cl	2.623750418	0.0643
27 H	8 Cl	2.723431193	0.0526
27 H	4 Cl	2.967646153	0.0303
28 H	5 Cl	2.623750191	0.0643
28 H	7 Cl	2.723431257	0.0526
28 H	3 Cl	2.967646208	0.0303
29 H	3 Cl	2.752873789	0.0395
29 H	11 O	2.393403137	0.0474
30 H	4 Cl	2.752873789	0.0395
30 H	12 O	2.393403137	0.0474
31 H	9 Cl	2.948306279	0.031
31 H	6 Cl	2.722454655	0.0514
32 H	10 Cl	2.948306279	0.031
32 H	5 Cl	2.722454538	0.0514
33 H	7 Cl	2.917651221	0.0404
34 H	8 Cl	2.917651221	0.0404
35 H	10 Cl	2.583176588	0.0675
36 H	9 Cl	2.583176588	0.0675
37 H	8 Cl	2.198484767	0.1332
38 H	7 Cl	2.198484767	0.1332
39 H	3 Cl	2.132573306	0.1532
39 H	14 O	2.670418475	0.0206
40 H	4 Cl	2.132573306	0.1532
40 H	13 O	2.670418612	0.0206
41 H	9 Cl	2.165366451	0.1369
42 H	10 Cl	2.165366563	0.1369

Table S26: Full list of hydrogen bonding interactions in the unit cell of Np(VI)-Piz.

Hydrogen atom	H accepter	Interaction distance (Å)	Bond order
10 H	6 O	1.930302038	0.1013
10 H	5 Cl	2.812691987	0.0262
11 H	7 O	1.930301714	0.1013
11 H	4 Cl	2.812691993	0.0262
12 H	3 Cl	2.137105782	0.1445
12 H	7 O	2.626607804	0.0203
13 H	2 Cl	2.137105584	0.1445
13 H	6 O	2.626607615	0.0203
14 H	5 Cl	3.035395172	0.0306
14 H	2 Cl	2.919356961	0.0308
15 H	4 Cl	3.035395104	0.0306
15 H	3 Cl	2.919357108	0.0308
16 H	4 Cl	3.214874221	0.0205
16 H	5 Cl	3.070344106	0.0238
16 H	7 O	2.628190762	0.0273
17 H	5 Cl	3.214874187	0.0205
17 H	4 Cl	3.070344039	0.0238
17 H	6 O	2.628190654	0.0273
18 H	5 Cl	2.809620059	0.0373
18 H	2 Cl	2.611212924	0.056
19 H	4 Cl	2.809619984	0.0373
19 H	3 Cl	2.611212798	0.056
20 H	2 Cl	2.893360366	0.0333
20 H	4 Cl	3.071491124	0.0236
20 H	3 Cl	2.721239086	0.0515
21 H	3 Cl	2.893360584	0.0333
21 H	5 Cl	3.071491184	0.0236
21 H	2 Cl	2.721239095	0.0515

Table S27: Full list of hydrogen bonding interactions in the unit cell of Np(VI)-Pyr.

Hydrogen atom	H accepter	Interaction distance (Å)	Bond order
19 H	5 Cl	2.662028768	0.049
19 H	8 Cl	2.417494909	0.0831
20 H	6 Cl	2.662028596	0.049
20 H	7 Cl	2.417495105	0.0831
21 H	10 Cl	2.982773694	0.0285
21 H	8 Cl	2.739772327	0.0449
22 H	9 Cl	2.982773717	0.0285
22 H	7 Cl	2.73977277	0.0449
23 H	3 Cl	2.584303846	0.0685
23 H	12 O	2.548208255	0.0495
24 H	4 Cl	2.584304065	0.0685
24 H	11 O	2.548208362	0.0495
25 H	9 Cl	2.853218474	0.0358
26 H	10 Cl	2.853218159	0.0358
27 H	6 Cl	2.907749755	0.0367
27 H	9 Cl	2.742090655	0.0464
28 H	5 Cl	2.907749405	0.0367
28 H	10 Cl	2.742090371	0.0464
29 H	5 Cl	2.719615988	0.0521
29 H	4 Cl	2.849662454	0.0368
30 H	6 Cl	2.719615839	0.0521
30 H	3 Cl	2.849662547	0.0368
31 H	4 Cl	2.833413581	0.0319
31 H	5 Cl	2.593745712	0.0574
31 H	8 Cl	2.53356406	0.0634
32 H	3 Cl	2.833413789	0.0319
32 H	6 Cl	2.593745634	0.0574
32 H	7 Cl	2.533563975	0.0634
33 H	5 Cl	2.76410096	0.045
33 H	10 Cl	2.615394911	0.0626
34 H	6 Cl	2.764101126	0.045
34 H	9 Cl	2.61539474	0.0626
35 H	13 O	2.670204585	0.0499
35 H	6 Cl	2.99538339	0.0258
36 H	14 O	2.670204444	0.0499
36 H	5 Cl	2.995383487	0.0258
37 H	3 Cl	2.587483449	0.0682
37 H	11 O	2.650584922	0.0488
38 H	4 Cl	2.587483397	0.0682
38 H	12 O	2.65058521	0.0488
39 H	9 Cl	3.031624724	0.0273
39 H	7 Cl	2.874361399	0.0351
40 H	10 Cl	3.03162476	0.0273
40 H	8 Cl	2.874361331	0.0351
41 H	8 Cl	2.85890178	0.0362
41 H	14 O	2.670126578	0.0477
41 H	7 Cl	2.912788932	0.033
42 H	7 Cl	2.858901635	0.0362
42 H	13 O	2.670126579	0.0477
42 H	8 Cl	2.91278895	0.033

Table S28: Full list of cation interactions in the unit cell of Np(VI)-K

Metal ion	Cl or O atom	Interaction distance (Å)	Bond order
2 K	5 Cl	4.204948541	0.0806
2 K	7 Cl	3.514671105	0.0433
2 K	7 Cl	3.514671105	0.0465
2 K	8 O	2.9453718	0.0681
2 K	4 Cl	3.291918984	0.0636
2 K	5 Cl	3.221347215	0.0806
2 K	5 Cl	4.434447623	0.0806
2 K	6 Cl	3.263245115	0.0767
2 K	7 Cl	3.486860368	0.0433
2 K	7 Cl	3.486860368	0.0465
2 K	9 O	3.005371102	0.0586
2 K	10 O	2.727122307	0.1037
2 K	11 O	2.779626067	0.0919
2 K	4 Cl	4.204948655	0.0806
3 K	6 Cl	3.514671045	0.0465
3 K	6 Cl	3.514671045	0.0433
3 K	9 O	2.945371806	0.0681
3 K	4 Cl	3.221347126	0.0806
3 K	4 Cl	4.434447492	0.0806
3 K	5 Cl	3.291918716	0.0636
3 K	6 Cl	3.486860414	0.0465
3 K	6 Cl	3.486860414	0.0433
3 K	7 Cl	3.263245042	0.0767
3 K	8 O	3.005371104	0.0586
3 K	10 O	2.779625774	0.0919
3 K	11 O	2.727122218	0.1037

Table S29: Full list of hydrogen interactions in the unit cell of Np(VI)-K

Hydrogen atom	H accepter	Interaction distance (Å)	Bond order
12 H	6 Cl	2.308288321	0.0962
13 H	7 Cl	2.308288398	0.0962
14 H	4 Cl	2.244477645	0.1166
14 H	4 Cl	4.462498262	0.1166
15 H	5 Cl	2.244477838	0.1166
15 H	5 Cl	4.462497979	0.1166

Table S30: Full list of cation interactions in the unit cell of Np(VI)-Rb

Metal ion	Cl or O atom	Interaction distance (Å)	Bond order
2 Rb	4 Cl	3.587223036	0.0597
2 Rb	4 Cl	3.587223036	0.0552
2 Rb	6 Cl	4.269620615	0.0922
2 Rb	10 O	2.873747669	0.111
2 Rb	4 Cl	3.556094213	0.0597
2 Rb	4 Cl	3.556094213	0.0552
2 Rb	5 Cl	3.402512243	0.0841
2 Rb	6 Cl	3.345602583	0.0922
2 Rb	6 Cl	4.495247578	0.0922
2 Rb	7 Cl	3.404181156	0.0763
2 Rb	8 O	3.057267784	0.0692
2 Rb	9 O	3.09880875	0.0611
2 Rb	11 O	2.947905662	0.0937
3 Rb	5 Cl	3.587222895	0.0552
3 Rb	5 Cl	3.587222895	0.0597
3 Rb	7 Cl	4.269620656	0.0922
3 Rb	11 O	2.87374738	0.111
3 Rb	4 Cl	3.402512248	0.0841
3 Rb	5 Cl	3.556094353	0.0552
3 Rb	5 Cl	3.556094353	0.0597
3 Rb	6 Cl	3.404181126	0.0763
3 Rb	7 Cl	3.34560258	0.0922
3 Rb	7 Cl	4.495247535	0.0922
3 Rb	8 O	3.098808829	0.0611
3 Rb	9 O	3.057267707	0.0692
3 Rb	10 O	2.947905635	0.0937

Table S31: Full list of hydrogen interactions in the unit cell of Np(VI)-Rb

Hydrogen atom	H accepter	Interaction distance (Å)	Bond order
12 H	7 Cl	2.2572847	0.1165
13 H	6 Cl	2.257285094	0.1165
14 H	5 Cl	2.317677225	0.0972
15 H	4 Cl	2.317677077	0.0972

Table S32 Full list of cation interactions in the unit cell of Np(VI)-Cs

Metal ion	Cl or O atom	Interaction distance (Å)	Bond order
3 Cs	10 Cl	3.544666	0.099
3 Cs	11 Cl	3.679676	0.0729
3 Cs	13 Cl	3.545682	0.0911
3 Cs	13 Cl	3.545682	0.0961
3 Cs	16 O	3.40762	0.0713
3 Cs	7 Cl	3.544666	0.099
3 Cs	12 Cl	3.545682	0.0911
3 Cs	12 Cl	3.545682	0.0961
3 Cs	12 Cl	3.572416	0.0911
3 Cs	12 Cl	3.572416	0.0961
3 Cs	13 Cl	3.572416	0.0911
3 Cs	13 Cl	3.572416	0.0961
3 Cs	14 Cl	3.679676	0.0729
3 Cs	16 O	4.452182	0.0713
4 Cs	8 Cl	3.544666	0.099
4 Cs	11 Cl	3.545682	0.0961
4 Cs	11 Cl	3.545682	0.0911
4 Cs	13 Cl	3.679676	0.0729
4 Cs	15 O	3.40762	0.0713
4 Cs	9 Cl	3.544666	0.099
4 Cs	11 Cl	3.572417	0.0961
4 Cs	11 Cl	3.572417	0.0911
4 Cs	12 Cl	3.679676	0.0729
4 Cs	14 Cl	3.545682	0.0961
4 Cs	14 Cl	3.545682	0.0911
4 Cs	14 Cl	3.572417	0.0961
4 Cs	14 Cl	3.572417	0.0911
4 Cs	15 O	4.452182	0.0713
5 Cs	8 Cl	3.545682	0.0911
5 Cs	8 Cl	3.545682	0.0961
5 Cs	9 Cl	3.545682	0.0911
5 Cs	9 Cl	3.545682	0.0961
5 Cs	11 Cl	3.544666	0.099
5 Cs	14 Cl	3.544666	0.099
5 Cs	18 O	3.40762	0.0713
5 Cs	7 Cl	3.679676	0.0729
5 Cs	8 Cl	3.572416	0.0911
5 Cs	8 Cl	3.572416	0.0961
5 Cs	9 Cl	3.572416	0.0911
5 Cs	9 Cl	3.572416	0.0961
5 Cs	10 Cl	3.679676	0.0729
5 Cs	18 O	4.452182	0.0713
6 Cs	7 Cl	3.545682	0.0961
6 Cs	7 Cl	3.545682	0.0911
6 Cs	10 Cl	3.545682	0.0961
6 Cs	10 Cl	3.545682	0.0911
6 Cs	12 Cl	3.544666	0.099
6 Cs	13 Cl	3.544666	0.099
6 Cs	17 O	3.40762	0.0713
6 Cs	7 Cl	3.572417	0.0961
6 Cs	7 Cl	3.572417	0.0911
6 Cs	8 Cl	3.679676	0.0729
6 Cs	9 Cl	3.679676	0.0729
6 Cs	10 Cl	3.572417	0.0961
6 Cs	10 Cl	3.572417	0.0911
6 Cs	17 O	4.452182	0.0713

7. Vibrational analysis

7.1 Raman spectroscopic analysis of neptunyl complexes.

SnRI High-Resolution Sierra 2.0 Raman spectrometer with 786 nm laser and 2048 pixels TE-CCD was used for the solid-state Raman investigation. The laser intensity was adjusted to 15 mW, and spectra were taken during time periods ranging from 2 to 60 seconds, depending on signal-to-noise ratios.

Peaks other than the $\nu_1$can be attributed to pure vibrational modes of organic cation or combination modes of neptunyl and organic cation vibrations. Additionally minor peaks in the ν_1 window are due to the presence of impurities in the solid phase. The selection of the correct neptunyl signal is aided by the computation vibrational analysis.

Model	PsdVoigt1																
Equatio	y = y0 + A * (mu * (2/pi) * (w / (4*(x-xc)^2 + w^2)) + (1 - mu) * (sqrt(4*ln(2)) / (sqrt(pi) * w)) * exp(-(4*ln(2)/w^2)*(x-xc)^2))																
Plot	Peak 1	Peak 2	Peak 3	Peak 4	Peak 5	Peak 6	Peak 7	Peak 8	Peak 9	Peak 10	Peak 11	Peak 12	Peak 13	Peak 14	Peak 15	Peak 16	Peak 17
y0	−4.3287	−4.3287	−4.3287	−4.3287	−4.3287	−4.3287	−4.3287	−4.3287	−4.3287	−4.3287	−4.32879	−4.32879	−4.32879	−4.3287	−4.3287	−4.3287	−4.3287
xc	620.248	741.280	755.567	768.018	795.122	806.741	845.020	906.159	1002.61	1033.55	1080.442	1094.748	1101.593	1156.65	1184.22	1198.33	1206.28
A	1216.40	274.876	448.993	362.855	1937.70	184.794	234.551	333.878	9360.87	2365.37	199.6631	129.5450	160.0021	872.520	1158.79	1624.08	776.541
w	7.15828	11.1546	12.6281	12.5 ± 0	15 ± 0	12 ± 0	15 ± 0	15 ± 0	6.7385	9.65607	13.58729	10.28521	11.14707	8.82569	10.4378	15.6753	12.9136
mu	1 ± 0	0.5 ± 0	0.5 ± 0	0.8 ± 0	1 ± 0	1 ± 0	1 ± 0	1 ± 0	1 ± 0	0.5 ± 0	1 ± 0	1 ± 0	1 ± 0	1 ± 0	1 ± 0	1 ± 0	1 ± 0
Reduce	67.46869																
R-Squa	0.98997																
Adj. R-	0.98981																

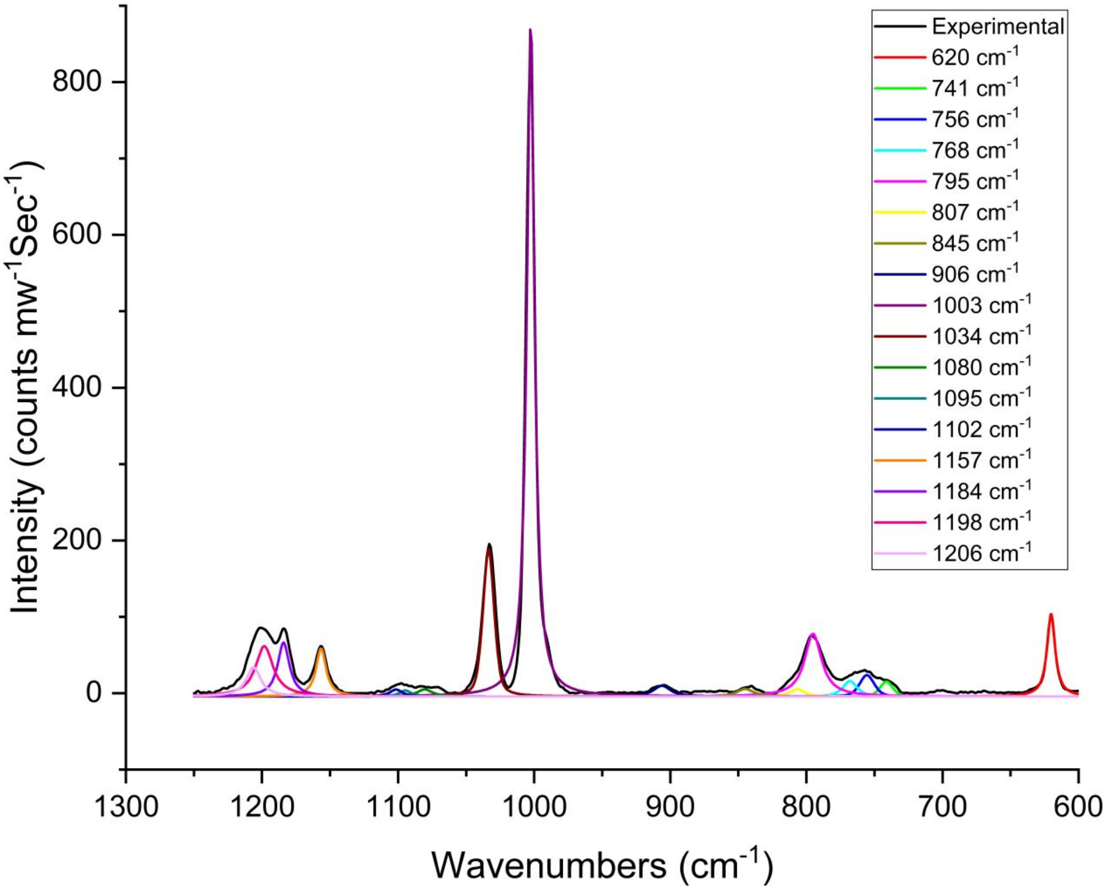

Figure S5: The fitted Raman spectrum of **Np(VI)-Bipy** in the spectral window 600-1200 cm^{-1}.

Model	PsdVoigt1				
Equation	y = y0 + A * (mu * (2/pi) * (w / (4*(x-xc)^2 + w^2)) + (1 - mu) * (sqrt(4*ln(2)) / (sqrt(pi) * w)) * exp(-(4*ln(2)/w^2)*(x-xc)^2))				
Plot	Peak 1	Peak 2	Peak 3	Peak 4	Peak 5
y0	−0.2519 ± 0.03606	−0.2519 ± 0.03606	−0.2519 ± 0.03606	−0.2519 ± 0.03606	−0.2519 ± 0.03606
xc	670.03063 ± 0.04046	804.35255 ± 0.0355	825.57401 ± 0.16728	844.28728 ± 0.5595	1009.55792 ± 0.03122
A	818.37389 ± 7.607	1566.86286 ± 13.99001	342.76212 ± 10.53589	42.6652 ± 3.55954	744.96777 ± 5.73358
w	13.59484 ± 0.12646	16.68464 ± 0.07924	14.26398 ± 0.52278	11 ± 0	8.08337 ± 0.07752
mu	0.58463 ± 0.02526	0.28329 ± 0.02083	0.7 ± 0	0.5 ± 0	0.71021 ± 0.02064
Reduced Chi-Sqr	0.38069				
R-Square (COD)	0.99826				
Adj. R-Square	0.99821				

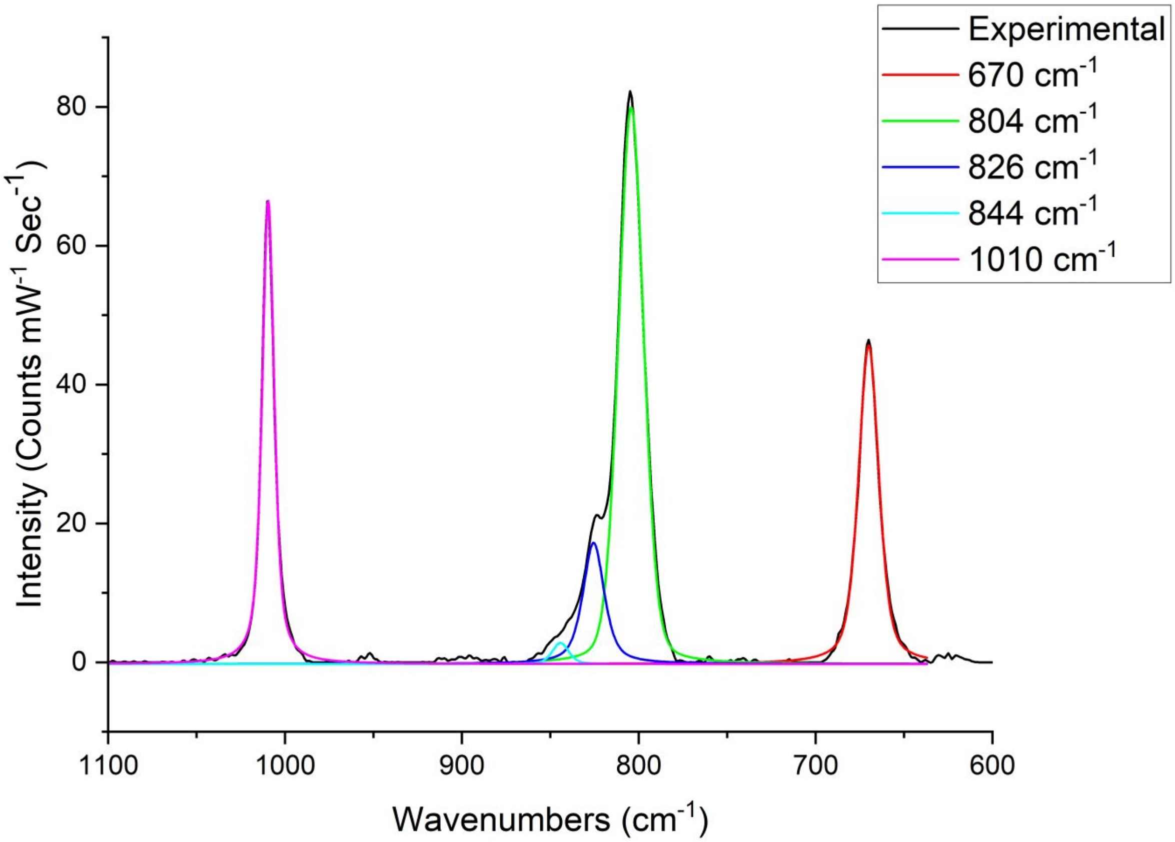

Figure S6: The fitted Raman spectrum of **Np(VI)-Gua** in the spectral window 600-1100 cm^{-1}.

Model	PsdVoigt1								
Equation	y = y0 + A * (mu * (2/pi) * (w / (4*(x-xc)^2 + w^2)) + (1 - mu) * (sqrt(4*ln(2)) / (sqrt(pi) * w)) * exp(-(4*ln(2)/w^2)*(x-xc)^2))								
Plot	Peak 1	Peak 2	Peak 3	Peak 4	Peak 5	Peak 6	Peak 7	Peak 8	Peak 9
y0	-0.10442 ± 0.0185	-0.10442 ± 0.0185	-0.10442 ± 0.0185	-0.10442 ± 0.0185	-0.10442 ± 0.01856	-0.10442 ± 0.01856	-0.10442 ± 0.01856	-0.10442 ± 0.01856	-0.10442 ± 0.01856
xc	803.1231 ± 0.2138	817.29957 ± 0.163	837.31906 ± 0.015	1017.8333 ± 0.130	1039.06512 ± 0.382	1070.16646 ± 0.270	1094.94309 ± 1.226	1169.85743 ± 2.135	1192.54884 ± 0.811
A	412.02935 ± 1.981	46.58595 ± 1.7150	742.11674 ± 1.948	83.8655 ± 1.93376	33.00193 ± 2.07795	42.54009 ± 1.98713	15.55692 ± 3.01962	6.97858 ± 0	17.82996 ± 2.45417
w	9.25604 ± 0.05771	5.22329 ± 0.30407	9.36346 ± 0.03271	6.88507 ± 0.24367	7.94832 ± 0.76763	7.00938 ± 0.49396	8.56903 ± 2.81934	6.45715 ± 3.16148	5.8484 ± 1.33482
mu	0.2 ± 0	0.5 ± 0	0.2 ± 0	0.5 ± 0	0.5 ± 0	0.5 ± 0	1 ± 0	1 ± 0	1 ± 0
Reduced Chi-S	0.14289								
R-Square (COD	0.9981								
Adj. R-Square	0.99802								

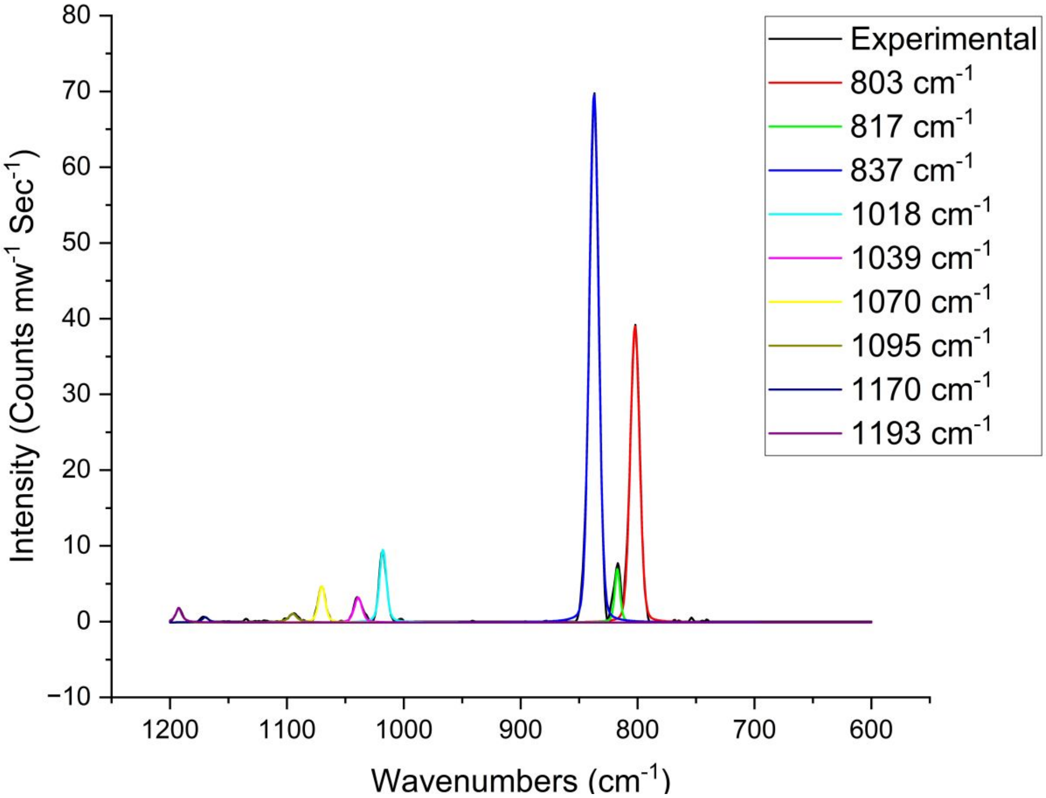

Figure S7: The fitted Raman spectrum of **Np(VI)-Morph** in the spectral window 600-1200 cm^{-1}.

Model	PsdVoigt1								
Equation	y = y0 + A * (mu * (2/pi) * (w / (4*(x-xc)^2 + w^2)) + (1 - mu) * (sqrt(4*ln(2)) / (sqrt(pi) * w)) * exp(-(4*ln(2)/w^2)*(x-xc)^2))								
Plot	Peak 1	Peak 2	Peak 3	Peak 4	Peak 5	Peak 6	Peak 7	Peak 8	Peak 9
y0	−0.31859 ± 0	−0.31859 ± 0	−0.31859 ± 0	−0.31859 ± 0.	−0.31859 ± 0	−0.31859 ± 0	−0.31859 ± 0	−0.31859 ± 0	−0.31859 ± 0
xc	622.35705 ±	642.63465 ±	698.99089 ±	718.90088 ±	725.04235 ±	736.25293 ±	799.69958 ±	838.92351 ±	849.58176 ±
A	127.7471 ± 3	125.72203 ±	21.49494 ± 3	182.02721 ±	69.78344 ± 6	71.64666 ± 1	822.00363 ±	561.85873 ±	290.12027 ±
w	13.54462 ± 0	10.5308 ± 0	11 ± 0	19.75431 ± 0	6.41157 ± 0	22.52172 ± 0	15.71435 ± 0	11.48607 ± 0	8.62859 ± 0
mu	0.2 ± 0	0.2 ± 0	0.2 ± 0	0.2 ± 0	0.2 ± 0	1 ± 0	0.06731 ± 0	1.07136 ± 0	0.5443 ± 0
Reduced C	0.38833								
R-Square (	0.99503								
Adj. R-Squ	0.99471								

Plot	Peak 10	Peak 11	Peak 12	Peak 13	Peak 14	Peak 15	Peak 16	Peak 17
y0	−0.31859 ± 0	−0.31859 ± 0.	−0.31859 ± 0.	−0.31859 ± 0	−0.31859 ± 0.	−0.31859 ±	−0.31859 ± 0.	−0.31859 ± 0.
xc	1000.1018 ±	1012.72881 ±	1046.83143 ±	1056.93886	1111.99192 ±	1200.77519	1233.27883 ±	1243.60584 ±
A	216.68989 ±	254.81597 ±	92.00449 ± 3	41.84603 ± 2	151.57829 ±	41.9434 ± 0	99.98589 ± 3	21.58621 ± 4
w	9.72896 ± 0	6.48249 ± 0	5.79465 ± 0	13.66768 ± 1	9.26853 ± 0.5	9.2168 ± 0	9.66204 ± 0.9	9.31753 ± 6.5
mu	0.10359 ± 0	0.1226 ± 0	1 ± 0	0.2 ± 0	1 ± 0	1.33657 ± 0	0.85908 ± 0.2	−0.28907 ± 4.

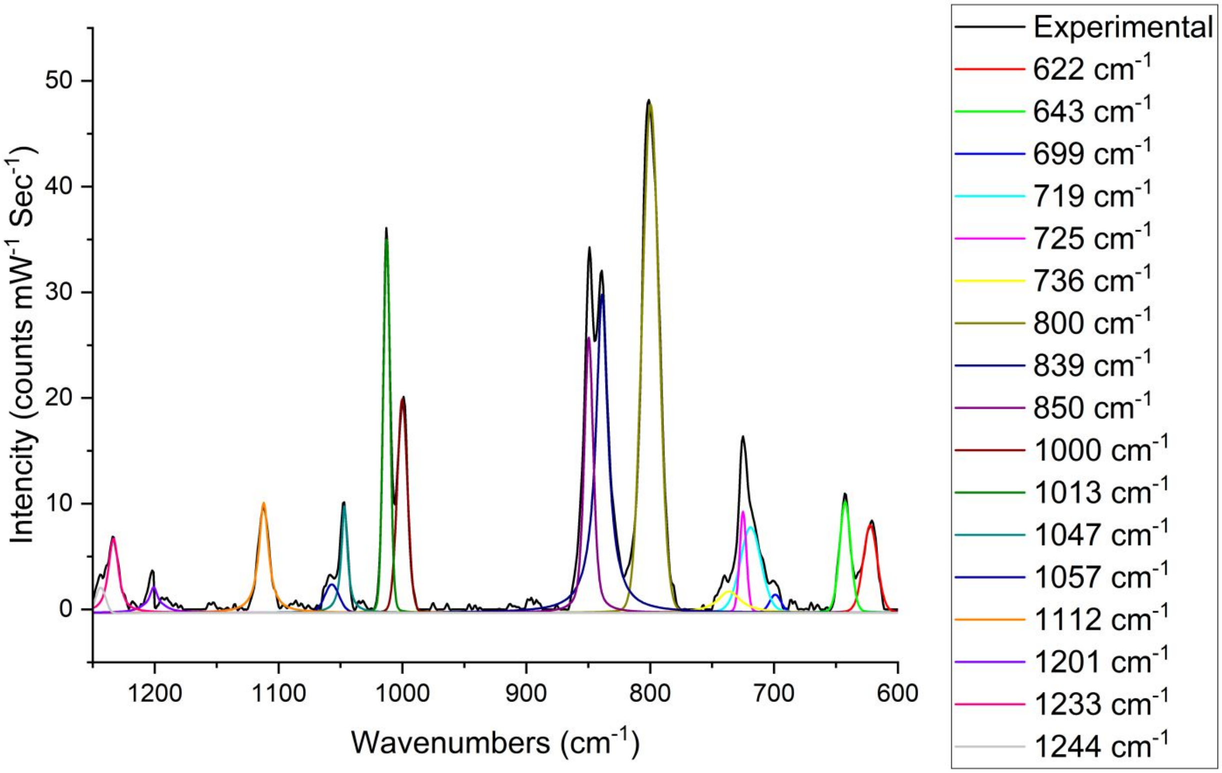

Figure S8: The fitted Raman spectrum of **Np(VI)-Apyr** in the spectral window 600-1200 cm^{-1}.

Model	PsdVoigt1									
Equation	y = y0 + A * (mu * (2/pi) * (w / (4*(x-xc)^2 + w^2)) + (1 - mu) * (sqrt(4*ln(2)) / (sqrt(pi) * w)) * exp(-(4*ln(2)/w^2)*(x-xc)^2))									
Plot	Peak 1	Peak 2	Peak 3	Peak 4	Peak 5	Peak 6	Peak 7	Peak 8	Peak 9	Peak 10
y0	-1.62351 ± 0.1379	-1.62351 ± 0.1379	-1.62351 ± 0.137	-1.62351 ± 0.137	-1.62351 ± 0.137	-1.62351 ± 0.137	-1.62351 ± 0.137	-1.62351 ± 0.137	-1.62351 ± 0.137	-1.62351 ± 0.1379
xc	806.03131 ± 0.060	819.90547 ± 0.299	906.18241 ± 1.03	921.20342 ± 1.5	1002.23419 ± 0.7	1045.41796 ± 2.7	1090.56199 ± 1.2	1102.82085 ± 0.7	1164.4608 ± 0.6	1191.00686 ± 0.05
A	3384.91859 ± 51.2	1369.50308 ± 72.4	163.46342 ± 26.3	66.62261 ± 16.6	124.93673 ± 16.9	65.08968 ± 23.21	158.65739 ± 42.3	329.52613 ± 53.2	448.61114 ± 34.	2683.74904 ± 24.2
w	12.99873 ± 0.1122	14.1838 ± 0.81238	13.00424 ± 2.750	10 ± 0	8.10083 ± 1.8107	13.95725 ± 7.414	8.11873 ± 1.5175	9.33026 ± 2.1461	19.58813 ± 1.89	9.82421 ± 0.13803
mu	0.5 ± 0	1 ± 0	1 ± 0	1 ± 0	1 ± 0	1 ± 0	1 ± 0	1 ± 0	1 ± 0	1 ± 0
Reduced Chi-	4.73911									
R-Square (CO	0.99588									
Adj. R-Square	0.99568									

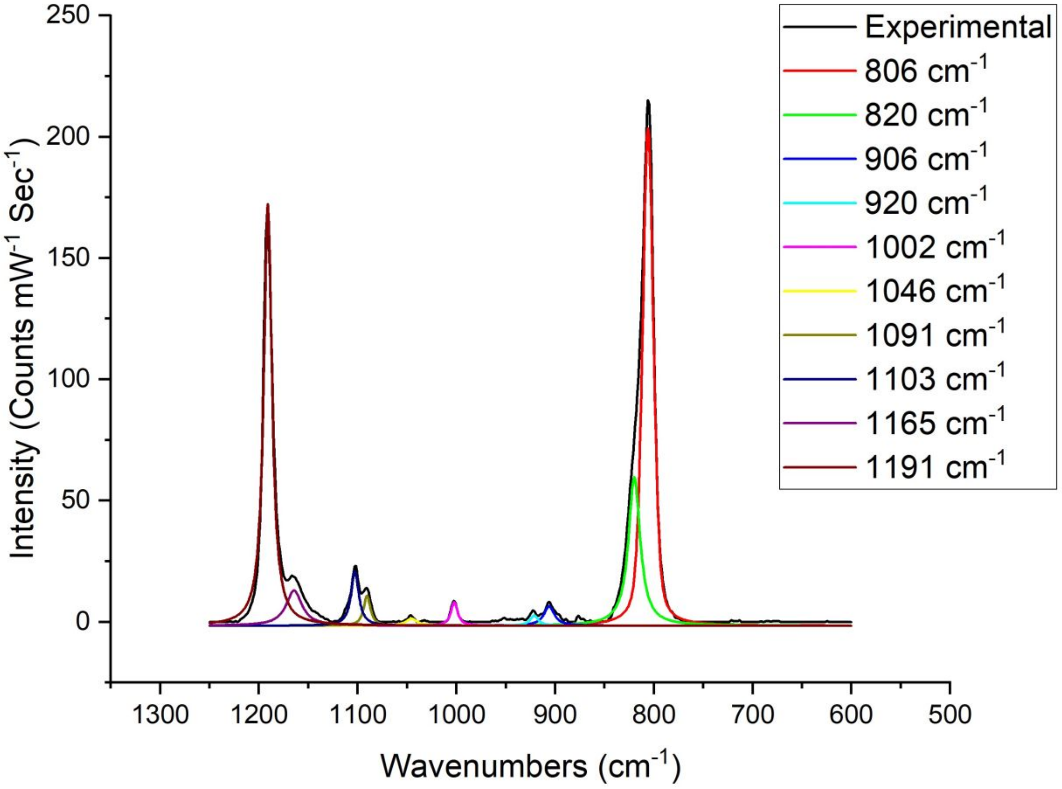

Figure S9: The fitted Raman spectrum of **Np(VI)-Imi** in the spectral window 600-1200 cm^{-1}.

Model	PsdVoigt1						
Equation	y = y0 + A * (mu * (2/pi) * (w / (4*(x-xc)^2 + w^2)) + (1 - mu) * (sqrt(4*ln(2)) / (sqrt(pi) * w)) * exp(-(4*ln(2)/w^2)*(x-xc)^2))						
Plot	Peak 1	Peak 2	Peak 3	Peak 4	Peak 5	Peak 6	Peak 7
y0	0.3708 ± 0.13447	0.3708 ± 0.13447	0.3708 ± 0.13447	0.3708 ± 0.13447	0.3708 ± 0.13447	0.3708 ± 0.13447	0.3708 ± 0.13447
xc	718.45785 ± 0.90417	791.3928 ± 0.019	805.76787 ± 0.04684	825.36717 ± 3.98747	1003.75943 ± 0.80778	1027.71143 ± 3.67501	1036.98328 ± 0.72492
A	157.97792 ± 19.20195	2640.50672 ± 10.58027	1026.61926 ± 10.34429	11.93358 ± 10.8508	149.97926 ± 21.50082	308.27463 ± 120.57741	193.08931 ± 95.54043
w	14.70189 ± 2.28268	6.3816 ± 0.03601	6.06235 ± 0.08827	5.06195 ± 7.07752	12.44016 ± 1.7207	19.50383 ± 4.43787	7.02578 ± 2.69895
mu	0.5 ± 0	0.2 ± 0	0.2 ± 0	0.5 ± 0	0.2 ± 0	1 ± 0	1 ± 0
Reduced Chi-Sqr	6.18146						
R-Square (COD)	0.99528						
Adj. R-Square	0.9951						

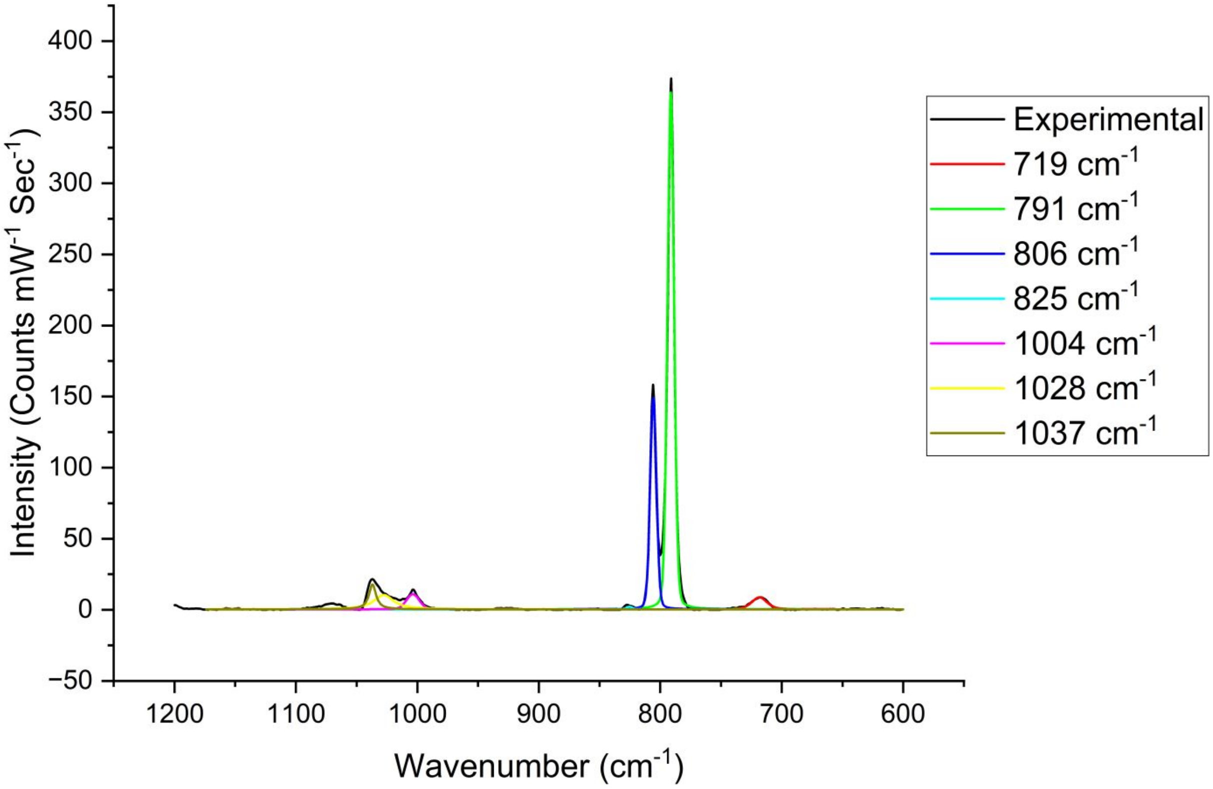

Figure S10: The fitted Raman spectrum of **Np(VI)-Pipz** in the spectral window 600-1200 cm^{-1}.

Model	PsdVoigt1					
Equation	y = y0 + A * (mu * (2/pi) * (w / (4*(x-xc)^2 + w^2)) + (1 - mu) * (sqrt(4*ln(2)) / (sqrt(pi) * w)) * exp(-(4*ln(2)/w^2)*(x-xc)^2))					
Plot	Peak 1	Peak 2	Peak 3	Peak 4	Peak 5	Peak 5
y0	0.09396 ± 0.06335	0.09396 ± 0.06335	0.09396 ± 0.06335	0.09396 ± 0.06335	0.09396 ± 0.06335	0.09396 ± 0.06335
xc	620.47667 ± 0.695	804.00563 ± 0.02619	1001.73664 ± 0.0837	1032.29077 ± 0.43034	1052.17913 ± 1.71092	1155.52276 ± 1.30157
A	53.0063 ± 8.77834	1495.90901 ± 7.92448	519.66026 ± 8.34651	109.07281 ± 8.69811	15.54516 ± 6.44774	60.73151 ± 8.71835
w	6.51623 ± 1.73087	6.93226 ± 0.0589	5.98383 ± 0.16517	6.33539 ± 0.86664	3.49569 ± 2.19727	12 ± 0
mu	1 ± 0	0.8 ± 0	1 ± 0	1 ± 0	1 ± 0	1 ± 0
Reduced Chi-Sqr	1.67941					
R-Square (COD)	0.99241					
Adj. R-Square	0.99219					

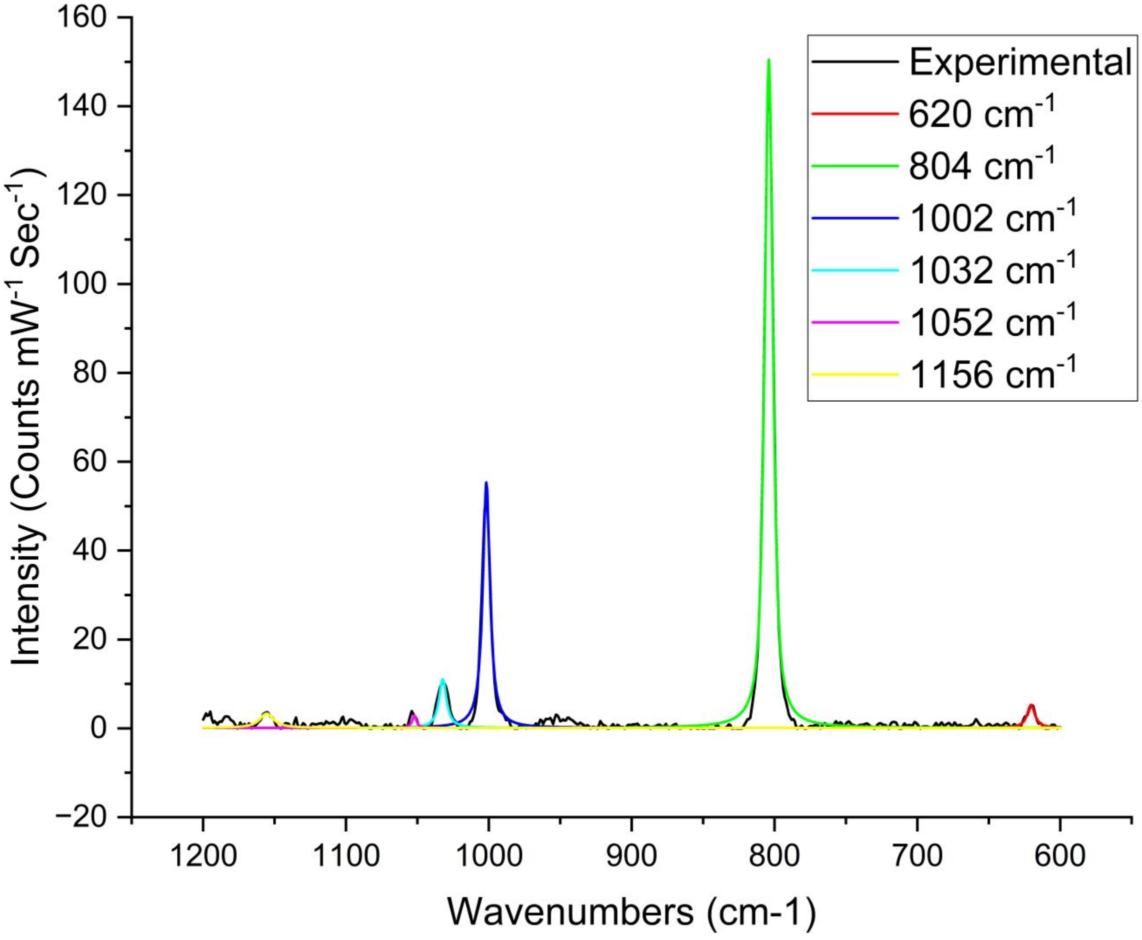

Figure S11: The fitted Raman spectrum of **Np(VI)-K** in the spectral window 600-1200 cm^{-1}.

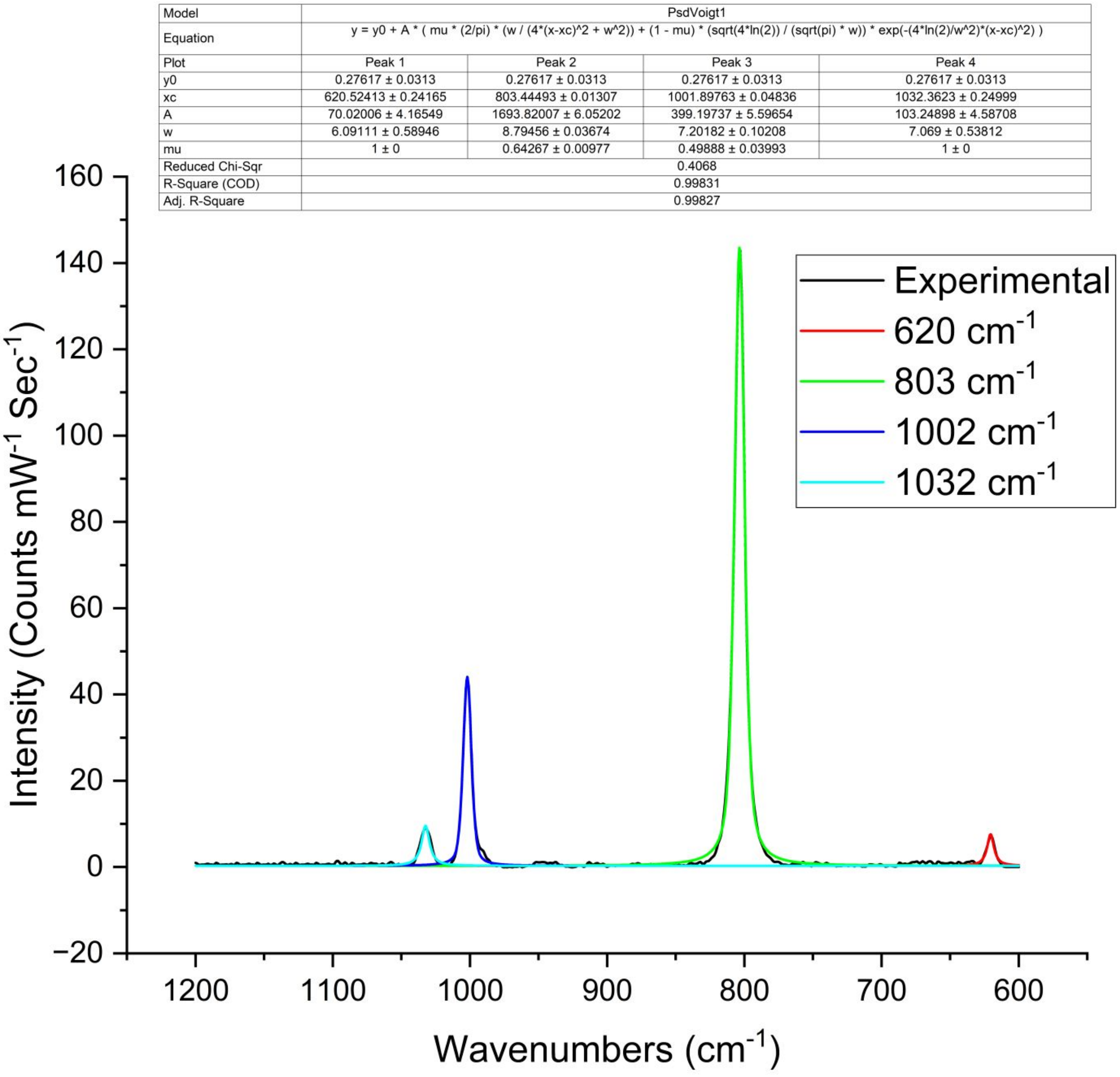

Model	PsdVoigt1			
Equation	y = y0 + A * (mu * (2/pi) * (w / (4*(x-xc)^2 + w^2)) + (1 - mu) * (sqrt(4*ln(2)) / (sqrt(pi) * w)) * exp(-(4*ln(2)/w^2)*(x-xc)^2))			
Plot	Peak 1	Peak 2	Peak 3	Peak 4
y0	0.27617 ± 0.0313	0.27617 ± 0.0313	0.27617 ± 0.0313	0.27617 ± 0.0313
xc	620.52413 ± 0.24165	803.44493 ± 0.01307	1001.89763 ± 0.04836	1032.3623 ± 0.24999
A	70.02006 ± 4.16549	1693.82007 ± 6.05202	399.19737 ± 5.59654	103.24898 ± 4.58708
w	6.09111 ± 0.58946	8.79456 ± 0.03674	7.20182 ± 0.10208	7.069 ± 0.53812
mu	1 ± 0	0.64267 ± 0.00977	0.49888 ± 0.03993	1 ± 0
Reduced Chi-Sqr	0.4068			
R-Square (COD)	0.99831			
Adj. R-Square	0.99827			

Figure S12: The fitted Raman spectrum of **Np(VI)-Rb** in the spectral window 600-1200 cm^{-1}.

7.2 Computation vibration analysis: Calculated phonon frequencies, neptunyl displacement amplitudes, and oxo displacement phase angle

The treatment of vibrational data was done according to the method illustrated by Spano *et al*[15] and in previous work we have successfully adopted this method to analyze uranyl vibrational modes.[6, 16]

$$S_{yl}^{n} = \sum_{i \in [yl]} |np_i^n| \qquad \text{Eq SI 2}$$

$$s^{n} = \frac{S_{yl}^{n}}{\sum_{i=1}^{N} |np_i^n|} \qquad \text{Eq SI 3}$$

$$\theta^{n} = \cos^{-1}\left(\frac{np_{oxo1}^{n} \cdot np_{oxo1}^{n}}{|np_{oxo1}^{n}| \cdot |np_{oxo1}^{n}|}\right) \qquad \text{Eq SI 4}$$

In Eq SI 2, $|np_i^n|$ is the displacement vectors of each atom of neptunyl cation ($NpO_2^{2+/+}$). S_{yl}^{n} is the total displacement amplitude of the neptunyl cation. The sum of the displacement amplitudes of all atoms (N) in the crystal structure is normalised by the total amplitude of the corresponding normal mode (Eq SI 3). Eq SI 4 thus represents the phase angle n between the displacement vectors of the two neptunyl oxo groups. We may use this approach to detect whether a particular normal mode is the consequence of symmetric ($\theta^n \approx 180°$) or asymmetric ($\theta^n \approx 0°$) stretching of the Np=O bonds.

Table S33: **Np(V)-Morph** phonon mode analysis in the spectral window of 600-1200 cm^{-1}. Red modes correspond to neptunyl symmetric (ν_1) and blue modes correspond to neptunyl asymmetric (ν_3) stretches.

	Np1		Np2		Np3		Np4	
**Energy (cm^{-1})**	s^n	θ^n	s^n	θ^n	s^n	θ^n	s^n	θ^n
668.9675	0.153904	177.2	0.153904	177.2	0.153904	177.2	0.153904	177.2
671.9044	0.154277	177.3	0.154277	177.3	0.154277	177.3	0.154277	177.3
672.2079	0.153962	177.3	0.153962	177.3	0.153962	177.3	0.153962	177.3
783.8249	0.129523	1.5	0.129523	1.5	0.129523	1.5	0.129523	1.5
783.9746	0.128455	1.5	0.128455	1.5	0.128455	1.5	0.128455	1.5
784.4598	0.128109	1.4	0.128109	1.4	0.128109	1.4	0.128109	1.4
785.9987	0.124509	1.3	0.124509	1.3	0.124509	1.3	0.124509	1.3
815.3887	0.001283	49.0	0.001283	49.0	0.001283	49.0	0.001283	49.0
815.4327	0.004903	5.6	0.004903	5.6	0.004903	5.6	0.004903	5.6
815.586	0.002175	23.5	0.002175	23.5	0.002175	23.5	0.002175	23.5
815.6747	0.004037	4.7	0.004037	4.7	0.004037	4.7	0.004037	4.7
817.7149	0.00921	5.5	0.00921	5.5	0.00921	5.5	0.00921	5.5
817.8881	0.01034	2.3	0.01034	2.3	0.01034	2.3	0.01034	2.3
818.266	0.011901	4.5	0.011901	4.5	0.011901	4.5	0.011901	4.5
818.3672	0.015432	3.2	0.015432	3.2	0.015432	3.2	0.015432	3.2
819.7487	0.001347	67.4	0.001347	67.4	0.001347	67.4	0.001347	67.4
819.773	0.002417	13.1	0.002417	13.1	0.002417	13.1	0.002417	13.1
820.3898	0.00225	28.9	0.00225	28.9	0.00225	28.9	0.00225	28.9
820.4193	0.004763	9.0	0.004763	9.0	0.004763	9.0	0.004763	9.0
820.8084	0.006177	15.8	0.006177	15.8	0.006177	15.8	0.006177	15.8
821.0283	0.006025	16.0	0.006025	16.0	0.006025	16.0	0.006025	16.0
821.6543	0.005948	15.9	0.005948	15.9	0.005948	15.9	0.005948	15.9
822.1493	0.008533	8.4	0.008533	8.4	0.008533	8.4	0.008533	8.4
822.556	0.000547	96.2	0.000547	96.2	0.000547	96.2	0.000547	96.2
822.617	0.002344	13.8	0.002344	13.8	0.002344	13.8	0.002344	13.8
823.4814	0.0012	10.2	0.0012	10.2	0.0012	10.2	0.0012	10.2
823.763	0.003284	3.5	0.003284	3.5	0.003284	3.5	0.003284	3.5
825.5629	0.007823	4.4	0.007823	4.4	0.007823	4.4	0.007823	4.4
825.586	0.007656	7.0	0.007656	7.0	0.007656	7.0	0.007656	7.0
825.7164	0.005814	1.9	0.005814	1.9	0.005814	1.9	0.005814	1.9
825.8949	0.008087	2.3	0.008087	2.3	0.008087	2.3	0.008087	2.3
858.34	0.002233	27.5	0.002233	27.5	0.002233	27.5	0.002233	27.5
858.7234	0.003059	13.4	0.003059	13.4	0.003059	13.4	0.003059	13.4
859.5178	0.003669	1.8	0.003669	1.8	0.003669	1.8	0.003669	1.8
859.5582	0.003098	12.4	0.003098	12.4	0.003098	12.4	0.003098	12.4
861.7265	0.001012	62.0	0.001012	62.0	0.001012	62.0	0.001012	62.0
861.7634	0.001769	133.6	0.001769	133.6	0.001769	133.6	0.001769	133.6
862.3304	0.00543	17.0	0.00543	17.0	0.00543	17.0	0.00543	17.0
862.3936	0.002884	17.5	0.002884	17.5	0.002884	17.5	0.002884	17.5
866.2646	0.004175	7.8	0.004175	7.8	0.004175	7.8	0.004175	7.8
866.3063	0.004202	13.1	0.004202	13.1	0.004202	13.1	0.004202	13.1
866.6737	0.003208	22.5	0.003208	22.5	0.003208	22.5	0.003208	22.5
866.9238	0.004373	21.3	0.004373	21.3	0.004373	21.3	0.004373	21.3
867.0595	0.002583	132.0	0.002583	132.0	0.002583	132.0	0.002583	132.0
867.0771	0.002019	117.7	0.002019	117.7	0.002019	117.7	0.002019	117.7
867.2183	0.002695	140.9	0.002695	140.9	0.002695	140.9	0.002695	140.9
867.9188	0.002242	142.2	0.002242	142.2	0.002242	142.2	0.002242	142.2

875.8702	0.000986	138.9	0.000986	138.9	0.000986	138.9	0.000986	138.9
875.971	0.002453	4.9	0.002453	4.9	0.002453	4.9	0.002453	4.9
877.5383	0.001528	14.4	0.001528	14.4	0.001528	14.4	0.001528	14.4
877.6777	0.002789	17.7	0.002789	17.7	0.002789	17.7	0.002789	17.7
878.4408	0.002164	20.6	0.002164	20.6	0.002164	20.6	0.002164	20.6
878.6402	0.002816	15.6	0.002816	15.6	0.002816	15.6	0.002816	15.6
879.7068	0.002721	4.6	0.002721	4.6	0.002721	4.6	0.002721	4.6
879.8145	0.00112	136.2	0.00112	136.2	0.00112	136.2	0.00112	136.2
880.413	0.001162	28.6	0.001162	28.6	0.001162	28.6	0.001162	28.6
881.0513	0.000827	158.9	0.000827	158.9	0.000827	158.9	0.000827	158.9
884.1771	0.000771	120.6	0.000771	120.6	0.000771	120.6	0.000771	120.6
884.282	0.001374	133.7	0.001374	133.7	0.001374	133.7	0.001374	133.7
902.5622	0.01574	3.6	0.01574	3.6	0.01574	3.6	0.01574	3.6
902.8509	0.01415	6.4	0.01415	6.4	0.01415	6.4	0.01415	6.4
903.3258	0.015795	4.6	0.015795	4.6	0.015795	4.6	0.015795	4.6
903.9401	0.015626	4.2	0.015626	4.2	0.015626	4.2	0.015626	4.2
921.0028	0.002526	122.5	0.002526	122.5	0.002526	122.5	0.002526	122.5
921.046	0.002475	112.7	0.002475	112.7	0.002475	112.7	0.002475	112.7
921.2043	0.002253	102.7	0.002253	102.7	0.002253	102.7	0.002253	102.7
921.5244	0.00242	117.7	0.00242	117.7	0.00242	117.7	0.00242	117.7
998.5325	0.002713	18.8	0.002713	18.8	0.002713	18.8	0.002713	18.8
998.7092	0.003321	12.4	0.003321	12.4	0.003321	12.4	0.003321	12.4
1001.904	0.002527	20.0	0.002527	20.0	0.002527	20.0	0.002527	20.0
1001.977	0.003052	13.2	0.003052	13.2	0.003052	13.2	0.003052	13.2
1008.456	0.001909	25.0	0.001909	25.0	0.001909	25.0	0.001909	25.0
1008.486	0.001516	88.3	0.001516	88.3	0.001516	88.3	0.001516	88.3
1009.074	0.002142	18.6	0.002142	18.6	0.002142	18.6	0.002142	18.6
1009.078	0.001328	93.2	0.001328	93.2	0.001328	93.2	0.001328	93.2
1010.431	0.000941	137.3	0.000941	137.3	0.000941	137.3	0.000941	137.3
1010.699	0.001647	7.9	0.001647	7.9	0.001647	7.9	0.001647	7.9
1010.842	0.000985	105.1	0.000985	105.1	0.000985	105.1	0.000985	105.1
1010.852	0.001361	172.2	0.001361	172.2	0.001361	172.2	0.001361	172.2
1010.876	0.001169	165.2	0.001169	165.2	0.001169	165.2	0.001169	165.2
1010.972	0.000918	11.6	0.000918	11.6	0.000918	11.6	0.000918	11.6
1016.568	0.001537	46.4	0.001537	46.4	0.001537	46.4	0.001537	46.4
1016.677	0.001331	10.3	0.001331	10.3	0.001331	10.3	0.001331	10.3
1019.007	0.001668	22.6	0.001668	22.6	0.001668	22.6	0.001668	22.6
1019.674	0.001386	26.3	0.001386	26.3	0.001386	26.3	0.001386	26.3
1020.423	0.000962	63.0	0.000962	63.0	0.000962	63.0	0.000962	63.0
1020.527	0.000384	114.5	0.000384	114.5	0.000384	114.5	0.000384	114.5
1024.444	0.000989	64.3	0.000989	64.3	0.000989	64.3	0.000989	64.3
1025.331	0.000469	142.8	0.000469	142.8	0.000469	142.8	0.000469	142.8
1026.446	0.001478	11.0	0.001478	11.0	0.001478	11.0	0.001478	11.0
1026.501	0.000854	18.1	0.000854	18.1	0.000854	18.1	0.000854	18.1
1026.593	0.000718	135.9	0.000718	135.9	0.000718	135.9	0.000718	135.9
1026.798	0.000299	148.1	0.000299	148.1	0.000299	148.1	0.000299	148.1
1028.08	0.001288	22.2	0.001288	22.2	0.001288	22.2	0.001288	22.2
1028.116	0.001268	23.4	0.001268	23.4	0.001268	23.4	0.001268	23.4
1028.813	0.000659	90.3	0.000659	90.3	0.000659	90.3	0.000659	90.3
1029.197	0.002096	14.9	0.002096	14.9	0.002096	14.9	0.002096	14.9
1029.395	0.002206	17.6	0.002206	17.6	0.002206	17.6	0.002206	17.6
1029.477	0.000707	96.5	0.000707	96.5	0.000707	96.5	0.000707	96.5
1032.009	0.001245	36.6	0.001245	36.6	0.001245	36.6	0.001245	36.6
1032.172	0.000863	53.4	0.000863	53.4	0.000863	53.4	0.000863	53.4

1032.183	0.001107	33.2	0.001107	33.2	0.001107	33.2	0.001107	33.2
1032.363	0.000818	42.6	0.000818	42.6	0.000818	42.6	0.000818	42.6
1065.347	0.002079	12.4	0.002079	12.4	0.002079	12.4	0.002079	12.4
1065.528	0.002467	4.7	0.002467	4.7	0.002467	4.7	0.002467	4.7
1066.044	0.00149	20.3	0.00149	20.3	0.00149	20.3	0.00149	20.3
1066.265	0.002167	6.3	0.002167	6.3	0.002167	6.3	0.002167	6.3
1067.107	0.000464	48.8	0.000464	48.8	0.000464	48.8	0.000464	48.8
1067.143	0.001143	14.6	0.001143	14.6	0.001143	14.6	0.001143	14.6
1068.68	0.000592	39.6	0.000592	39.6	0.000592	39.6	0.000592	39.6
1068.687	0.000436	73.2	0.000436	73.2	0.000436	73.2	0.000436	73.2
1070.572	0.001286	19.4	0.001286	19.4	0.001286	19.4	0.001286	19.4
1070.663	0.001117	55.9	0.001117	55.9	0.001117	55.9	0.001117	55.9
1070.788	0.001531	156.9	0.001531	156.9	0.001531	156.9	0.001531	156.9
1070.833	0.001394	156.7	0.001394	156.7	0.001394	156.7	0.001394	156.7
1077.459	0.000926	50.9	0.000926	50.9	0.000926	50.9	0.000926	50.9
1077.951	0.000682	99.7	0.000682	99.7	0.000682	99.7	0.000682	99.7
1078.818	0.000543	105.6	0.000543	105.6	0.000543	105.6	0.000543	105.6
1079.226	0.000636	112.8	0.000636	112.8	0.000636	112.8	0.000636	112.8
1080.554	0.000585	131.6	0.000585	131.6	0.000585	131.6	0.000585	131.6
1081.351	0.001181	27.6	0.001181	27.6	0.001181	27.6	0.001181	27.6
1081.612	0.000623	83.7	0.000623	83.7	0.000623	83.7	0.000623	83.7
1082.176	0.000773	34.8	0.000773	34.8	0.000773	34.8	0.000773	34.8
1083.673	0.001354	164.9	0.001354	164.9	0.001354	164.9	0.001354	164.9
1083.809	0.001332	154.9	0.001332	154.9	0.001332	154.9	0.001332	154.9
1085.39	0.000924	137.8	0.000924	137.8	0.000924	137.8	0.000924	137.8
1085.889	0.001013	110.2	0.001013	110.2	0.001013	110.2	0.001013	110.2
1143.533	0.00056	104.9	0.00056	104.9	0.00056	104.9	0.00056	104.9
1144.019	0.000604	84.5	0.000604	84.5	0.000604	84.5	0.000604	84.5
1144.103	0.000487	91.4	0.000487	91.4	0.000487	91.4	0.000487	91.4
1144.142	0.000466	110.7	0.000466	110.7	0.000466	110.7	0.000466	110.7
1158.665	0.001465	13.0	0.001465	13.0	0.001465	13.0	0.001465	13.0
1159.119	0.001348	32.9	0.001348	32.9	0.001348	32.9	0.001348	32.9
1159.715	0.001249	62.8	0.001249	62.8	0.001249	62.8	0.001249	62.8
1159.733	0.001364	36.0	0.001364	36.0	0.001364	36.0	0.001364	36.0
1163.489	0.00104	33.9	0.00104	33.9	0.00104	33.9	0.00104	33.9
1163.603	0.000779	46.8	0.000779	46.8	0.000779	46.8	0.000779	46.8
1163.63	0.000953	21.1	0.000953	21.1	0.000953	21.1	0.000953	21.1
1164.211	0.000646	6.5	0.000646	6.5	0.000646	6.5	0.000646	6.5
1166.356	0.001082	38.1	0.001082	38.1	0.001082	38.1	0.001082	38.1
1166.451	0.001134	33.5	0.001134	33.5	0.001134	33.5	0.001134	33.5
1167.678	0.00115	28.3	0.00115	28.3	0.00115	28.3	0.00115	28.3
1167.706	0.001261	28.1	0.001261	28.1	0.001261	28.1	0.001261	28.1
1173.336	0.000699	11.9	0.000699	11.9	0.000699	11.9	0.000699	11.9
1173.39	0.000606	52.5	0.000606	52.5	0.000606	52.5	0.000606	52.5
1173.577	0.000568	40.3	0.000568	40.3	0.000568	40.3	0.000568	40.3
1173.584	0.000732	6.9	0.000732	6.9	0.000732	6.9	0.000732	6.9
1186.681	0.000537	58.4	0.000537	58.4	0.000537	58.4	0.000537	58.4
1186.73	0.000639	88.7	0.000639	88.7	0.000639	88.7	0.000639	88.7
1186.923	0.000436	83.7	0.000436	83.7	0.000436	83.7	0.000436	83.7
1187.027	0.000741	50.8	0.000741	50.8	0.000741	50.8	0.000741	50.8

Table S34: **Np(V)-Pipz** phonon mode analysis in the spectral window of 600-1200 cm^{-1} Red modes correspond to neptunyl symmetric (ν1) and blue modes correspond to neptunyl asymmetric (ν3) stretches.

	Np1		Np2	
Energy (cm^{-1})	s^n	θ^n	s^n	θ^n
647.7236092	0.22781255	180	0.315111	180
649.2905957	0.22655202	0	0.32307	0
767.6383377	0.32567667	0	0.22812	0
768.0807683	0.29028718	180	0.207986	180
807.264284	0.01376742	180	0.009794	180
807.6888879	0.00940382	0	0.007874	0
809.9679606	0.02477958	180	0.020222	180
811.6061794	0.00932032	180	0.006221	180
813.3383508	0.01615011	180	0.006969	180
814.5926901	0.00759644	180	0.008473	180
814.6795892	0.0089881	0	0.011155	0
826.6390388	0.03581332	180	0.017845	180
844.7965501	0.01532536	180	0.027568	180
846.3920119	0.01678828	0	0.026473	0
862.5656272	0.00320155	0	0.004712	0
863.7771902	0.03229588	180	0.00947	180
864.2478039	0.00365427	0	0.001966	0
864.7717947	0.0107172	180	0.002619	180
865.0339293	0.01754491	0	0.002597	0
870.7402824	0.00088555	0	0.003102	0
871.4306726	0.00620942	180	0.013447	180
873.5331944	0.01637304	0	0.019881	0
879.2137147	0.01742995	0	0.006777	0
896.1017157	0.01798149	180	0.022574	180
923.2948988	0.00533327	0	0.019659	0
923.5535415	0.01019687	180	0.024591	180
946.3180378	0.01469397	0	0.008719	0
952.9802426	0.01561093	0	0.004434	0
984.9532227	0.00055314	0	0.000455	0
987.4672352	0.00037482	180	0.002033	180
995.2095346	0.00463426	0	0.00151	0
999.6862269	0.00097118	0	0.004155	0
1026.040887	0.00169578	180	0.002795	180
1030.028953	0.00107208	0	0.004309	0
1030.323636	0.00144745	180	0.003833	180
1036.612228	0.01153733	180	0.003061	180
1042.26912	0.0007558	0	0.002035	0
1043.082841	0.00143251	180	0.00364	180
1043.950001	0.00165518	180	0.009348	180
1050.733387	0.00099027	180	0.005363	180
1052.242322	0.00253034	0	0.00366	0
1054.686352	0.00361984	0	0.001584	0
1062.490054	0.01092976	0	0.018199	0
1066.669204	0.00440588	180	0.003646	180
1073.935249	0.00128628	0	0.001351	0
1082.678403	0.00108892	0	0.000294	0
1082.886183	0.00044867	180	0.000511	180

1086.645162	0.00224964	0	0.002189	0
1130.756822	0.00041736	180	0.000871	180
1132.529482	0.00156277	0	0.00164	0
1137.183528	0.00165925	180	0.003339	180
1151.35391	0.00292796	180	0.005535	180
1167.259917	0.00239633	180	0.004995	180
1170.362948	0.00299809	0	0.003437	0
1188.160064	0.00099228	180	0.002879	180
1189.860596	0.00126819	0	0.008232	0
1189.945354	0.00229388	0	0.003524	0
1195.108168	0.00205691	180	0.003723	180
1200.780175	0.00184132	180	0.004954	180

Table S35: **Np(V)-Cs** phonon mode analysis in the spectral window of 600-1200 cm^{-1}. Red modes correspond to neptunyl symmetric (ν1) and blue modes correspond to neptunyl asymmetric (ν3) stretches.

	Np1		Np2		Np3		Np4	
**Energy (cm^{-1})**	**s^n**	**θ^n**	**s^n**	**θ^n**	**s^n**	**θ^n**	**s^n**	**θ^n**
682.9278	0.224345	180	0.224345	180	0.224345	180	0.224345	180
683.9821	0.224264	180	0.224264	180	0.224264	180	0.224264	180
686.5906	0.223927	180	0.223927	180	0.223927	180	0.223927	180
687.4702	0.222527	180	0.222527	180	0.222527	180	0.222527	180
822.4879	0.245409	0	0.245409	0	0.245409	0	0.245409	0
831.6578	0.242265	0	0.242265	0	0.242265	0	0.242265	0
832.488	0.242035	0	0.242035	0	0.242035	0	0.242035	0
839.5312	0.240643	0	0.240643	0	0.240643	0	0.240643	0

Table S36: **Np(VI)-Bipy** phonon mode analysis in the spectral window of 600-1200 cm^{-1}. Red modes correspond to neptunyl symmetric (ν_1) and blue modes correspond to neptunyl asymmetric (ν_3) stretches.

	Np1		Np2		Np3		Np4	
**Energy (cm^{-1})**	**s^n**	**θ^n**	**s^n**	**θ^n**	**s^n**	**θ^n**	**s^n**	**θ^n**
614.4025	0.000702	0	0.000702	0	0.000934	0	0.000934	0
614.4537	0.0015	0	0.0015	0	0.001094	0	0.001094	0
614.5543	0.000368	180	0.000368	180	0.000392	180	0.000392	180
614.831	0.001252	180	0.001252	180	0.00032	180	0.00032	180
617.6379	0.001299	0	0.001299	0	0.001099	0	0.001099	0
618.0716	0.002066	180	0.002066	180	0.001462	180	0.001462	180
618.8028	0.002873	180	0.002873	180	0.001353	180	0.001353	180
619.7751	0.002867	0	0.002867	0	0.001244	0	0.001244	0
635.1736	0.000796	0	0.000796	0	0.001442	0	0.001442	0
635.4773	0.003266	180	0.003266	180	0.00101	180	0.00101	180
635.6376	0.002706	0	0.002706	0	0.00152	0	0.00152	0
635.7637	0.000465	180	0.000465	180	0.001052	180	0.001052	180
712.6584	0.000342	0	0.000342	0	0.001077	0	0.001077	0
712.78	0.000785	180	0.000785	180	0.002098	180	0.002098	180
713.131	0.000608	0	0.000608	0	0.000967	0	0.000967	0

713.2738	0.000407	180	0.000407	180	0.001421	180	0.001421	180
729.4097	0.002166	0	0.002166	0	0.001927	0	0.001927	0
729.9039	0.000207	180	0.000207	180	0.003427	180	0.003427	180
730.8762	0.006759	180	0.006759	180	0.001135	180	0.001135	180
731.6308	0.001833	0	0.001833	0	0.001324	0	0.001324	0
745.6474	0.001874	0	0.001874	0	0.002038	0	0.002038	0
745.7925	0.012482	180	0.012482	180	0.002522	180	0.002522	180
752.8508	0.003245	0	0.003245	0	0.002173	0	0.002173	0
754.4606	0.009341	180	0.009341	180	0.008959	180	0.008959	180
759.7985	0.001768	180	0.001768	180	0.005605	180	0.005605	180
759.9894	0.002651	0	0.002651	0	0.002076	0	0.002076	0
760.3631	0.00688	180	0.00688	180	0.005175	180	0.005175	180
760.6649	0.001873	0	0.001873	0	0.003172	0	0.003172	0
784.8374	0.004559	0	0.004559	0	0.004609	0	0.004609	0
786.2969	0.024821	180	0.024821	180	0.013418	180	0.013418	180
787.5666	0.047684	180	0.047684	180	0.016099	180	0.016099	180
789.8831	0.001823	0	0.001823	0	0.003776	0	0.003776	0
794.187	0.235022	180	0.235022	180	0.021459	180	0.021459	180
795.4115	0.164748	180	0.164748	180	0.002496	180	0.002496	180
825.267	0.012195	180	0.012195	180	0.259332	180	0.259332	180
827.9252	0.017467	180	0.017467	180	0.278589	180	0.278589	180
859.9967	0.00447	0	0.00447	0	0.001478	0	0.001478	0
860.0857	0.003745	180	0.003745	180	0.001092	180	0.001092	180
860.4934	0.001846	180	0.001846	180	0.013976	180	0.013976	180
861.0045	0.002334	0	0.002334	0	0.003825	0	0.003825	0
871.9162	0.016128	0	0.016128	0	0.002331	0	0.002331	0
875.2741	0.011147	0	0.011147	0	0.002158	0	0.002158	0
876.379	0.012919	180	0.012919	180	0.006215	180	0.006215	180
880.4794	0.004857	180	0.004857	180	0.013544	180	0.013544	180
917.6343	0.017255	0	0.017255	0	0.008708	0	0.008708	0
919.689	0.001799	180	0.001799	180	0.002329	180	0.002329	180
920.8279	0.013885	0	0.013885	0	0.009899	0	0.009899	0
923.2141	0.000989	180	0.000989	180	0.00107	180	0.00107	180
933.7743	0.028909	0	0.028909	0	0.009543	0	0.009543	0
936.6827	0.028275	0	0.028275	0	0.006019	0	0.006019	0
937.9812	0.007149	180	0.007149	180	0.001886	180	0.001886	180
938.031	0.00525	180	0.00525	180	0.001044	180	0.001044	180
974.1162	0.186909	0	0.186909	0	0.005905	0	0.005905	0
975.2497	0.146805	0	0.146805	0	0.006156	0	0.006156	0
982.7968	0.037832	0	0.037832	0	0.011363	0	0.011363	0
983.104	0.000555	180	0.000555	180	0.00102	180	0.00102	180
983.4463	0.002402	180	0.002402	180	0.00139	180	0.00139	180
983.5356	0.004394	0	0.004394	0	0.006197	0	0.006197	0
988.5816	0.036047	0	0.036047	0	0.000811	0	0.000811	0
990.4241	0.003177	180	0.003177	180	0.001771	180	0.001771	180

990.5035	0.001429	180	0.001429	180	0.00141	180	0.00141	180
990.7144	0.069722	0	0.069722	0	0.004066	0	0.004066	0
1001.856	0.002139	0	0.002139	0	0.066208	0	0.066208	0
1002.613	0.010933	0	0.010933	0	0.063002	0	0.063002	0
1003.753	0.002207	180	0.002207	180	0.00039	180	0.00039	180
1003.93	0.001748	180	0.001748	180	0.002132	180	0.002132	180
1006.283	0.013387	0	0.013387	0	0.083385	0	0.083385	0
1006.562	0.009381	0	0.009381	0	0.079915	0	0.079915	0
1009.85	0.011834	0	0.011834	0	0.013388	0	0.013388	0
1009.949	0.00045	180	0.00045	180	0.00065	180	0.00065	180
1010.514	0.003364	180	0.003364	180	0.000562	180	0.000562	180
1011.051	0.000251	180	0.000251	180	0.002453	180	0.002453	180
1011.91	0.016282	0	0.016282	0	0.034015	0	0.034015	0
1012.158	0.000827	180	0.000827	180	0.002379	180	0.002379	180
1012.515	0.023728	0	0.023728	0	0.021832	0	0.021832	0
1013.548	0.000831	0	0.000831	0	0.011727	0	0.011727	0
1013.872	0.000866	180	0.000866	180	0.002661	180	0.002661	180
1015.335	0.000678	180	0.000678	180	0.002533	180	0.002533	180
1017.872	0.011335	0	0.011335	0	0.116485	0	0.116485	0
1019.279	0.000622	0	0.000622	0	0.098263	0	0.098263	0
1036.503	0.00246	0	0.00246	0	0.016119	0	0.016119	0
1036.766	0.001171	180	0.001171	180	0.000744	180	0.000744	180
1037.319	0.000645	180	0.000645	180	0.00173	180	0.00173	180
1037.982	0.008486	0	0.008486	0	0.015774	0	0.015774	0
1051.211	0.001141	180	0.001141	180	0.000297	180	0.000297	180
1051.437	0.011996	0	0.011996	0	0.001222	0	0.001222	0
1051.711	0.001982	180	0.001982	180	0.000257	180	0.000257	180
1051.771	0.006015	0	0.006015	0	0.003473	0	0.003473	0
1075.13	0.000561	180	0.000561	180	4.75E-05	180	4.75E-05	180
1075.151	0.000651	180	0.000651	180	4.24E-05	180	4.24E-05	180
1075.184	0.00175	0	0.00175	0	0.001223	0	0.001223	0
1075.298	0.004327	0	0.004327	0	0.000401	0	0.000401	0
1100.1	0.000453	180	0.000453	180	0.000899	180	0.000899	180
1100.53	0.001676	0	0.001676	0	0.002456	0	0.002456	0
1100.544	0.004674	0	0.004674	0	0.004158	0	0.004158	0
1100.58	0.001302	180	0.001302	180	0.000265	180	0.000265	180
1104.571	0.001428	180	0.001428	180	0.00117	180	0.00117	180
1105.027	0.004804	0	0.004804	0	0.004141	0	0.004141	0
1105.081	0.002558	0	0.002558	0	0.005392	0	0.005392	0
1105.29	0.001387	180	0.001387	180	0.000244	180	0.000244	180
1158.71	0.001289	180	0.001289	180	0.001349	180	0.001349	180
1158.86	0.001158	0	0.001158	0	0.007319	0	0.007319	0
1158.975	0.001108	180	0.001108	180	0.001	180	0.001	180
1160.228	0.001481	0	0.001481	0	0.00537	0	0.00537	0
1164.61	0.001451	0	0.001451	0	0.000946	0	0.000946	0

1165.226	0.004138	0	0.004138	0	0.001006	0	0.001006	0
1165.524	0.000216	180	0.000216	180	0.000244	180	0.000244	180
1166.244	0.000492	180	0.000492	180	0.000281	180	0.000281	180

Table S37: **Np(VI)-Gua** phonon mode analysis in the spectral window of 600-1200 cm^{-1}. Red modes correspond to uranyl symmetric (ν_1) and asymmetric (ν_3) stretches.

	Np1		Np2	
**Energy (cm^{-1})**	**s^n**	**θ^n**	**s^n**	**θ^n**
609.8099	0.003122	180	0.003122	180
616.5791	0.00883	0	0.00883	0
625.6093	0.006416	180	0.006416	180
626.3478	0.012715	180	0.012715	180
630.265	0.00454	180	0.00454	180
652.9099	0.007578	0	0.007578	0
657.465	0.007232	0	0.007232	0
702.3015	0.001454	180	0.001454	180
703.4157	0.003478	0	0.003478	0
705.192	0.001057	180	0.001057	180
705.3972	0.003411	0	0.003411	0
813.8983	0.378651	180	0.378651	180
820.5465	0.380396	180	0.380396	180
990.1089	0.209596	0	0.209596	0
992.4781	0.234522	0	0.234522	0
1005.591	0.046821	0	0.046821	0
1005.651	0.032131	0	0.032131	0
1006.882	0.000819	180	0.000819	180
1007.454	0.005511	180	0.005511	180
1016.463	0.008503	180	0.008503	180
1017.632	0.007366	180	0.007366	180
1020.509	0.085693	0	0.085693	0
1026.16	0.064493	0	0.064493	0
1083.859	0.00387	180	0.00387	180
1084.72	0.00404	180	0.00404	180
1085.421	0.026416	0	0.026416	0
1086.516	0.025319	0	0.025319	0
1125.128	0.001158	180	0.001158	180
1125.717	0.004443	0	0.004443	0
1127.466	0.010081	0	0.010081	0
1129.064	0.001565	180	0.001565	180

Table S38: Np(VI)-Morph phonon mode analysis in the spectral window of 600-1200 cm-1. Red modes correspond to neptunyl symmetric (ν_1) and blue modes correspond to neptunyl asymmetric (ν_3) stretches.

	Np1		Np2	
**Energy (cm^{-1})**	**s^n**	**θ^n**	**s^n**	**θ^n**
615.8863	0.012508	180	0.012508	180
623.5143	0.037652	0	0.037652	0
657.5045	0.009651	0	0.009651	0

662.1793	0.023204	180	0.023204	180
692.6836	0.030048	0	0.030048	0
693.9053	0.020636	180	0.020636	180
726.839	0.005435	0	0.005435	0
749.4474	0.023607	180	0.023607	180
750.2141	0.010157	180	0.010157	180
783.6166	0.014044	180	0.014044	0
801.0158	0.236993	0	0.336993	180
811.9317	0.233993	0	0.317555	180
826.0812	0.013582	0	0.013582	0
827.0039	0.016374	180	0.016374	180
840.436	0.021512	0	0.021512	0
855.9228	0.010104	180	0.010104	180
881.1854	0.059532	0	0.059532	0
888.9223	0.036058	0	0.036058	0
907.3669	0.024688	180	0.024688	180
922.0083	0.004447	180	0.004447	180
930.5229	0.047981	0	0.047981	0
935.6199	0.044649	0	0.044649	0
956.8696	0.008251	180	0.008251	180
960.2215	0.014679	180	0.014679	180
972.108	0.110631	0	0.110631	0
972.2	0.018252	180	0.018252	180
991.7753	0.187631	0	0.187631	0
992.2909	0.01302	180	0.01302	180
992.6055	0.040928	0	0.040928	0
1033.711	0.016854	180	0.016854	180
1038.981	0.054404	0	0.054404	0
1048.862	0.008295	180	0.008295	180
1060.881	0.023608	0	0.023608	0
1099.698	0.027373	0	0.027373	0
1105.561	0.02085	0	0.02085	0
1105.897	0.008726	180	0.008726	180
1126.267	0.014359	180	0.014359	180
1142.635	0.011708	180	0.011708	180
1148.859	0.033554	0	0.033554	0
1161.001	0.029503	0	0.029503	0
1165.052	0.010499	0	0.010499	0
1166.787	0.009644	180	0.009644	180
1172.593	0.006639	0	0.006639	0
1200.932	0.015078	180	0.015078	180
1207.871	0.019836	0	0.019836	0

Table S39: **Np(VI)-Apyr** phonon mode analysis in the spectral window of 600-1200 cm^{-1}. Red modes correspond to neptunyl symmetric (ν_1) and blue modes correspond to neptunyl asymmetric (ν_3) stretches.

	**Np1**	
**Energy (cm^{-1})**	**s^n**	**θ^n**
639.085045	0.0045349	180
640.640593	0.0035301	0
685.378225	0.0039358	0
686.546231	0.0133909	180

706.288058	0.0033789	0
710.764132	0.0027948	180
761.502968	0.0700414	180
763.100958	0.0262082	0
814.10286	0.0390723	180
820.57802	0.3203906	180
822.677183	0.0087777	0
842.153624	0.0096131	0
844.068109	0.0510624	180
850.501702	0.0135838	0
851.76056	0.030135	180
951.095105	0.006537	0
953.718271	0.0049406	180
966.474997	0.0135973	180
966.858868	0.0023452	0
993.8422	0.0038513	180
997.147733	0.108279	0
1002.05462	0.5178296	0
1039.31288	0.0450202	0
1041.12752	0.0009683	180
1042.95261	0.0017883	180
1044.54708	0.0022855	0
1107.48041	0.0145949	0
1110.03431	0.003575	180
1187.28676	0.0030325	0
1189.72528	0.002402	180

Table S40: **Np(VI)-Imi** phonon mode analysis in the spectral window of 600-1200 cm^{-1}. Red modes correspond to neptunyl symmetric (ν_1) and blue modes correspond to neptunyl asymmetric (ν_3) stretches.

	Np1		Np2	
**Energy (cm^{-1})**	**s^n**	**θ^n**	**s^n**	**θ^n**
620.4723	0.002425	0	0.002792	0
621.6766	0.001927	0	0.000713	0
622.3883	0.000508	180	0.003477	180
624.794	0.000475	0	0.003729	0
625.2005	0.003147	180	0.001564	180
627.8896	0.002443	0	0.002554	0
628.5607	0.002953	180	0.001699	180
630.4684	0.003507	180	0.001441	180
751.1083	0.012401	180	0.001241	180
751.328	0.004473	0	0.002217	0
756.9682	0.010205	180	0.001699	180
762.634	0.001885	0	0.007671	0
785.1656	0.002902	0	0.003539	0
787.1112	0.08719	180	0.037251	180
788.8431	0.007451	0	0.005634	0
792.8241	0.00028	180	0.05589	180
799.204	0.252491	180	0.017074	180
799.5489	0.005886	0	0.018751	0
805.8882	0.018217	0	0.007457	0
806.1233	0.065315	180	0.032011	180

807.7715	0.119579	180	0.039019	180
825.9324	0.045372	180	0.389493	180
871.237	0.0025	0	0.010526	0
876.1626	0.002065	0	0.01225	0
876.354	0.000869	180	0.003828	180
881.2222	0.001641	0	0.013601	0
883.9373	0.013198	180	0.010131	180
886.0442	0.009844	0	0.017056	0
887.6331	0.004114	180	0.031944	180
889.7614	0.017898	180	0.008015	180
901.8628	0.021674	0	0.007307	0
904.9346	0.003453	180	0.001545	180
905.4916	0.015006	0	0.003289	0
906.2785	0.004633	180	0.009034	180
921.3004	0.008241	0	0.017849	0
921.7068	0.00323	180	0.011362	180
921.8417	0.004806	0	0.010381	0
923.4036	0.00919	180	0.007307	180
982.7314	0.606532	0	0.016061	0
1009.615	0.014998	0	0.572287	0
1036.58	0.000938	180	0.002731	180
1037.647	0.026686	0	0.011824	0
1038.117	0.00257	180	0.004569	180
1039.298	0.017799	0	0.03593	0
1083.696	0.00318	0	0.005467	0
1083.971	0.000643	180	0.00607	180
1085.197	0.007604	0	0.02762	0
1087.352	0.000833	180	0.000541	180
1117.087	0.004008	180	0.001734	180
1119.843	0.004496	0	0.010298	0
1120.192	0.016698	0	0.003929	0
1125.265	0.006128	180	0.002958	180
1143.775	0.00528	180	0.001064	180
1147.523	0.018188	0	0.005372	0
1150.775	0.016076	0	0.006386	0
1153.565	0.004797	180	0.001359	180
1195.77	0.003642	0	0.007857	0
1196.851	0.00127	0	0.006179	0
1198.166	0.002755	180	0.00098	180

Table S41: **Np(VI)-Pipz** phonon mode analysis in the spectral window of 600-1200 cm^{-1}. Red modes correspond to neptunyl symmetric (ν_1) and blue modes correspond to neptunyl asymmetric (ν_3) stretches.

	**Np1**	
**Energy (cm^{-1})**	**s^n**	**θ^n**
771.0374	0.602601	180
802.6136	0.035913	180
815.26	0.011681	180
842.2536	0.053128	180
859.3202	0.028534	0
863.5934	0.025927	0
920.6181	0.122065	0

943.4261	0.496432	0
986.1317	0.076319	0
1027.275	0.011473	180
1039.05	0.018939	180
1049.187	0.005261	0
1069.992	0.015205	0
1137.593	0.002071	180
1171.239	0.020627	0
1186.099	0.001283	180

Table S42: **Np(VI)-Pyr** phonon mode analysis in the spectral window of 600-1200 cm^{-1}. Red modes correspond to neptunyl symmetric (ν_1) and blue modes correspond to neptunyl asymmetric (ν_3) stretches.

	Np1		Np2	
**Energy (cm^{-1})**	**s^n**	**θ^n**	**s^n**	**θ^n**
604.6364	0.001427	0	0.002946	0
605.2614	0.000963	180	0.002291	180
605.4518	0.002397	0	0.001908	0
607.0321	0.000847	180	0.002858	180
628.7396	0.000749	0	0.000828	0
629.3489	0.000363	180	0.003421	180
630.3508	0.002061	0	0.00137	0
631.3743	0.000384	180	0.003093	180
668.2724	0.001718	0	0.004334	0
674.5554	0.004566	0	0.003806	0
674.6586	0.00308	180	0.002626	180
678.531	0.00477	180	0.006994	180
728.5061	0.002524	0	0.001934	0
732.934	0.00615	180	0.015553	180
746.077	0.001727	0	0.006352	0
751.3049	0.004711	180	0.014991	180
800.8199	0.647187	180	0.06177	180
817.2526	0.046871	180	0.540623	180
851.6137	0.016962	180	0.023639	180
855.1761	0.00475	0	0.006949	0
860.9427	0.002194	180	0.008015	180
863.0368	0.002916	0	0.014399	0
866.383	0.002579	0	0.014426	0
866.3965	0.010151	180	0.010346	180
889.0449	0.007376	180	0.019075	180
891.1879	0.021195	0	0.029692	0
967.6054	0.005623	0	0.05467	0
970.8684	0.002901	180	0.006981	180
977.3899	0.127458	0	0.058279	0
981.3539	0.000917	180	0.010383	180
982.8002	0.325624	0	0.037965	0
984.3256	0.001926	180	0.004432	180
985.1512	0.050642	0	0.053486	0
986.3989	0.02058	0	0.00102	0
989.966	0.003818	180	0.006029	180
1001.932	0.001782	0	0.250846	0
1006.558	0.002719	180	0.001601	180

1006.729	0.039432	0	0.037886	0
1009.756	0.000468	180	0.002284	180
1011.95	0.018969	0	0.165278	0
1023.814	0.013259	0	0.026208	0
1024.202	0.001195	180	0.001089	180
1024.67	0.006333	0	0.029604	0
1025.802	0.001688	180	0.000557	180
1027.952	0.001664	180	0.001181	180
1028.803	0.002162	0	0.025622	0
1030.071	0.006385	0	0.007142	0
1030.301	0.001208	180	0.000256	180
1044.314	0.015453	0	0.015582	0
1044.377	0.003625	180	0.001319	180
1048.634	0.002126	180	0.001237	180
1048.892	0.009697	0	0.00528	0
1052.686	0.014384	0	0.008973	0
1054.174	0.011076	0	0.01305	0
1054.325	0.002392	180	0.00032	180
1054.575	0.003858	180	0.001824	180
1133.863	0.003981	180	0.002405	180
1135.089	0.017976	0	0.010176	0
1144.373	0.010067	180	0.003158	180
1144.585	0.023315	0	0.005281	0
1168.449	0.006752	0	0.005505	0
1168.512	0.003473	180	0.000825	180
1173.523	0.00391	180	0.000914	180
1176.563	0.014462	0	0.002065	0

Table S43: **Np(VI)-K** phonon mode analysis in the spectral window of 600-1200 cm^{-1}. Red modes correspond to neptunyl symmetric (ν_1) and blue modes correspond to neptunyl asymmetric (ν_3) stretches.

	**Np1**	
**Energy (cm^{-1})**	**s^n**	**θ^n**
613.9178	0.040886	180
815.8881	0.817064	180
981.0589	0.949027	0

Table S44: **Np(VI)-Rb** phonon mode analysis in the spectral window of 600-1200 cm^{-1}. Red modes correspond to neptunyl symmetric (ν_1) and blue modes correspond to neptunyl asymmetric (ν_3) stretches.

	**Np1**	
**Energy (cm^{-1})**	**s^n**	**θ^n**
815.6299	0.835437	180
984.6784	0.952808	0

Table S45: **Np(VI)-Cs** phonon mode analysis in the spectral window of 600-1200 cm^{-1} Red modes correspond to neptunyl symmetric (ν_1) and blue modes correspond to neptunyl asymmetric (ν_3) stretches.

	Np1		Np2	
**Energy (cm^{-1})**	s^n	θ^n	s^n	θ^n
809.4944	0.462251	180	0.462251	180
814.4682	0.466128	180	0.466128	180
994.6889	0.493789	0	0.493789	0
1000.178	0.486855	0	0.486855	0

8. Reference

(1) Anisimov, V. I.; Gunnarsson, O. Density-functional calculation of effective Coulomb interactions in metals. *Physical Review B* **1991**, *43* (10), 7570-7574. DOI: 10.1103/PhysRevB.43.7570.
(2) Anisimov, V. I.; Zaanen, J.; Andersen, O. K. Band theory and Mott insulators: Hubbard U instead of Stoner I. *Physical Review B* **1991**, *44* (3), 943-954. DOI: 10.1103/PhysRevB.44.943.
(3) Anisimov, V.; Aryasetiawan, F.; Lichtenstein, A. First-Principles Calculations of the Electronic Structure and Spectra of Strongly Correlated Systems: The LDA+ U Method. *Journal of Physics: Condensed Matter* **1997**, *9*, 767. DOI: 10.1088/0953-8984/9/4/002.
(4) Neidig, M. L.; Clark, D. L.; Martin, R. L. Covalency in f-element complexes. *Coordination Chemistry Reviews* **2013**, *257* (2), 394-406. DOI: https://doi.org/10.1016/j.ccr.2012.04.029.
(5) Denning, R. G. Electronic Structure and Bonding in Actinyl Ions and their Analogs. *The Journal of Physical Chemistry A* **2007**, *111* (20), 4125-4143. DOI: 10.1021/jp071061n.
(6) Rajapaksha, H.; Mason, S. E.; Augustine, L. J.; Forbes, T. Guiding Principles for the Rational Design of Hybrid Materials: Use of DFT Methodology for Evaluating Non-Covalent Interactions in a Uranyl Tetrahalide Model System. *Angewandte Chemie International Edition* **2023**, *n/a* (n/a), e202305073. DOI: https://doi.org/10.1002/anie.202305073.
(7) NEA. *Chemical Thermodynamics of Neptunium and Plutonium*; OECD Publishing, 2001. DOI: 10.1039/9781782624028.
(8) The NBS tables of chemical thermodynamic properties : selected values for inorganic and C_1 and C_2 organic substances in SI units. Wagman, D. D., Ed.; American Chemical Society and the American Institute of Physics for the National Bureau of Standards: New York ;, 1982.
(9) Cahill, C. L.; Deifel, N. P.; Reusser, D.; Zhang, L.; Navrotsky, A. Thermochemical properties of U(VI) hybrid materials containing uranyl tetrachloride anions. *The Journal of Chemical Thermodynamics* **2017**, *114*, 66-70. DOI: https://doi.org/10.1016/j.jct.2017.05.009.
(10) Parker, V. B. *Thermal properties of aqueous uni-univalent electrolytes*; US Government Printing Office Washington, DC, 1965.
(11) Manz, T. A.; Limas, N. G. Introducing DDEC6 atomic population analysis: part 1. Charge partitioning theory and methodology. *RSC Advances* **2016**, *6* (53), 47771-47801, 10.1039/C6RA04656H. DOI: 10.1039/C6RA04656H.

(12) Limas, N. G.; Manz, T. A. Introducing DDEC6 atomic population analysis: part 2. Computed results for a wide range of periodic and nonperiodic materials. *RSC Advances* **2016**, *6* (51), 45727-45747, 10.1039/C6RA05507A. DOI: 10.1039/C6RA05507A.
(13) Manz, T. A. Introducing DDEC6 atomic population analysis: part 3. Comprehensive method to compute bond orders. *RSC Advances* **2017**, *7* (72), 45552-45581, 10.1039/C7RA07400J. DOI: 10.1039/C7RA07400J.
(14) Limas, N. G.; Manz, T. A. Introducing DDEC6 atomic population analysis: part 4. Efficient parallel computation of net atomic charges, atomic spin moments, bond orders, and more. *RSC Advances* **2018**, *8* (5), 2678-2707, 10.1039/C7RA11829E. DOI: 10.1039/C7RA11829E.
(15) Spano, T. L.; Shields, A. E.; Barth, B. S.; Gruidl, J. D.; Niedziela, J. L.; Kapsimalis, R. J.; Miskowiec, A. Computationally Guided Investigation of the Optical Spectra of Pure β-UO3. *Inorganic Chemistry* **2020**, *59* (16), 11481-11492. DOI: 10.1021/acs.inorgchem.0c01279.
(16) Augustine, L. J.; Rajapaksha, H.; Pyrch, M. M. F.; Kasperski, M.; Forbes, T. Z.; Mason, S. E. Periodic Density Functional Theory Calculations of Uranyl Tetrachloride Compounds Engaged in Uranyl–Cation and Uranyl–Hydrogen Interactions: Electronic Structure, Vibrational, and Thermodynamic Analyses. *Inorganic Chemistry* **2023**, *62*, 372-380. DOI: 10.1021/acs.inorgchem.2c03476.
